# Effect modification of the association between comorbidities and severe course of COVID-19 disease by age of study participants: a systematic review and meta-analysis

**DOI:** 10.1186/s13643-021-01732-3

**Published:** 2021-06-30

**Authors:** Nathalie Verónica Fernández Villalobos, Jördis Jennifer Ott, Carolina Judith Klett-Tammen, Annabelle Bockey, Patrizio Vanella, Gérard Krause, Berit Lange

**Affiliations:** 1 Department of Epidemiology, Helmholtz Centre for Infection Research (HZI), Inhoffenstraße 7, 38124 Braunschweig, Germany; 2 PhD Programme Epidemiology, Helmholtz Centre for Infection Research (HZI), Inhoffenstraße 7, 38124 Braunschweig-Hannover, Germany; 3 Hannover Medical School, Hannover, Germany; 4 German Center for Infection Research (DZIF), Braunschweig, Germany; 5 Department of Medicine II, Division of Infectious Diseases, University Hospital Freiburg, Freiburg, Germany; 6 Chair of Empirical Methods in Social Science and Demography, University of Rostock, Rostock, Germany; 7 TWINCORE GmbH, Centre for Experimental and Clinical Infection Research, Hannover, Germany

**Keywords:** Comorbidity, COVID-19-associated hospitalisation, COVID-19-associated mortality, Effect modification, Interaction, Meta-analyses

## Abstract

**Background:**

Comprehensive evidence synthesis on the associations between comorbidities and behavioural factors with hospitalisation, intensive care unit (ICU) admission, and death due to COVID-19 is required for deriving national and international recommendations on primary targets for non-pharmacological interventions (NPI) and vaccination strategies.

**Methods:**

We performed a rapid systematic review and meta-analysis on studies and publicly accessible data to quantify associations between predisposing health conditions, demographics, behavioural factors on the one hand and hospitalisation, ICU admission, and death from COVID-19 on the other hand. We provide ranges of reported and calculated effect estimates and pooled relative risks derived from a meta-analysis and meta-regression.

**Results:**

Seventy-five studies were included in qualitative and 74 in quantitative synthesis, with study populations ranging from 19 to 44,672 COVID-19 cases. The risk of dying from COVID-19 was significantly associated with cerebrovascular [pooled relative risk (RR) 2.7 (95% CI 1.7–4.1)] and cardiovascular [RR 3.2 (CI 2.3–4.5)] diseases, hypertension [RR 2.6 (CI 2.0–3.4)], and renal disease [RR 2.5 (CI 1.8–3.4)], with high heterogeneity in pooled estimates, partly but not solely explained by age of study participants. For some comorbidities, our meta-regression showed a decrease in effect on the severity of disease with a higher median age of the study population. Compared to death, associations between several comorbidities and hospitalisation and ICU admission were less pronounced.

**Conclusions:**

We obtained robust estimates on the magnitude of risk for COVID-19 hospitalisation, ICU admission, and death associated with comorbidities, demographic, and behavioural risk factors and show that these estimates are modified by age of study participants. This interaction is an important finding to be kept in mind for current vaccination strategies and for the protection of individuals with high risk for a severe COVID-19 course.

**Supplementary Information:**

The online version contains supplementary material available at 10.1186/s13643-021-01732-3.

## Introduction

Various factors determine the risk of a severe course of COVID-19 disease and COVID-related deaths. Some of them are demographic in nature, such as age and sex, and others have to do with diagnosed conditions such as diabetes and hypertension [1–5]. Furthermore, behavioural and occupational risk factors have also been discussed [6–9]. Accordingly, some non-pharmacological interventions are aimed towards protecting affected population groups like the elderly [10].

The definition of exact target groups for health measures other than the elderly [11, 12] has often been vague. A part of the reason for this is that research evidence for such predisposing factors is based on incomparable data sources, indicators and calculations, and denominators. Estimates are further challenged not only by the interrelation between co-morbidities and disease severity, but also their interaction with age [13–15]. In most studies, effect estimates for factors experienced by patients are missing, as they focus on data description in the form of clinical case series [16–18].

In order to prioritise current vaccination strategies against COVID-19 infection, national and international policies require information on major risk groups. An evidence synthesis to quantify the risk of individual predisposing factors and their interactions with regard to disease severity is needed. The existing systematic reviews on that subject have been published as pre-print or as peer-reviewed publications to date [6–8, 19–23]. Some of these focus on one association to a particular endpoint, e.g., cardiovascular morbidity and severity of the course of the disease but do not quantify the actual risk of patients with these comorbidities in relation to important health outcomes [24–26]. Some assess several comorbidities [4, 23] but do not cover many studies, as they have been conducted at a very early stage of the pandemic. Importantly, although age is studied as a risk factor, the interaction of age with comorbidities has rarely been studied (this information is available in the Supplement, Table 1). To our knowledge, no existing review provides comparative relative risk (RR) measures of the major predisposing conditions, using both crude data and reported RR measures, while taking the age structure—not just as a potential confounder but also as a potential effect modifier—of the respective study population into account.

We conducted a meta-analysis on risk for COVID-related hospitalisation, intensive care unit (ICU) admission, and death and its association with demographics, comorbidities, and behavioural factors. We extracted and included crude study data to understand the magnitude of the effect of comorbidities and other factors on COVID-19 health outcomes. By focussing on these outcomes, our objective was to generate evidence for prioritising health measures for vulnerable population groups. Our results were made publicly made available (https://www.medrxiv.org/content/10.1101/2020.07.30.20165050v1). With this communication, we aim to focus not only on the relation between individual co-morbidities and disease severity (e.g., as measured by ICU admission) but also further identify potential interactions with age.

## Material and methods

### Search strategy

We performed a systematic review (registration number in PROSPERO CRD42020190548), following PRISMA and MOOSE guidelines [27, 28], in MEDLINE, bioRXiv, and MedRXiv, searching for publications on COVID-19 and risk groups for severe or lethal disease outcomes (search terms “novel coronavirus”, “COVID-19”, “SARS-CoV-2”). We further applied the snowball method [29] to available systematic reviews to identify additional evidence. The available and accessible reviews are listed in Supplement, table 1. The literature search includes reports up to May 28, 2020. Additionally, we include reports from other publicly available sources, namely national (public) health institutions, and data repositories.

### Inclusion and exclusion criteria

We included reports if (a) patients had COVID-19, either confirmed microbiologically or clinically (population); (b) information on COVID-19 outcome was reported as either death (hospital or after a defined follow-up time), ICU admission (both ICU and intermediate care), or hospitalisation (clinical description) (outcome); and (c) at least one comorbidity, risk factor or behavioural factor was described and if the number of patients with/without outcome was reported according to the respective factor (exposure and comparison). Eligible study designs were cohort studies, cross-sectional studies, case series, and clinical trials. Languages included were English, Spanish, Italian, French, or German.

### Data extraction

We extracted relevant variables as specific as provided (age, date, etc.) and according to the main stratification variable, either comorbidity or behavioural risk factor, author, URL link, country, data source, age range, study time-frame, baseline population group, outcome (mortality, severity, or other), number of individuals in the risk group, total sample, number of individuals among risk group with the outcome, total number of individuals with the outcome, and effect measures of association reported as well as RRs computed automatically.

The outcomes were severity during the course of COVID-19 disease in terms of hospitalisation, admission to ICU, and death. We also included another category “composite endpoint” for those studies that were not possible to separate into the previously defined outcomes. The definition for this category is studies that reported admission to an ICU, the use of mechanical ventilation, the use of extracorporeal membrane oxygenation (ECMO), or death.

“Risk groups” were those with comorbidities (International Classification of Diseases 11th Revision (ICD-11)) [30]. We extracted the five most common comorbidities as well as behavioural, occupational, or demographic factors per included study. Smoking was grouped into current or former smokers.

For data from research reports, we did random plausibility checks and plotted RRs with ranges. A researcher not involved in the data extraction (AB) double-checked 20% of included studies and compared extracted numbers with original reports.

For publicly available data, we extracted data for seven countries on the age of confirmed COVID-19 cases, hospitalisations, ICU admissions, and deaths. For the United States of America (USA), Spain, and France, we additionally extracted mortality data, distinguished by comorbidities, which we used to estimate RR of death among cases, or in the case of France, among hospitalisations.

### Risk of bias

We assessed the risk of bias using an adapted version of the ROBINS-I tool [31] for non-randomised studies. We analysed the studies in terms of bias due to confounding, selection of participants and follow-up, misclassification of exposure, missing data, measurement of outcome, or reporting. We measured the risk scales as low, moderate, and high.

### Data analysis

#### Descriptive

We display ranges of reported estimates of association [odds ratios (ORs), hazard ratios (HRs), and RRs] for the health outcomes from included studies and calculate RRs for each risk group and for each outcome, based on crude and absolute data from the studies. Strata of outcomes that reported “zero” were excluded from the analysis, as this gives an invalid statistical estimate of the underlying risks.

For data from publicly available sources, we computed point estimates of the RRs to severe health status, like hospitalisation or death; if possible, stratified by age groups and sex with 95% confidence intervals (95% CIs). In addition, we estimated RRs of death among three age groups and two sexes for cases in Spain and hospitalised cases in France.

#### Meta-analysis and meta-regression

We assessed heterogeneity visually in forest plots and by assessing the percentage of variance overstudies I^2^. Due to the difference between populations and observed heterogeneity, we performed a random-effects meta-analysis for pooled RRs. For those risk groups with considerable heterogeneity (>75%), we performed subgroup analyses to further investigate reasons for heterogeneity according to the Cochrane Handbook for Systematic Reviews of Interventions [32]. Within meta-regression, we assessed effect modification by age on RRs of included comorbidities or other risk factors.

## Results

**Fig. 1:**
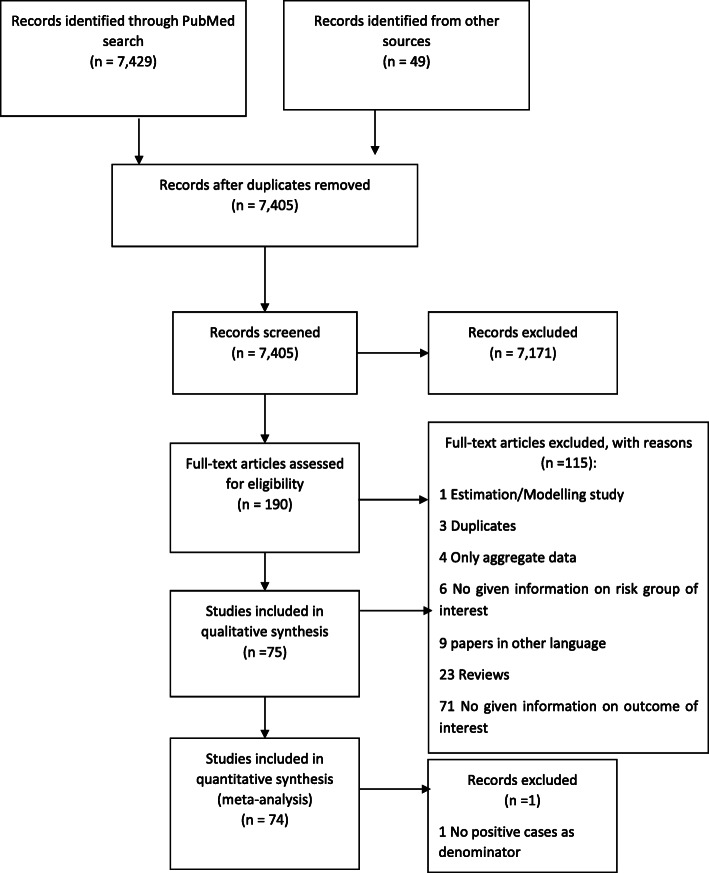
Flow chart of study selection based on the rapid review

We identified a total of 7429 records. We retrieved 190 of them for full-text screening and 75 studies met the inclusion criteria (Fig. 1). All of them were used for our qualitative analysis, and 74 for the quantitative analysis. One study was excluded as the number of confirmed COVID-19 cases was reported [33].

The majority of reports (*n*=66) were from China, followed by the USA (*n*=5). Studies were based on medical or clinical records (*n*=69) or official reported data (*n*=4) and were conducted between late December 2019 and April 2020 with follow-up of 5–30 days. Endpoints were hospitalisation (*n*= 43), admission to ICU (*n*=17), and death (*n*=26; mostly within 30 days or in-hospital deaths). Three studies had composite endpoints, three others reported multiple endpoints.

The sample sizes were between 19 and 44,672 confirmed COVID-19 cases and individuals were aged between 33 and 82 years; in seven studies children were also included (Supplement, Table 2). To our knowledge and after multiple reviews, most studies were published in peer-reviewed journals, except one that appears still as a preprint (Liu et al. (2020) Clinical features and progression of acute respiratory distress syndrome in coronavirus disease 2019) [34].

### Risk of bias assessment

We assessed the risk of bias due to (a) confounding, (b) selection, (c) misclassification, (d) missing data, and (e) measurement of outcome. Confounding was moderate in most studies as either adjusted estimates or age information was provided. Selection bias was mostly low to moderate as was misclassification. A high risk of bias was found for 45 studies due to non-reporting or missing data. In several studies, the source of data or definition of the outcome was unclear and several reported results in selective subgroups (Supplement, Table 3).

### Reported and calculated associations

Measures reported were ORs (14 studies), HRs (9 studies), and RRs (one study); an additional study included cases reported from official data. We calculated RRs from crude study data for (a) hospitalisation, (b) admission to ICU, and (c) death (within 30 days or within the hospital) for all 74 studies included in the quantitative analysis (Table 1).

**Table 1:** Association in original studies and own calculations of relative risks by COVID-19 outcomes and by risk

Risk group	Outcome	Number of studies reporting	Reported measure of association (95 %CI)	Number of studies Calculated RR	Calculated relative risk ranges (95 %CI)	Pooled analysis RR (95% CI)
**Comorbidities**						
Asthma	Death			1	0.9 (0.3–2.4)	0.9 (0.3–2.4)
	Hospitalisation			2	1.2 (0.6–2.5)–1.3 (0.5–3.6)	1.2 (0.7–2.2)
	ICU admission	1		1	1.1 (0.7–1.6)	1.1 (0.7–1.6)
Cancer	Death	1	RR: 2.9 (1.3–6.4)	7	0.7 (0.1–4.9)–4.8 (1.1–21.5)	2.0 (1.4–2.8)
	Hospitalisation			12	0.8 (0.1–4.4)–4.4 (1.0–19.5)	1.5 (1.2–1.8)
	ICU admission	1	OR: 5.4 (1.8–16.2)	5	1.4 (0.7–3.0)–4.9 (2.7–9.0)	2.3 (1.3–4.0)
Cardiovascular disease	Death	4	RR: 6.8 (5.4–8.4)	15	1.3 (0.2–8.9)–6.7 (5.4–8.4)	3.3 (2.3–4.5)
			OR: 1.2 (0.2–7.8) -2.1 (0.3–17.8)			
			HR: 1.9 (1.1–3.3)			
	Hospitalisation	2	OR: 2.4 (1.5–3.9)	23	0.7 (0.1–3.9)–3.9 (1.7–8.8)	2.0 (1.6–2.4)
			HR: 0.6 (0.1–3.6)			
	Composite			1	1.6 (0.8–3.1)	1.6 (0.8–3.1)
	ICU admission			8	0.9 (0.4–2.1)–4.1 (3.4–4.9)	2.1 (1.3–3.2)
Cerebrovascular disease	Death	1	HR: 1.4 (0.7–2.9)	7	1.2 (0.3–4.3)–7.1 (3.3–15.4)	2.6 (1.7–4.1)
	Hospitalisation			6	1.1 (0.7–2.0)–3.4 (2.3–5.1)	2.2 (1.5–3.3)
	Composite			1	4.6 (1.9–11.0)	4.6 (1.9–11.0)
	ICU admission			4	0.8 (0.5–1.3)–3.7 (2.4–5.8)	1.9 (0.9-4.0)
Chronic obstructive pulmonary disease (COPD)	Death	2	OR: 5.4 (0.9–30.4)	6	1.9 (0.4–10.1)–6.0 (1.3–26.8)	2.4 (2.0–3.0)
			HR: 2.2 (1.1–4.5)			
	Hospitalisation			11	1.2 (0.9–1.7)–4.3 (0.8–17.1)	2.0 (1.5–2.6)
	Composite			1	0.9 (0.2–5.0)	0.9 (0.2–5.0)
	ICU admission			4	1.0 (0.4–2.8)–10.6 (6.2–16.1)	2.4 (0.6–9.8)
Chronic renal failure	Death			3	1.9 (0.4–10.1)–2.6 (1.8–3.6)	2.5 (1.8–3.4)
	Hospitalisation			9	0.8 (0.3–2.5)–3.3 (2.8–3.9)	1.6 (1.0–2.6)
	ICU admission			4	0.8 (0.4–1.9)–4.6 (3.6–5.8)	2.1 (0.9–4.9)
Diabetes mellitus	Death	5	RR: 4.5 (3.5–5.6)	18	1.1 (0.6–1.9)–4.5 (1.9–10.6)	2.2 (1.7–2.9)
			OR: 2.9 (1.4–6.1)–4.9 (1.3–18.2)			
			HR: 1.1 (0.6–2.1)–1.7 (0.3–8.2)			
	Hospitalisation	4	OR: 0.7 (0.1–8.3)–OR: 4.7 (0.7–32.4) HR: 1.6 (1.0–2.4)–HR: 1.7 (0.3–8.2)	30	0.7 (0.2–2.4)–4.3 (2.4–7.7)	1.8 (1.5–2.2)
	Composite			1	1.4 (0.7–2.8)	1.4 (0.7–2.8)
	ICU admission	3	OR: 1.6 (0.4–5.8)–2.1 (0.7–6.2)	12	0.3 (0.1–2.3)–4.6 (2.8–7.5)	1.9 (1.4–2.6)
			HR: 2.3 (1.4–4.1)			
Diseases of liver	Death			3	0.9 (0.1–5.9)–4.8 (1.1–21.5)	1.9 (0.6–6.4)
	Hospitalisation			10	0.8 (0.1–4.3)–11.3 (4.6–27.6)	1.7 (1.0–2.9)
Diseases of the digestive system	Death			1	0.8 (0.1–6.1)	0.8 (0.1–6.1)
	Hospitalisation			3	0.7 (0.2–2.2)–2.0 (0.7–5.2)	1.2 (0.9–1.5)
	ICU admission			1	0. 8 (0.4–1.5)	0.8 (0.4–1.5)
Endocrine diseases	Death			1	1.2 (0.4–3.9)	1.2 (0.4–3.9)
	Hospitalisation			1	1.4 (1.2–1.5)	1.4 (1.2–1.5)
Hypertension	Death	6	RR: 4.5 (3.7–5.5)	17	1.1 (0.2–6.4)–8.1 (2.9–22.3)	2.7 (2.1–3.4)
			OR: 1.1 (0.3–4.6)–3.1 (1.6–5.9)			
			HR: 1.5 (0.9–2.4)–1.7 (0.9–3.1)			
	Hospitalisation	5	OR 2.7 (1.3–5.6)–4.4 (1.0–18.9)	29	0.8 (0.3–1.9)–4.4 (2.6–7.4)	1.8 (1.6–2.1)
			HR: 1.6 (0.4–5.8)–HR: 1.6 (1.1–2.3)			
	Composite			2	1.3 (0.7–2.4)–3.2 (2.0–5.1)	2.1 (0.9–4.9)
	ICU admission	3	OR: 0.5 (0.1–1.7)–2.3 (0.9–5.8)	9	0.9 (0.6–1.3)–3.1 (1.8–5.4)	1.4 (1.1–1.7)
			HR:1.8 (1.1–2.9)			
Immunocompromised condition	Hospitalisation			1	1.7 (1.4–2.1)	1.7 (1.4–2.1)
	ICU admission			1	2.6 (1.9–3.5)	2.6 (1.9–3.5)
Mycobacterial diseases	Hospitalisation			1	0.9 (0.4–1.9)	0.9 (0.4–1.9)
Other diseases or unspecified	Death	1	OR: 1.5 (1.0–2.2)	2	1.6 (1.1–2.4)–1.9 (0.9–4.3)	1.7 (1.2–2.4)
	Hospitalisation	2	OR: 2.8 (0.8–10.1)	6	0.9 (0.3–2.7)–6.0 (3.5–10.4)	2.9 (1.6–5.1)
			HR: 3.9 (1.9–7.9)			
	ICU admission			1	2.5 (1.3–4.9)	2.5 (1.3–4.9)
Other respiratory diseases	Death			7	0.8 (0.3–2.2)–3.4 (2.4–4.9)	2.2 (1.5–3.0)
	Hospitalisation			7	0.9 (0.5–1.7)–2.9 (1.0–8.2)	1.4 (1.1–1.8)
	ICU admission			3	0.4 (0.2–1.1)–2.6 (2.1–3.2)	1.3 (0.6–2.9)
**Demographic, behavioural, and occupational factors**						
Healthcare profession	Death			2	0.1 (0.0–0.8)–0.1 (0.1–0.3)	0.1 (0.1–0.3)
	Hospitalisation			3	0.1 (0.0–0.3)–0.8 (0.3–2.2)	0.3 (0.1–0.9)
Male sex	Death	4	RR: 1.7 (1.5–1.9)	19	0.7 (0.1–7.5)–4.7 (1.4–15.1)	1.4 (1.3–1.6)
			OR: 1.5 (1.1–1.9)–7.2 (1.3–40.2)			
			HR: 0.6 (0.3–1.1)			
	Hospitalisation	5	OR: 1.5 (0.6–3.6)–4.4 (1.0–18.9)	36	0.8 (0.4–1.3)–3.5 (1.7–7.1)	1.3 (1.2–1.5)
			HR: 0.9 (0.9–0.9)–1.7 (1.1–2.8)			
	Composite	1	OR: 1.9 (0.5–7.2)	2	1.5 (0.8–2.8)–1.8 (0.6–5.5)	1.6 (0.9–2.7)
	ICU admission	3	OR: 1.1 (0.3–3.4)–2.8 (1.0–7.9)	11	0.7 (0.3–1.6)–2.0 (0.5–77)	1.3 (1.1–1.4)
			HR: 1.5 (0.9–2.4)			
Obesity	Hospitalisation	1	OR: 6.3 (1.2–34.5)	2	2.1 (1.1–4.2)–4.0 (1.0–15.6)	2.4 (1.3–4.4)
	ICU admission	1	OR: 5.4 (1.1–25.6)–9.9 (1.4–71.7)	2	1.4 (1.0–1.8)–1.5 (0.9–2.3)	1.4 (1.1–1.8)
Smoking	Death	1	OR: 2.2 (0.7–7.6)	4	1.2 (0.8–1.7)–8.7 (3.7–20.1)	2.6 (1.0–6.8)
	Hospitalisation	1	OR 14.3 (1.6–25.0)	17	0.4 (0.1–2.3)–5.5 (2.1–14.4)	1.5 (1.2–1.9)
	ICU admission			5	0.9 (0.5–1.8)–2.9 (1.8–4.8)	1.8 (1.1–2.9)

National primary care electronic health record data linked to in-hospital COVID-19 death data

### Hospitalisation

Two studies reported higher odds of being hospitalised due to COVID-19 for patients with cardiovascular disease and diabetes [OR 2.4; HR 1.6, respectively]. Hypertension was reported as a factor that increased the odds of being hospitalised [OR 2.7–4.4, HR 1.6; 5 studies].

Using crude data from all clinical case series that provided these numbers, we calculated that patients with cerebrovascular disease or chronic obstructive pulmonary disease (COPD) had a higher risk of hospitalisation [RR 1.1–3.4; 6 studies] [1.2–4.3; 11 studies], respectively. Regarding the association of demographic, behavioural, and occupational factors and hospitalisation, four studies reported that male patients had higher odds and hazard of being hospitalised [OR 3.7, HR 1.7], while two studies did not see evidence of an association [OR 0.5, HR 0.9].

Using the crude data, we found that male patients [RR 1.1–3.5; 29 studies], patients who smoke [1.1–5.5; 14 studies], and obese patients [2.1–4.0; 2 studies] had a higher risk of disease severity. Other studies did not find associations between being male [0.8–0.9; 7 studies] or smoking [0.4–0.7; 3 studies] and hospitalisation. Healthcare workers were found to have a lower risk of being hospitalised due to COVID-19 [RR 0.1–0.8; 3 studies] (all Table 1).

### ICU admission

Four studies reported on those patients that needed to be admitted to an ICU based on medical records. Three studies reported cancer, diabetes, or hypertension as factors that increase the odds of ICU admission [OR 5.4, HR 2.3, and HR 1.8, respectively].

Using crude numbers of patients and events provided in studies, we calculated that patients with cancer [RR 1.4–4.9; 5 studies] or COPD [1.0–10.6; 4 studies] had a high risk of being admitted to an ICU.

Regarding other risks, one study reported that male patients [OR 2.8] had higher odds of being admitted to an ICU or having invasive mechanical ventilation.

We found obesity [RR 1.4–1.5; 2 studies], male gender [RR 1.1–2.0; 9 studies], or smoking [RR of 2.4–2.9; 3 studies] to be risk factors for ICU admission. Other studies did not find associations between being male [0.7–0.9; 2 studies] or smoking [0.9; 2 studies] and ICU admission (Table 1).

### Death

Eight studies reported on patients deceased due to COVID-19, and one study included cases reported from official data.

Two studies reported cancer and COPD as comorbidities associated with death [RR 2.9 and HR: 2.2, respectively]. Patients with cardiovascular disease [HR 1.9, RR 6.8; 4 studies], diabetes [OR 2.9–4.9, RR 4.5; 5 studies], or hypertension [OR 3.1, HR of 1.6, RR of 4.5; 6 studies] had a higher risk of dying because of COVID-19.

Calculating from crude numbers of patients and events provided in study reports, we found that patients with cardiovascular disease [RR 1.3–6.7; 15 studies], cerebrovascular disease [1.2–7.1; 7 studies], COPD [1.9–6.0; 6 studies], diabetes [1.1–4.5; 18 studies], or hypertension [1.1–8.1; 17 studies] had a higher risk of dying due to COVID-19 than those patients without these comorbidities.

Regarding demographic, behavioural, and occupational factors, two studies reported that male patients had higher odds of death [OR 1.5, RR 1.7].

Based on the calculated RR, we found that males [RR 1.2–4.7; 14 studies] or smokers [RR 1.2–8.7; 4 studies] had a higher mortality risk. There was no association between being male and death [RR 0.7–0.9] or healthcare workers had a lower risk of death [RR 0.1, 2 studies] (Table 1).

#### National primary care electronic health record data linked to in-hospital COVID-19 death data

One study [33] reported their results based on primary care records for patients in England. Deaths from COVID-19 were associated with being male [HR 1.6 (95% CI 1.5–1.6)], age [HR 2.4 (2.1–2.6), HR 6.1 (5.5–6.7), and HR 20.6 (18.7–22.7) for age groups 60–69, 70–79, and >80 years of age, respectively, taking into account age group 50–59 as a reference], uncontrolled diabetes [HR 1.9 (1.8–2.0)], and severe asthma [HR 1.1 (1.0–1.3)].

### Meta-analysis and meta-regression: association of comorbidities and demographic, behavioural, and occupational factors with hospitalisation, ICU admission, and death—effect modification of age

The random-effects meta-analysis assessed the influence of comorbidities and other factors on three endpoints (hospitalisation, ICU admission, and death; Fig. 3). To assess the influence of age on these associations, we performed meta-regressions for those associations with more than 15 studies available on the influence of age on these associations. We also performed a mixed-effects meta-regression on the main comorbidities adjusted for median/mean age and gender.

### Hospitalisation

#### Comorbidities

**Fig. 2:**
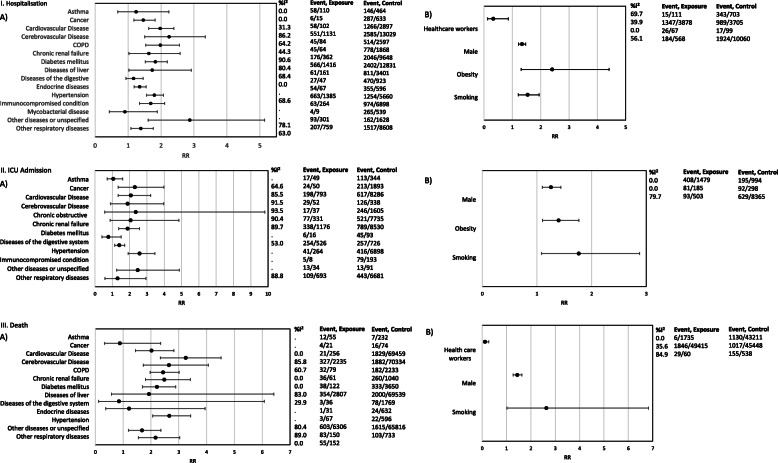
Summary effect meta-analysis of hospitalisation, ICU admission, and death. I. Hospitalisation: A. association of hospitalisation and comorbidity, B. association of hospitalisation and demographic, behavioural, and occupational. II. ICU admission: A. association of ICU admission and comorbidity, B. association of ICU admission and behavioural, demographic, and occupational factors. III. Death: A. association of death and comorbidity, B. association of death and behavioural, demographic, and occupational factors

Random-effects meta-analysis found patients with cardiovascular disease (RR 1.9, 95% CI 1.6–2.4), cerebrovascular disease (RR 2.3, 1.5–3.3), and/or diabetes mellitus (RR 1.8, 1.5–2.6) were at higher risk of hospitalisation. Other comorbidities, including chronic renal disease, chronic respiratory disease, and COPD, were correlated with higher hospitalisation rates as well. We found moderate to high heterogeneity for several of these risk factors (I^2^ 50–90%) (Fig. 2.I.A.).

#### Demographic, behavioural, and occupational factors

In pooled results from random-effect meta-analysis, obese individuals had 2.4 times the risk of being hospitalised compared to those without obesity (RR 2.4, 95% CI 1.3–4.4). Healthcare workers were less likely to be hospitalised (RR 0.3, 0.1–0.9), and males were 30% more likely to be hospitalised than females (RR 1.3, 1.2–1.5) (Fig. 2.I.B.).

### ICU admission

#### Comorbidities

The following comorbidities were associated with high risk for ICU admission (Fig. 2.II.A.) in pooled analysis: cancer (RR 2.3, 95%CI 1.3–4), diabetes mellitus (RR 1.9, 1.4–2.6), and cardiovascular conditions (RR 2.0, 1.3–3.2). Heterogeneity of pooled results was moderate to high.

#### Demographic, behavioural, and occupational factors

Obesity and smoking moderately increased the risk to being admitted to an ICU (Fig. 2.II.B.). Information on healthcare workers was insufficient to pool results.

### Death

#### Comorbidities

The highest observed RRs of death were found for cerebrovascular disease, cardiovascular disease, chronic renal disease, and hypertension (RR 2.7, 95% CI 1.7–4.0), (3.2, 2.3–4.5), (2.5, 1.8–3.4), and (2.6, 2.0–3.4), respectively (Fig. 2.III.A.).

#### Demographic, behavioural, and occupational factors

Males had risk of death due to COVID-19 1.4 times that of females (95% CI 1.3–1.6), and healthcare professionals were at lower risk of death due to COVID-19, when compared to other population groups (RR 0.12, 95% CI 0.06–0.27) (Fig. 2.III.B).

### Effect modification

#### Hospitalisation

**Fig. 3:**
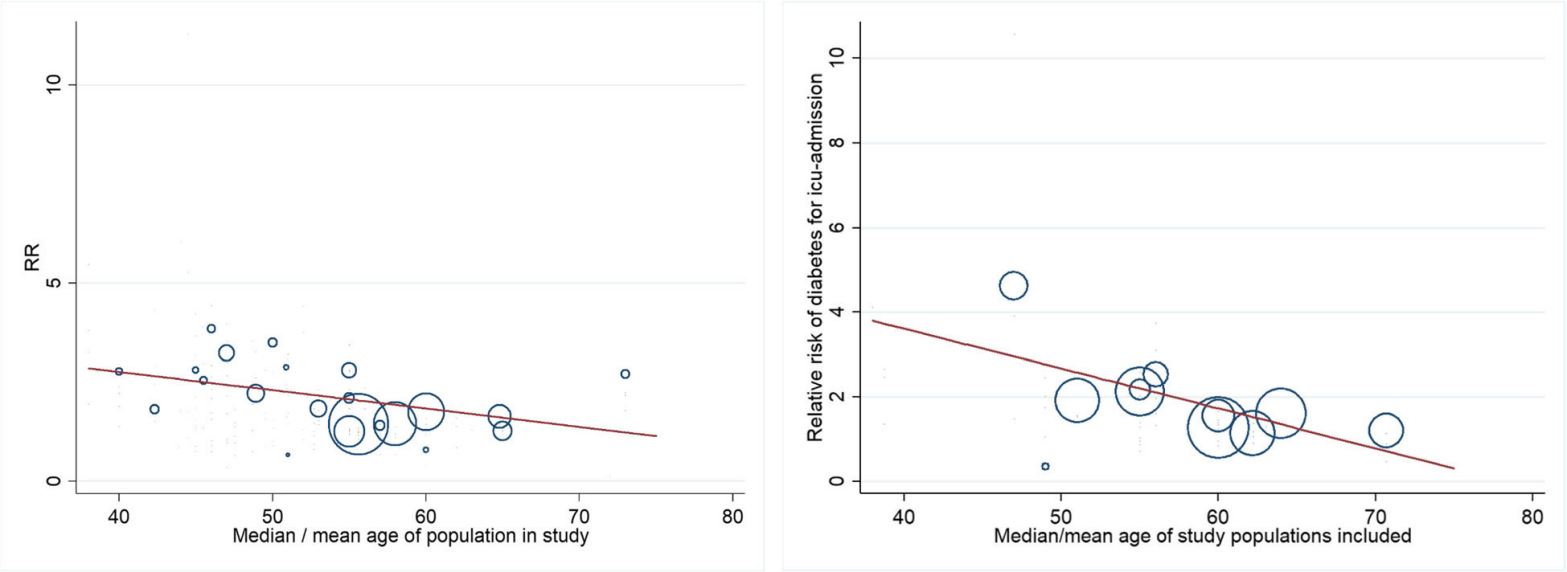
Effect modification of mean/median age of the population in the study: A. modification of the association between cardiovascular morbidity and hospitalisation, B. modification of age on the association of diabetes and ICU admission risk

Meta-regression revealed that the strength of the association between comorbidities and hospitalisation decreased with increased median or mean age of the study population [cardiovascular (coefficient −0.05, 95%CI −0.09–0.003, *p* value≈0.038) and diabetes (coefficient −0.07, 95%CI −0.1- −0.03, *p* value≈0.002)] (Fig. 3.A).

#### ICU

The age was also modifying the association with diabetes; here, the RRs for ICU admission decreased with increasing age (coefficient −0.1, 95% CI −0.2 - −0.004, *p* value≈0.042) (Fig. 3.B). We did not find effect modification of age for other risk factors like gender, hypertension, or smoking.

#### Death

Effect modification was found for the association of hypertension with dying from COVID-19 with higher relative risks in those studies with lower median/mean ages (coefficient −0.14, 95% CI −0.27 - −0.022, *p* value≈0.025). We did not find effect modification for diabetes or cardiovascular morbidity.

### Publicly available data

Based on data from Spain and France, the RR varies by age and sex. Individuals aged 70+ years were at higher risk of death and hospitalisation than younger individuals. For example, as of May 21, 2020, in Spain, a 50–69-year-old male case of COVID-19 had an estimated risk of dying 6.9 (95% CI 6.0–8.0) times that of a male below 50 years of age. Males were at higher risk of dying or witnessing a severe course of the infection than females. For instance, in Italy, a male aged 50 years or younger was estimated to have a 3.33 (2.6–4.2) higher average risk of death after infection than a woman at the same age (Tables 2 and 3).

**Table 2:** Relative risk estimates for COVID-19 infection outcomes, older ages, by country

Country	Source	Date	Status Change	Sex	Age group		
					<50	50–69 (95%CI)	70+ (95%CI)
USA [35, 36]	CDC	16.05.2020	Infection to death	Both	Ref	9.8 (9.5–10.2)	53.1 (51.3–54.9)
Italy [37]	ISS	20.05.2020	Infection to death	Male	Ref	10.1 (8.9–11.4)	43.2 (38.2–49.0)
				Female	Ref	11.1 (9.0–13.7)	82.3 (67.4–100.4)
				Both	Ref	11.3 (10.1–12.6)	53.8 (48.5–59.8)
Spain [38]	ISCIII	21.05.2020	Infection to hospitalisation	Male	Ref	1.9 (1.8–1.9)	2.2 (2.2–2.3)
				Female	Ref	2.2 (2.1–2.2)	3.0 (2.9–3.0)
				Both	Ref	2.1 (2.1–2.1)	2.6 (2.6–2.7)
			Hospitalisation to death	Male	Ref	3.7 (3.2–4.2)	15.8 (13.7–18.1)
				Female	Ref	4.1 (0.6–28.6)	26.8 (3.8–187.9)
				Both	Ref	3.7 (3.3–4.1)	18.1 (16.2–20.2)
			Infection to death	Male	Ref	6.9 (6.0–8.0)	35.3 (30.7–40.6)
				Female	Ref	7.5 (6.1–9.1)	66.1 (54.8–79.8)
				Both	Ref	7.7 (6.9–8.7)	47.3 (42.3–52.8)
France [39]	Santé publique France	18.05.2020	Hospitalisation to death	Both	Ref	2.8 (2.6–3.1)	7.3 (6.7–7.9)
England [40]	Public Health England	20.05.2020	Infection to death	Male	Ref	7.2 (6.5–7.9)	19.0 (17.3–20.8)
				Female	Ref	9.3 (8.2–10.5)	36.2 (32.3–40.7)
				Both	Ref	8.7 (8.1–9.4)	26.6 (24.8–28.6)
Belgium [41]	Sciensano	26.05.2020	Infection to death	Male	Ref	5.0 (4.0–6.2)	20.2 (16.5–24.7)
				Female	Ref	9.1 (6.8–12.3)	51.7 (39.3–68.1)
				Both	Ref	7.4 (6.2–8.8)	32.0 (27.2–37.7)
Germany [42]	RKI	27.05.2020	Infection to death	Both	Ref	17.5 (14.2–21.6)	195.1 (159.6–238.5)

**Table 3:** Relative risk estimates for COVID-19 infection outcomes, sexes, by country (reference: male sex)

Country	Source	Date	State change	Age group	Relative risk (95%CI)
Italy	ISS	20.05.2020	Infection to death	<50	3.3 (2.6–4.2)
				50–69	3.0 (2.8–3.2)
				70+	1.7 (1.7–1.8)
Spain	ISCIII	21.05.2020	Infection to hospitalisation	<50	2.0 (1.9–2.0)
				50–69	1.7 (1.7–1.7)
				70+	1.5 (1.5–1.5)
			Hospitalisation to death	<50	1.8 (0.3–12.5)
				50–69	1.6 (1.5–1.7)
				70+	1.0 (1.0–1.1)
			Infection to death	<50	2.9 (2.3–2.7)
				50–69	2.7 (2.5–2.9)
				70+	1.6 (1.5–1.6)
England	Public Health England	20.05.2020	Infection to death	<50	2.6 (2.2–3.0)
				50–69	2.0 (1.9–2.1)
				70+	1.3 (1.3–1.4)
Belgium	Sciensano	20.05.2020	Infection to death	<50	4.2 (3.0–5.9)
				50–69	2.3 (2.0–2.7)
				70+	1.6 (1.6–1.7)

Regarding comorbidities (Table 4), the RR of diabetes ranged between 1.3 (95% CI 1.2–1.5) and 3.4 (3.4–3.5). In terms of cardiac and cardiovascular disease, the RR of death was 1.9 (1.7–2.0)−4.6 (4.6–4.7). For pulmonary and respiratory disease, the RR of death was 1.5 (1.3–1.7)−3.0 (3.0–3.0).

**Table 4:** Relative risks for COVID-19 deaths, by risk groups and country

Country	Source	Sample size	Date	Risk group	Outcome	Relative risk (95%CI)
Spain	ISCIII	250287	21.05.2020	Cardiovascular disease	Death	4.6 (4.6–4.7)
				Respiratory disease		3.0 (3.0–3.0)
				Diabetes		3.4 (3.4–3.5)
				At least one comorbidity		6.6 (6.5–6.6)
France	Santé publique France	3784	18.05.2020	Morbid obesity (BMI>40)	Death	0.9 (0.6–1.2)
				Diabetes		1.3 (1.2–1.5)
				Cardiac disease		1.9 (1.7–2.0)
				Pulmonary disease		1.5 (1.3–1.7)
				Immunodeficiency		1.6 (1.3–1.8)
				Renal disease		1.6 (1.4–1.9)
				Neuromuscular disorder		2.1 (1.8–2.4)

## Discussion

We believe that our meta-analyses add to existing evidence [17, 19–22] by assessing the magnitude of risk associated with comorbidities and other factors on the one hand and hospitalisation, ICU admission, and death on the other hand. Importantly, we take into account potential interactions with one main modifier, which is age. Our results have public health implications in four main fields:

First, we confirm that the risk of dying from COVID-19 is associated with the most prevalent existing comorbidities, such as cerebrovascular and cardiovascular diseases, hypertension, COPD, and renal disease, with RRs between two and three. Given the limited human and financial resources, knowing the exact magnitude of risk is important for effective protection and identification of the most vulnerable population groups. Explanations for the RR increase are in some instances related to the pathophysiology of the disease itself. For cardiovascular disease and hypertension, for example, a dysregulated innate immune response was found to influence severe COVID-19 infections [43]. In addition to that, it has been documented that the angiotensin-converting enzyme 2 (ACE2) has a vital role in the cardiovascular and immune systems, and it is involved in the heart function and the development of hypertension and diabetes mellitus [44]. Moreover, Zheng et al. considered that disease severity in patients with cardiovascular disease can be associated with increased secretion of ACE2 [45]. There is evidence that hypertensive patients may experience a decreased expression of ACE2, and consequently, an elevation of the angiotensin II levels that generates a severe manifestation of the disease [46]. The same protein and its poor regulation may also explain the link between COPD and smoking in terms of COVID-19 severity and mortality [22, 47]. Additionally, exacerbations in COPD cases are triggered by viral infections and environmental conditions [48]. For diabetes, the increased RR is explainable by reduced pulmonary function and a thickening of the pulmonary basal lamina [1, 49]. Factors like sex determine the COVID-19 risk and women might be protected by hormonal factors [8].

Second, we showed results of previous meta-regressions on the potential effects of age on COVID-19 outcomes and comorbidities [22]. For several comorbidities (cardiovascular disease, hypertension, and diabetes), we showed weaker associations with deaths among studies with a higher median age of patients. We want to highlight the effect modification of associations between comorbid conditions and severity of COVID-19 course as it is important for the NPIs just as for vaccination strategies. We considered that (i) adjusting for age will in most studies decrease associations and (ii) including adjusted estimates for our pooled estimates would, in most cases, leave age as the main predictor of severe course of the disease. However, this would disguise the considerable difference between those with predisposing conditions at young age and those healthy in young age.

Third, our analysis shows that the association of these comorbidities with hospitalisation and ICU admission is generally less strong for these same comorbidities and other risk factors and death. This corresponds to public data from Europe, where the proportion of infected people of older age or of other risk groups is relevantly higher in those who died compared to those who were hospitalised. In Spain, among infected aged 70 years and older, the risk of dying was higher than the risk of hospitalisation. In terms of comorbidities, while COVID-19 mortality risk among those with cardiovascular conditions is relevantly raised, the risk to be hospitalised or admitted to ICU is only moderately increased. Other studies have also found that for patients with cerebrovascular disease and with diabetes the risk increase of ICU admission was lower than the risk of dying compared to other population groups [19, 50]. This finding is important, as it implies that public health measures to protect healthcare surge capacities should not be equalled with measures to protect the vulnerable population from death in the fight against COVID-19. Even if—hypothetically—protecting all people at high risk of death, the effect on healthcare surge capacities, measured by hospital beds, critical care beds, healthcare workers, and healthcare expenditure [51], will not be equally effective. Interestingly, our meta-analyses showed less risk of severe outcomes of COVID-19 for healthcare professionals. This might be explained by a lower likelihood for underreporting in this population group but also by the healthy worker effect [52]. Therefore, comparative studies on this occupational group are needed.

The limitations of our work derived from the restricted and rapid search in one main database of medical literature. As we did not include articles in the Chinese language in our search due to lack of interpreters, that could have affected our findings. The nature of the included data was often based on hospital recording implying bias in a sense that more severely symptomatic patients are more likely included. Although our assessment revealed high to moderate study quality, studies based on hospital records are highly selective regarding the population included.

The limitation of the results from publicly available data derived from the limited and varying data sources, which restrict generalisability and country comparisons. Also, our effect estimates for some countries only investigate subgroups of hospitalised cases (e.g., France), which may lead to a systematic underestimation of the effect estimates, since these individuals likely have an increased risk of severity. However, the qualitative implications apply across different geographic regions as well as different data sources.

In conclusion, we found estimates in the range of two and three indicating that populations with comorbidities, such as cerebrovascular and cardiovascular diseases, hypertension, COPD, and renal disease are most at risk of dying from COVID-19. We also provided evidence synthesis for the effects of comorbidities on the severity of COVID-19 and effect modification of these effects by age in order to target public health measures towards groups at risk. The detailed analyses on effect modification revealed that effects of comorbidities on disease severity increase for several comorbidities with young age, which has important implications for the planning of vaccine strategies as well as non-pharmacological interventions targeting risk groups.

## Supplementary Information

**Additional file 1: **Published Systematic Reviews as obtained from PubMed search. **Table 1.** Published Systematic Reviews as obtained from PubMed search. Characteristics of the 73 included studies. **Table 2.** Characteristics of the 73 included studies. **Table 3.** Severe disease definition used by the included studies. Results of the risk of bias assessment. **Table 4.** Results of the risk of bias assessment. Meta-analysis by individual factor and outcome: Comorbidities: Asthma, Cancer, Cardiovascular disease, Cerebrovascular disease, COPD, Chronic Renal Failure, Diabetes, Diseases of liver, Diseases of the digestive system, Endocrine diseases, Hypertension, Immunocompromised condition, Mycobacterial diseases, Other diseases or unspecified, Other respiratory diseases. Demographic, Occupational or lifestyle Factors: Healthcare workers, Male, Obesity, Smoking. Meta-analysis of comorbidities and severe clinical course of disease. Meta-analysis of Comorbidities and ICU admission. Meta-analysis of Comorbidities and Death. Meta-analysis of Epidemiologic Factors and severe clinical course of disease. Meta-analysis of Epidemiologic Factors and ICU admission. Meta-analysis of Epidemiologic Factors and death. References

## Data Availability

The datasets used and/or analysed during the current study are available from the corresponding author on reasonable request.

## Abbreviations

COVID-19: Coronavirus disease
ICU: Intensive care unit
NPI: Non-pharmacological interventions
ECMO: Extracorporeal membrane oxygenation
ICD-11: International Classification of Disease 11th Revision
ORs: Odds ratios
HRs: Hazard ratios
RRs: Relative risks
USA: United States of America
CI: Confidence intervals
AICc: Akaike’s information criterion
BIC: Bayesian information criterion
COPD: Chronic obstructive pulmonary disease
