# Supplementary material for: Effect modification of the association between comorbidities and severe course of COVID-19 disease by age of study participants: a systematic review and meta-analysis
Source: Syst Rev. 2021 Jun 30;10:194. doi: 10.1186/s13643-021-01732-3 (PMC8244460; doi:10.1186/s13643-021-01732-3)
Supplement: Supplementary file 1 — Additional file 1: Published Systematic Reviews as obtained from PubMed search. Table 1. Published Systematic Reviews as obtained from PubMed search. Characteristics of the 73 included studies. Table 2. Characteristics of the 73 included studies. Table 3. Severe disease definition used by the included studies. Results of the risk of bias assessment. Table 4. Results of the risk of bias assessment. Meta-analysis by individual factor and outcome: Comorbidities: Asthma, Cancer, Cardiovascular disease, Cerebrovascular disease, COPD, Chronic Renal Failure, Diabetes, Diseases of liver, Diseases of the digestive system, Endocrine diseases, Hypertension, Immunocompromised condition, Mycobacterial diseases, Other diseases or unspecified, Other respiratory diseases. Demographic, Occupational or lifestyle Factors: Healthcare workers, Male, Obesity, Smoking. Meta-analysis of comorbidities and severe clinical course of disease. Meta-analysis of Comorbidities and ICU admission. Meta-analysis of Comorbidities and Death. Meta-analysis of Epidemiologic Factors and severe clinical course of disease. Meta-analysis of Epidemiologic Factors and ICU admission. Meta-analysis of Epidemiologic Factors and death. References [file 13643_2021_1732_MOESM1_ESM.docx]

Supplement

Index

[1. Published Systematic Reviews as obtained from PubMed search 2](#_Toc56419326)

[**Table 1.** Published Systematic Reviews as obtained from PubMed search 2](#_Toc56419327)

[2. Characteristics of the 73 included studies 7](#_Toc56419328)

[**Table 2.** Characteristics of the 73 included studies 7](#_Toc56419329)

[**Table 3.** Severe disease definition used by the included studies 22](#_Toc56419330)

[3. Results of the risk of bias assessment 34](#_Toc56419331)

[**Table 4.** Results of the risk of bias assessment 34](#_Toc56419332)

[4. Meta-analysis by individual factor and outcome 39](#_Toc56419333)

[Comorbidities 39](#_Toc56419334)

[Demographic, Occupational or lifestyle Factors 66](#_Toc56419335)

[5. Meta-analysis of comorbidities and severe clinical course of disease 74](#_Toc56419336)

[6. Meta-analysis of Comorbidities and ICU admission 79](#_Toc56419337)

[7. Meta-analysis of Comorbidities and Death 81](#_Toc56419338)

[8. Meta-analysis of Epidemiologic Factors and severe clinical course of disease 86](#_Toc56419339)

[9. Meta-analysis of Epidemiologic Factors and ICU admission 88](#_Toc56419340)

[10. Meta-analysis of Epidemiologic Factors and death 89](#_Toc56419341)

[11. References 90](#_Toc56419342)

## Published Systematic Reviews as obtained from PubMed search

**Table 1.** Published Systematic Reviews as obtained from PubMed search

| **First Author** | **Topic** | **Date published** | **Journal** | **Population** | **Exposure** | **Comparison** | **Outcome** | **Number of studies included** | **Value added by our review** |
| --- | --- | --- | --- | --- | --- | --- | --- | --- | --- |
| **Several exposures** | | | | | | | | | |
| Couper, K. et al (1). | Association between key resuscitation interventions (chest compressions, defibrillation, CPR) and aerosol generation and airborne transmission of infection | Apr 20, 2020 | Resuscitation | Rescuers (healthcare worker or lay person) delivery chest compressions &/or defibrillation &/or CPR to a person suffering a cardiac arrest | COVID-19 | Chest compressions and/or defibrilla- tion and/or CPR, with or without PPE (personal protective equipment) | Aerosol generation, or transmission of infection. | 11 | Inclusion of other comorbidities and interaction with age |
| Hu, Y. et al (2). | Risk factors of COVID-19 | Apr 14, 2020 | J Clin Virol | Patients diagnosed with COVID-19 | Patient characteristics (gender, age, comorbidities, etc.) | Comparison of different outcomes | Symptoms, ARDS, ACI (acute cardiac injury), AKI (acute kidney injury), shock, incidence of severity and death | 21 | Inclusion of other comorbidities and interaction with age |
| Nasiri, M. et al (3). | Better understanding of clinical, laboratory, epidemiologic and mortality findings of COVID-19 | July 21, 2020 | Frontiers in Medicine | Patients diagnosed with COVID-19 by RT-PCR | Clinical manifestations, comorbidities, laboratory findings and radiologic findings | Comparison of different outcomes & their associated risk factors | Hospitalisation, severe disease, death, ICU admission, mean duration between time of hospitalisation and death | 32 | Study of different comorbidities and conditions for different outcomes |
| Xu, L. et al (4). | Risk factors for 2019 novel coronavirus disease (COVID-19) patients progressing to critical illness: a systematic review and meta-analysis | Jun 23, 2020 | Aging | Patients diagnosed with COVID-19 | Elderly age, male, high body mass index, high breathing rate, and underlying diseases | Severe and non-severe patients | Severe disease (defined as 1)Respiratory distress with respiratory frequency 30/min; 2) Pulse Oximeter Oxygen Saturation 93% at rest; 3) Oxygenation index (artery partial pressure of oxygen/inspired oxygen fraction, PaO2/FiO2) 300 mmHg (1 mmHg=0.133 kPa)) | 20 | Inclusion of deaths and ICU admission as outcomes, calculation of RR based on crude numbers, interaction with age |
| Zhao, Q. et al (5). | The risk of severe Covid-19 in patients with pre-existing chronic obstructive pulmonary disease (COPD) and ongoing smoking history. | Apr 15, 2020 | J Med Virol | Patients diagnosed with COVID-19 | Pre-existing COPD and ongoing smoking history | Severe and non-severe cases | Severe COVID-19 & death | 11 | Inclusion of other comorbidities and interaction with age |
| Zheng, Z. et al (6). | Risk factors for the progression of COVID-19 | Apr 23, 2020 | J Infect | Patients diagnosed with COVID-19 | Sociodemographic (sex, age), behavioural (smoking) and clinical risk factors (comorbidities, clinical manifestations, laboratory examinations) | Critical/mortal cases vs non-critical cases | Disease progression: critical case & death | 13 | No limit on recruited participants, calculation of RR based on crude numbers, interaction with age |
| **Arterial hypertension** | | | | | | | | | |
| Zuin, M. et al (7). | Risk of death in COVID-19 infection patients with and without arterial hypertension | Apr 11, 2020 | J Infect | Patients diagnosed with COVID-19 | Arterial hypertension (HT) | Patients with and without HT | Prevalence of HT, death | 3 | Inclusion of other comorbidities |
| **Cardiovascular** | | | | | | | | | |
| Li, B. et al (8). | Association of cardiovascular metabolic diseases with the development of COVID-19 | Mar 11, 2020 | Clinical Research in Cardiology | Patients diagnosed with COVID-19 | Cardiovascular metabolic diseases | Patients with Cardiovascular metabolic diseases compared to other comorbidities | ICU/severe and non-ICU/severe | 6 | Inclusion of other comorbidities and interaction with age |
| Matsushita, K. et al. (9) | The Relationship of COVID-19 Severity with Cardiovascular Disease and Its Traditional Risk Factors: A Systematic Review and Meta-Analysis | Sep 22, 2020 | Glob Heart. | Adult patients (>18 years) diagnosed with COVID-19 | Cardiovascular Disease | Different Clinical (hypertension, DM, prior CVD) and sociodemographic (age, sex & smoking) risk factors | COVID-19 severity (death, ARDS, mechanical ventilation, ICU admission) | 21 | Inclusion of other comorbidities and interaction with age |
| **Stroke** | | | | | | | | | |
| Aggarwal, G. et al (10). | Association between stroke and severity of disease or mortality | Apr 20, 2020 | Int J Stroke | COVID-19 patients with Cerebrovascular disease | Stroke (cerebrovascular disease) | Cerebrovascular disease in COVID-19 patients with or without severe disease and in non-survivors versus survivors | Disease severity (Severe disease, death, recovery) | 7 | Inclusion of other comorbidities and interaction with age |
| Li, J. W. et al (11). | Severity differences in acute cardiac injury and acute cardiac injury with mortality. | Apr 16, 2020 | Prog Cardiovasc Dis | Cardiac Injury Patients | COVID-19 | Those admitted to ICU and not admitted, non-survivors and survivors, severe vs non-severe cases | Infection severity on cardiac injury, death | 22 | Inclusion of other comorbidities and interaction with age |
| Santoso, A. et al (12). | Association between cardiac injury and mortality, the need for intensive care unit (ICU) care, acute respiratory distress syndrome (ARDS), and severe coronavirus disease 2019 | Apr 19, 2020 | Am J Emerg Med | Patients diagnosed with COVID-19 | Cardiac injury (highly sensitive cardiac troponin I (hs-cTnl) N99th percentile) | Comparison of the association of cardiac injury with different outcomes | Primary outcome was mortality, and the secondary outcomes were ARDS, the need for ICU care, and severe COVID-19. | 13 | Inclusion of other comorbidities and interaction with age |
| **Diabetes** | | | | | | | | | |
| Huang, I. et al (13). | Association between DM and poor outcome in patients with COVID-19 pneumonia | Apr 17, 2020 | Diabetes Metab Syndr | COVID-19 patients | DM | Patients with and without DM | Composite poor outcome, including mortality, severe COVID-19, acute respiratory distress syndrome (ARDS), need for intensive care unit (ICU) care, and disease progression. | 30 | Inclusion of other comorbidities and interaction with age |

## Characteristics of the 73 included studies

**Table 2.** Characteristics of the 73 included studies

| **First Author*** | **Country** | **Sample size** | **Study design** | **Characteristics of population** | **Underlying data** | **Study time-frame** | **Age-range, Median (IQR), or Median (SD) in years** | **Reported outcome** | **Outcome definition** |
| --- | --- | --- | --- | --- | --- | --- | --- | --- | --- |
| Cai, Q. et al. (14) | China | 298 | Case series | Confirmed COVID-19 pneumonia | Medical/Clinical records | 11 January to 06 February 2020 | Median (IQR): 47 (33-61) | Disease severity | Based on the official clinical practice guideline of the American Thoracic Society and Infectious Disease Society of America |
| Cao, J. et al. (15) | China | 102 | Retrospective case series | Laboratory-confirmed COVID-19 cases | Medical/Clinical records | 03 January to 15 February 2020 | Median (IQR): 54 (37-67) | ICU admission |  |
| CDC COVID-19 Response Team (16) | USA | 7162 | Descriptive study | Laboratory-confirmed COVID-19 cases | Official reported data | 12 February to 28 March 2020 | all ages | Hospitalisation |  |
| Chen, G. et al. (17) | China | 21 | Retrospective study | Patients with COVID-19 | Medical/Clinical records | Late December 2019 to 27 January 2020 | Median (IQR): 56 (50 – 65) | Disease severity | Based on "Sixth revised trial Version of the Novel Coronavirus Pneumonia Diagnosis and Treatment Guidance" |
| Chen, Q. et al. (18) | China | 145 | Single centre retrospective observational study | Patients with confirmed Corona Virus Disease 2019 | Medical/Clinical records | 01 January to 11 March 2020 | Mean (SD): 47.5 (14.6) | Disease severity | Diagnosis and Treatment of Pneumonia caused by SARSCoV-2 (version 7) issued by of National Health Commission of the People’s Republic of China. |
| Chen, T. et al. (19) | China | 799** | Case series study | Hospitalised/clinically admitted COVID-19 cases | Medical/Clinical records | 13 January to 28 February 2020 | Median (IQR): 62 (44-70) | Death |  |
| Cheng, Y. et al. (20) | China | 701 | Prospective cohort study | Hospitalised COVID-19 cases | Medical/Clinical records | 28 January to 11 February 2020 | Median (IQR): 63 (50-71) | AKI and in-hospital death | Based on "Fifth revised trial Version of the Novel Coronavirus Pneumonia Diagnosis and Treatment Guidance" |
| Deng, G. et al. (21) | China | 44672 | Meta-analysis | Laboratory confirmed cases | Official reported data | Up to 11 February 2020 | - | Death |  |
| Deng, Q. et al. (22) | China | 112 | Case series | Confirmed COVID-19 pneumonia | Medical/Clinical records | 06 January to 20 February 2020 | Median (IQR): 65.0 (49.0-70.8) | Disease severity and composite endpoint: admission to intensive care unit (ICU), or mechanical ventilation, or extracorporeal membrane oxygenation (ECMO), or death | Interim Guidance for Novel Coronavirus Pneumonia (Trial Implementation of Sixth Edition) |
| Du, R. et al. (23) | China | 109 | Multi-centre observational study | Hospitalised COVID-19 cases | Medical/Clinical records | 25 December 2019 to 24 February 2020 | Mean (SD):70.7 (10.9)  Range 43-99 | ICU admission |  |
| Duanmu, Y. et al. (24) | USA | 100 | Observational, cross‐sectional study | Emergency department patients with a laboratory‐confirmed diagnosis of COVID‐19 | Medical/Clinical records | 04 March to 23 March 2020 | Median: 45 | Admission at emergency department |  |
| Fan, J. et al. (25) | China | 21 | Retrospective longitudinal study | Surviving and non-surviving COVID-19 cases | Medical/Clinical records | 18 January to 15 March 2020 | Mean (SD): 62.5 (12.6) | Death |  |
| Feng, Y. et al. (26) | China | 476 | Multi-centre retrospective study | Patients with diagnosis of COVID-19 | Medical/Clinical records | 01 January to 21 March 2020 | Median (IQR): 53 (40-64) | Disease severity | Fifth version of the guidelines issued by the National Health Commission of China on Diagnosis and Treatment of COVID-19 |
| Goyal, P. et al. (27) | USA | 393 | Retrospective case series | Adults with confirmed Covid-19 | Medical/Clinical records | 03 March to 27 March 2020 | Median (IQR): 62.2 (48.6-73.7) | Invasive mechanical ventilation |  |
| Guan, W. et al. (28) | China | 1590 | Retrospective case study | Patients with COVID-19 | Medical/Clinical records | 21 November 2019 to 31 January 2020 | Mean (SD): 48.9 (16.3) | Admission to an intensive care unit, the use of mechanical ventilation, or death | Based on 2007 American Thoracic Society/Infectious Disease Society of America guidelines. |
| Guan, Wei-jie. et al. (29) | China | 1099 | Descriptive study | Laboratory-confirmed COVID-19 cases | Medical/Clinical records | 11 December 2019 to 31 January 2020 | Median (IQR): 47 (35-58) | Admission to intensive care unit, invasive ventilation, or death |  |
| Guo, T. et al. (30) | China | 187 | Retrospective single-centre case series | Hospitalised COVID-19 cases | Medical/Clinical records | 23 January to 23 February 2020 | Mean (SD): 58.50 (14.66) | Death |  |
| Huang, C. et al. (31) | China | 41 | Single-centre cohort Study | Hospitalised COVID-19 cases | Medical/Clinical records | 16 December 2019 to 22 January 2020 | Median (IQR): 49 (41–58) | ICU admission |  |
| Ji, D. et al. (32) | China | 208 | Retrospective, non-interventional study | Patients with confirmed COVID-19 | Medical/Clinical records | 20 January to 18 March 2020 | Mean (SD): 44.0 (16.3) | Disease progression |  |
| Ji, Dong. et al. (33) | China | 202 | Retrospective study | Admitted patients with confirmed COVID-19 | Medical/Clinical records | 20 January to 17 February 2020 | Median (IQR): 44.5 (34.8-54.1) | Disease progression |  |
| Kalligeros, M. et al.(34) | USA | 103 | Retrospective cohort study | Patients Hospitalised with COVID-19 | Medical/Clinical records | 17 February to 05 April 2020 | Median (IQR): 60 (52-70) | ICU admission |  |
| Lei, S. et al. (35) | China | 34 | Multi-centre, retrospective study | Patients with elective surgeries | Medical/Clinical records | 01 January to 05 February 2020 | Median (IQR): 55 (43-63) | ICU admission |  |
| Li, K. et al. (36) | China | 83 | Retrospective study | Patients with COVID-19 pneumonia | Medical/Clinical records | January to February 2020 | Mean (SD): 45.5 (12.3) | Disease severity | Diagnosis and Treatment of Novel Coronavirus Pneumonia (Fifth Trial Version) of China |
| Li, X. et al. (37) | China | 548 | Retrospective study | Patients with COVID-19 | Medical/Clinical records | 26 January to 03 March 2020 | Median (IQR): 60 (48-69) | Disease severity | Based on the official clinical practice guideline of the American Thoracic Society and Infectious Disease Society of America. |
| Li, Y. et al. (38) | China | 25 | Retrospective single-centre Study | Hospitalised COVID-19 cases and Healthcare staff | Medical/Clinical records | 01 January to 03 March 2020 | Median: 51  Range: 22-69 | Disease severity and vital status | Based on "Seventh revised trial Version of the Novel Coronavirus Pneumonia Diagnosis and Treatment Guidance" |
| Liang, W. et al. (39) | China | 1590 | Prospective cohort study | Hospitalised laboratory-confirmed COVID-19 cases | Medical/Clinical records | <31 January 2020 | - | Severe events | Intensive care unit requiring invasive ventilation, or death |
| Liu, Jing. et al. (40) | China | 40 | Retrospective single-centre study | Confirmed COVID-19 pneumonia | Medical/Clinical records | 05 January to 24 January 2020 | Mean (SD): 48.7 (13.9) | Disease severity | Based on "Fifth revised trial Version of the Novel Coronavirus Pneumonia Diagnosis and Treatment Guidance" |
| Liu, Jingyuan. et al. (41) | China | 61 | Prospective study | Confirmed COVID-19 pneumonia | Medical/Clinical records | 13 January to 31 January 2020 | Median: 40  Range: 01-86 | Severe illness | Based on "Fourth revised trial Version of the Novel Coronavirus Pneumonia Diagnosis and Treatment Guidance" |
| Liu, W. et al. (42) | China | 78 | Retrospective study | Diagnosed patients with COVID-19 associated pneumonia | Medical/Clinical records | 30 December 2019 to 15 January 2020 | Median (Q1,Q3): 38 (33,57) | Disease progression | Based on “Fourth revised trial version of the Diagnosis and Treatment Protocol for Novel Coronavirus Infection-Induced Pneumonia” |
| Liu, Y. et al. (43) | China | 109 | Retrospective study | Confirmed COVID-19 pneumonia | Medical/Clinical records | 02 January to 12 February 2020 | Mean (IQR): 55 (43-66)  Range 22-94 | Acute respiratory distress syndrome (ARDS) | Based in The Berlin definition to determine the presence and severity of ARDS (Acute Respiratory Distress Syndrome) |
| Ma, J. et al. (44) | China | 37 | Single-centre, retrospective study | Cancer patients with COVID-19 infection | Medical/Clinical records | 01 January to 30 March 2020 | Median (IQR): 62 (59-70) | Disease severity | National Health Commission of China. New coronavirus pneumonia prevention and control program (7th edition) |
| Mao, L. et al. (45) | China | 214 | Retrospective, observational case series | Hospitalised patients with laboratory-confirmed diagnosis of severe acute respiratory syndrome coronavirus 2 infection | Medical/Clinical records | 16 January to 19 February 2020 | Mean (SD): 52.7 (15.5) | Disease severity | Based on the official clinical practice guideline of the American Thoracic Society and Infectious Disease Society of America. |
| McMichael, T. et al. (46) | USA | 167 | Case investigations | Confirmed cases of Covid-19 | Official reported data | 20 January to 18 March 2020 | Median: 72  Range: 21-100 | Hospitalisation |  |
| Nikpouraghdam, M. et al. (47) | Iran | 2964 | Retrospective study | COVID-19 patients | Medical/Clinical records | 19 February to 15 April 2020 | Mean (SD): 55.50 (15.15) | Death |  |
| Qin, C. et al. (48) | China | 452 | Retrospective study | Patients with COVID-19 | Medical/Clinical records | 10 January to 12 February 2020 | Median (IQR): 58 (47-67)  Range: 22-95 | Disease severity | Based on "Fifth revised trial Version of the Novel Coronavirus Pneumonia Diagnosis and Treatment Guidance" |
| Ruan, Q. et al. (49) | China | 150 | Retrospective multi-centre study | Laboratory-confirmed COVID-19 cases | Medical/Clinical records | - | - | Death |  |
| Shi, Y. et al. (50) | China | 487 | Retrospective cohort | Patient with COVID-19 | Medical/Clinical records | January to 17 February 2020 | Mean (SD): 46 (19) | Disease severity | No definition |
| Simonnet, A. et al. (51) | France | 124 | Retrospective cohort study | Patients admitted in intensive care for SARS-CoV-2 | Medical/Clinical records | 27 February to 06 April 2020 | Median (IQR): 60 (51-70) | ICU admission |  |
| Sun, S. et al. (52) | China | 116 | Retrospective study | Confirmed cases with COVID-19 | Medical/Clinical records | 19 January to 20 February 2020 | Median (IQR): 47.0 (37.0–54.5) | Disease severity | Based on “Seventh revised version of the Novel Coronavirus Pneumonia Diagnosis and Treatment Interim Guidance” |
| Tang, N. et al. (53) | China | 183 | Prospective study | Confirmed COVID-19 pneumonia | Medical/Clinical records | 01 January to 13 February 2020 | Mean (SD): 54.1 (16.2)  Range: 14-94 | Death |  |
| The Novel Coronavirus Pneumonia Emergency Response Epidemiology Team (54) | China | 44672 | Descriptive and exploratory study | Confirmed COVID-19 pneumonia | Official reported data | 31 December 2019 to 11 February 2020 | all ages | Death |  |
| Tian, S. et al. (55) | China | 262 | Retrospective study | Hospitalised COVID-19 cases | Medical/Clinical records | 20 January to 10 February 2020 | Median: 47.5  Range: 1-94 | Disease severity | No definition |
| Tomlins, J. et al. (56) | UK | 95 | Retrospective single-centre study | Patients hospitalised with SARS-COV-2 infection | Medical/Clinical records | 10 March to 06 April 2020 | Median (IQR): 75 (59-82) | Death |  |
| Wan, S. et al. (57) | China | 135 | Single-centre cohort Study | Hospitalised COVID-19 cases | Medical/Clinical records | 23 January to 08 February 2020 | Median (IQR): 47 (36‐55) | Disease severity | Based on "Fifth revised trial Version of the Novel Coronavirus Pneumonia Diagnosis and Treatment Guidance" |
| Wang, Dawei et al. (58) | China | 107 | Retrospective case series | Discharged patients with COVID-19 | Medical/Clinical records | Up to 10 February 2020 | Median (IQR): 51 (36-65) | Death |  |
| Wang, D. et al. (59) | China | 138 | Retrospective, single-centre case series | Hospitalised COVID-19 cases | Medical/Clinical records | 01 January to 03 February 2020 | Median (IQR): 56 (42-68)  Range 22-92 | ICU admission |  |
| Wang, L. et al. (60) | China | 339 | Retrospective, single-centre study | Hospitalised COVID-19 cases | Medical/Clinical records | 01 January to 05 March 2020 | Median (IQR): 69 (65-76) | Death |  |
| Wang, R. et al. (61) | China | 125 | Descriptive study | Patients infected SARS-CoV-2 | Medical/Clinical records | 20 January to 18 February 2020 | Mean (SD): 38.76 (13.79) | Critically ill patients | Based in The Berlin definition to determine the presence and severity of ARDS (Acute Respiratory Distress Syndrome). |
| Wang, X. et al. (62) | China | 1012 | Case series | Laboratory-confirmed COVID-19 cases | Medical/Clinical records | 7 February to 22 February 2020 | Median (IQR): 50 (39-58)  Range 16-89 | Aggravation of illness |  |
| Wang, Y. et al. (63) | China | 344 | Case series | Hospitalised COVID-19 cases | Medical/Clinical records | 25 January to 25 February 2020 | Median (IQR): 64 (52-72) | Death |  |
| Wei, X. et al. (64) | China | 252 | Retrospective study | COVID-19 patients | Medical/Clinical records | 13 February to 03 March 2020 | Mean (SD): 64.8 (13.3) | Disease severity | Chinese Centre for Disease Control (CDC) guidelines and a previous literature. |
| Wei, YY. et al. (65) | China | 167 | Observational study | Hospitalised confirmed COVID-19 patients | - | - | Mean (SD): 42.31 (15.29) | Disease severity | No definition |
| Williamson, E. et al. (66)*** | England | 17425445 | Cohort study | Electronic health record data linked to data on COVID-19-related deaths | Official reported data | 01 February to 25 April 2020 | - | Death |  |
| Wu, C. et al. (67) | China | 201 | Retrospective cohort study | Confirmed COVID-19 pneumonia | Medical/Clinical records | 25 December 2019 to 13 February 2020 | Median (IQR): 51 (43-60)  Range: 21-83 | Development of Acute respiratory distress syndrome (ARDS) and death | World Health Organization interim guidance to define ARDS. |
| Xie, H. et al. (68) | China | 79 | Retrospective study | Laboratory-confirmed COVID-19 cases | Medical/Clinical records | 02 February to 23 February 2020 | Median: 60  Range: 27-87 | Disease severity | Based on "Sixth revised trial Version of the Novel Coronavirus Pneumonia Diagnosis and Treatment Guidance" |
| Xu, Y. (69) | China | 50 | Retrospective study | Laboratory-confirmed COVID-19 cases | Medical/Clinical records | January to February 2020 | Mean (SD): 43.9 (16.8)  Range: 3-85 | Disease severity | Based on "Fifth revised trial Version of the Novel Coronavirus Pneumonia Diagnosis and Treatment Guidance" |
| Yan, Y. et al. (70) | China | 193 | Retrospective study | Patients confirmed with COVID-19 pneumonia | Medical/Clinical records | 10 January to 24 February 2020 | Median (IQR): 64 (49-73) | ICU admission and death |  |
| Yang, X. et al. (71) | China | 52 | Single-centre, retrospective, observational study | Patients with severe covid-19 | Medical/Clinical records | 24 December 2019 to 09 February 2020 | Mean (SD): 59.7 (13.3) | Death |  |
| Yao, Q. et al. (72) | China | 108 | Single-centre, retrospective, observational study | Confirmed COVID-19 pneumonia | Medical/Clinical records | 30 January to 11 February 2020 | Median (IQR): 52 (37-58) | Disease severity and death | Based on the official clinical practice guideline of the American Thoracic Society and Infectious Disease Society of America. |
| Yu, X. et al. (73) | China | 92 | Retrospective cohort study | Patients diagnosed with COVID‑19 | Medical/Clinical records | 19 January to 19 March 2020 | Mean (SD): 55 (16) | Severe at admission | No definition |
| Yuan, M. et al. (74) | China | 27 | Retrospective study | Patients with confirmed COVID-19 | Medical/Clinical records | 1 January 2020 to 25 January 2020 | Median (IQR): 60 (47-69) | Death |  |
| Zhang, G. et al. (75) | China | 221 | Retrospective study | Patients with 2019 novel coronavirus (2019-nCoV) infected pneumonia | Medical/Clinical records | 02 January to 10 February 2020 | Median (IQR): 55.0 (39.0-66.5) | Disease severity | Based on the official clinical practice guideline of the American Thoracic Society and Infectious Disease Society of America. |
| Zhang, J. et al. (76) | China | 19 | Single-centre, retrospective case series study | Patients with laboratory confirmed SARS-CoV-2 pneumonia | Medical/Clinical records | 16 January to 20 February 2020 | Median: 73 | Disease severity | World Health Organization (WHO) interim guidance |
| Zhang, Jixiang. et al. (77) | China | 663 | Retrospective cohort study | Patients diagnosed with COVID-19 | Medical/Clinical records | 11 January to 06 February 2020 | Median (IQR): 55.6 (44-69) | Disease severity and death | Interim guidelines from the World Health Organization and the National Health Commission of China |
| Zhang, Jin-jin. et al. (78) | China | 140 | Observational study | Hospitalised Covid-19 cases | Medical/Clinical records | 16 January to 03 February 2020 | Median: 57  Range: 25-87 | Disease severity | Based on "Diagnosis and Treatment Guideline for SARS-CoV-2" issued by the Chinese National Health Committee (version 3-5). |
| Zhang, Y. et al. (79) | China | 80 | Cohort study | Hospitalised patients with laboratory confirmed COVID/19 | Medical/Clinical records | January to February 2020 | Median (IQR): 49.5 (37.75-63.5) | Disease severity | Based on "Fifth revised trial Version of the Novel Coronavirus Pneumonia Diagnosis and Treatment Guidance" |
| Zhao, X. et al. (80) | China | 91 | Retrospective study | Patients diagnosed with COVID-19 | Medical/Clinical records | 16 January to 10 February 2020 | Median: 46 | Disease severity | No definition |
| Zheng, KI. et al. (81) | China | 66 | Multi-centre study | Adult patients with laboratory-confirmed COVID-19 | Medical/Clinical records | 01 January to 29 February 2020 | Mean: 47 | Disease severity | Based on "Seventh revised trial Version of the Novel Coronavirus Pneumonia Diagnosis and Treatment Guidance" |
| Zheng, S. et al. (82) | China | 96 | Retrospective cohort study | Patients with laboratory confirmed SARS-CoV-2 infection | Medical/Clinical records | 19 January to 20 March 2020 | Median (IQR): 55 (44.3-64.8) | Disease severity | Based on “Sixth edition of the Guideline for Diagnosis and Treatment of SARS-CoV-2” |
| Zheng, F. et al. (83) | China | 161 | Retrospective study | Admitted confirmed cases of COVID-19 | Medical/Clinical records | 17 January to 07 February 2020 | Median (IQR): 45 (33.5-57) | Disease severity | Based on "Fifth revised trial Version of the Novel Coronavirus Pneumonia Diagnosis and Treatment Guidance" |
| Zhou, F. et al. (84) | China | 191 | Retrospective, multi-centre cohort study | Hospitalised Covid-19 cases | Medical/Clinical records | 29 December 2019 to 31 January 2020 | Median (IQR): 56 (46-67)  Range: 18-87 | Death |  |
| Zhu, Z. et al. (85) | China | 127 | Retrospective study | Hospitalised patients with confirmed COVID-19 | Medical/Clinical records | 23 January to 20 February 2020 | Mean (SD): 50.90 (15.26) | Disease severity | Based on “Sixth edition of the Guideline for Diagnosis and Treatment of SARS-CoV-2” |
| Zou, Y. et al. (86) | China | 303 | Retrospective study | Patients diagnosed with COVID-19 | Medical/Clinical records | 20 January to 24 February 2020 | Median: 51 | Disease severity | Based on "Seventh revised trial Version of the Novel Coronavirus Pneumonia Diagnosis and Treatment Guidance”. |
| *All the studies were published in 2020  ** Analysis based on 274 patients  ***National primary care electronic health record data linked to in-hospital COVID-19 death data | | | | | | | |  |  |

**Table 3.** Severe disease definition used by the included studies

| **First Author** | **Guideline document** | **Definition** |
| --- | --- | --- |
| Cai, Q. et al. | Based on the official clinical practice guideline of the American Thoracic Society and Infectious Disease Society of America (87). | Validated definition includes either one major criterion or three or more minor criteria  Minor criteria   - Respiratory rate ≥ 30 breaths/min - PaO2/FiO2 ratio ≤ 250 - Multilobar infiltrates - Confusion/disorientation - Uremia (blood urea nitrogen level ≥ 20 mg/dl) - Leukopenia* (white blood cell count < 4,000 cells/μl) - Thrombocytopenia (platelet count < 100,000/μl) - Hypothermia (core temperature < 36°C) - Hypotension requiring aggressive fluid resuscitation   Major criteria   - Septic shock with need for vasopressors - Respiratory failure requiring mechanical ventilation |
| Chen, G. et al. | Based on "Sixth revised trial Version of the Novel Coronavirus Pneumonia Diagnosis and Treatment Guidance" | 1. Mild cases: The clinical symptoms are mild and no pneumonia manifestation can be found in imaging.  2. Moderate cases: Patients have symptoms like fever and respiratory tract symptoms, etc., and pneumonia manifestation can be seen in imaging.  3. Severe cases: Meeting any of the following:   - respiratory distress, - respiratory rate ≥ 30 breaths/min; - SpO2 ≤ 93% at rest; and - PaO2/FIO2 ≤ 300. - Patients with greater than 50% lesion progression within 24 to 48 hours in pulmonary imaging should be treated as severe cases.   4. Critically ill cases: Meeting any of the following:   - respiratory failure occurs and mechanical ventilation is required, - shock, and - complications from other organ failure that require monitoring and treatment in the ICU. |
| Chen, Q. et al. | Diagnosis and Treatment of Pneumonia caused by SARSCoV-2 (version 7) issued by of National Health Commission of the People’s Republic of China. | Severe cases were defined as follow:   - The respiratory rate is more than 30 times/min; - In the resting state, transcutaneous oxygen saturation (SaO2) ≤ 93%; - (3) Oxygenation index (PaO2/FiO2) ≤ 300 mmHg. Sepsis was defined according to the new definition of Sepsis-3. |
| Cheng, Y. et al. | Based on "Fifth revised trial Version of the Novel Coronavirus Pneumonia Diagnosis and Treatment Guidance" | 1. Severe cases were defined as follow:   - respiratory rate > 30 breaths/min, - oxygen saturation  ≤ 93%, or - PaO2/FiO2 ratio  ≤ 300 mm Hg.   2. Critical severe cases were defined as including 1 of the following criteria:   - shock; - respiratory failure requiring mechanical ventilation; - combination with other organ failures; and admission to intensive care unit. |
| Deng, Q. et al. | Interim Guidance for Novel Coronavirus Pneumonia (Trial Implementation of Sixth Edition) | 1. Severe cases should meet one of the three criteria:   - respiratory distress and respiratory rate higher than 30 times per minute; - fingertip blood oxygen saturation b93% at rest; - partial arterial oxygen pressure (PaO2)/fraction of inspiration oxygen (FiO2) b300 mmHg.   2. Critical patients should meet one of the three conditions:   - respiratory failure, requiring mechanical ventilation; - shock; - (iii) multiple organ failure, requiring intensive care management. |
| Feng, Y. et al. | Fifth version of the guidelines issued by the National Health Commission of China on Diagnosis and Treatment of COVID-19 | Severity was classified as follows:  1. Mild type: The clinical symptoms are mild, with no abnormal radiological findings.  2. Moderate type: Fever, cough, and other symptoms are present with pneumonia on chest CT.  3. Severe type: The disease is classified as severe if one of the following conditions is met:   - Respiratory distress, respiratory rate ≥30/min. - Oxygen saturation on room air at rest ≤93%. - Partial pressure of oxygen in arterial blood/FiO2 ≤300 mm Hg.   4. Critical type: One of the following conditions has to be met:   - Respiratory failure occurs and mechanical ventilation is required. - Shock occurs. - Other organ dysfunction is present, requiring ICU monitoring and treatment. |
| Guan, W. et al. | Based on 2007 American Thoracic Society/Infectious Disease Society of America guidelines (88). | Severe cases denoted at least one major criterion:   - septic shock requiring vasoactive medications, or - respiratory failure requiring mechanical ventilation.   Or at least three minor criteria:   - respiratory rate ⩾30 breaths·min−1, - oxygen index ⩽250, - multiple lobe infiltration, - delirium or loss of consciousness, - blood urea nitrogen ⩾20 mg·dL−1 , - blood leukocyte count ⩽4000 cells·dL−1, - blood platelet count ⩽100000 cells·dL−1, - body temperature <36°C, a - hypotension necessitating vasoactive drugs for maintaining blood pressure. |
| Li, K. et al. | Diagnosis and Treatment of Novel Coronavirus Pneumonia (Fifth Trial Version) of China | The severe/critical patients met any of the following conditions:   - respiratory rate of 30 breaths per minute or greater; - finger of oxygen saturation of 93% or less in a resting state; - arterial oxygen tension (Pao2)/inspiratory oxygen fraction (Fio2) of 300 mm Hg or less (1 mm Hg = 0.133 kPa); - respiratory failure occurred and mechanical ventilation required; - shock occurred; and - patients with other organ failure needed intensive care unit monitoring and treatment. |
| Li, X. et al. | Based on the official clinical practice guideline of the American Thoracic Society and Infectious Disease Society of America (87). | On the basis of whether or not requiring: ventilatory support on admission |
| Li, Y. et al. | Based on "Seventh revised trial Version of the Novel Coronavirus Pneumonia Diagnosis and Treatment Guidance" | 1. Mild stage: the clinical symptoms were mild, and no signs of pneumonia were found on imaging;  2. Common stage: fever, respiratory tract and other symptoms, imaging findings of pneumonia;  3. Severe stage, 1 of the following criteria existed:   - shortness of breath, respiratory rate ≥30 times/min; - in resting state, oxygen saturation is less than 93%; - PaO2/FiO2 ≤300 mmHg. CT imaging showed significant lesion progression >50% within 24 to 48 h;   4. Critical stage, one of the following occurred:   - Respiratory failure requiring mechanical ventilation; - Shock; - Complicated with other organ failure requiring ICU care |
| Liu, Jing. et al. | Based on "Fifth revised trial Version of the Novel Coronavirus Pneumonia Diagnosis and Treatment Guidance" | 1. Mild patients met all following conditions:   - Epidemiological history, - Fever or other respiratory symptoms, - Typical CT image abnormities of viral pneumonia, and - Positive result of RT-PCR for SARS-CoV-2 RNA.   2. Severe patients additionally met at least one of the following conditions:   - Shortness of breath, respiratory rate ≥30 times/min, - Oxygen saturation (Resting state) ≤93%, or - (3) PaO2 / FiO2 ≤300 mmHg. |
| Liu, Jingyuan. et al. | Based on "Fourth revised trial Version of the Novel Coronavirus Pneumonia Diagnosis and Treatment Guidance" | 1. Mild, with fever, respiratory tract symptoms, and imaging shows pneumonia.  2. Moderate, meet any of the following:   - respiratory distress, respiratory rate ≥ 30 beats / min; - in the resting state, means oxygen saturation ≤ 93%; - arterial blood oxygen partial pressure / oxygen concentration ≤ 300mmHg (1mmHg = 0.133kPa).   3. Severe, one of the following conditions:   - respiratory failure occurs and requires mechanical ventilation; - Shock occurs; - ICU admission is required for combined organ failure. |
| Liu, W. et al. | Based on “Fourth revised trial version of the Diagnosis and Treatment Protocol for Novel Coronavirus Infection-Induced Pneumonia” | 1. Common: fever, respiratory tract infection symptoms, and so on, with imaging indicating pneumonia;  2. Severe (any of the following conditions):   - respiratory distress, RR ≥30 breaths/min; - oxygen saturation ≤93% at rest; - partial pressure of oxygen (PaO2)/fraction of inspired oxygen ≤300 mmHg (1 mmHg = 0.133 kPa);   3. Critical (any of the following conditions):   - respiratory failure and a requirement for mechanical ventilation; - shock; - concomitant failure of other organs and requirement for ICU monitoring and treatment. |
| Liu, Y. et al. | Based in The Berlin definition to determine the presence and severity of ARDS (Acute Respiratory Distress Syndrome) (89). | Exclusive categories of ARDS based on degree of hypoxemia:   - mild (200 mm Hg < PaO2/FIO2 ≤ 300 mm Hg), - moderate (100 mm Hg < PaO2/FIO2 ≤ 200 mm Hg), and - severe (PaO2/FIO2 ≤ 100 mm Hg).   Ancillary variables for severe ARDS:   - radiographic severity, - respiratory system compliance (≤40 mL/cm H2O), - positive end-expiratory pressure (≥10 cm H2O), and - corrected expired volume per minute (≥10 L/min). |
| Ma, J. et al. | National Health Commission of China. New coronavirus pneumonia prevention and control program (7th edition) | 1. Mild cases: The clinical symptoms were mild, and there was no sign of pneumonia on imaging.  2. Moderate cases: Showing fever and respiratory symptoms with radiological findings of pneumonia.  3. Severe cases:  Adult cases meeting any of the following criteria:   - Respiratory distress (≧30 breaths/ min); - Oxygen saturation≤93% at rest; - Arterial partial pressure of oxygen (PaO2)/ fraction of inspired oxygen (FiO2)≦ - 300mmHg (l mmHg=0.133kPa).   Child cases meeting any of the following criteria:   - Tachypnea (RR ≥ 60 breaths/min for infants aged below 2 months; RR ≥ 50 BPM for - infants aged 2-12 months; RR ≥ 40 BPM for children aged 1-5 years, and RR ≥ 30 BPM - for children above 5 years old) independent of fever and crying; - Oxygen saturation ≤ 92% on finger pulse oximeter taken at rest; - Laboured breathing (moaning, nasal fluttering, and infrasternal, supraclavicular and - intercostal retraction), cyanosis, and intermittent apnea; - Lethargy and convulsion; - Difficulty feeding and signs of dehydration.   4. Critical cases: Cases meeting any of the following criteria:   - Respiratory failure and requiring mechanical ventilation; - Shock; - With other organ failure that requires ICU care. |
| Mao, L. et al. | Based on the official clinical practice guideline of the American Thoracic Society and Infectious Disease Society of America (87). | Minor criteria   - Respiratory rate ≥ 30 breaths/min - PaO2/FiO2 ratio ≤ 250 - Multilobar infiltrates - Confusion/disorientation - Uremia (blood urea nitrogen level ≥ 20 mg/dl) - Leukopenia* (white blood cell count < 4,000 cells/μl) - Thrombocytopenia (platelet count < 100,000/μl) - Hypothermia (core temperature < 36°C) - Hypotension requiring aggressive fluid resuscitation   Major criteria   - Septic shock with need for vasopressors - Respiratory failure requiring mechanical ventilation |
| Qin, C. et al. | Based on "Fifth revised trial Version of the Novel Coronavirus Pneumonia Diagnosis and Treatment Guidance" | Severe-type met the following criteria:   - Respiratory distress with the respiratory rate over 30 per minute; - Oxygen saturation ≤ 93% in the resting state; - 3. Arterial blood oxygen partial pressure (PaO2) / oxygen concentration (FiO2) ≤300mmHg. |
| Sun, S. et al. | Based on “Seventh revised version of the Novel Coronavirus Pneumonia Diagnosis and Treatment Interim Guidance” | 1. Common cases: Those who have fever, respiratory tract symptoms, and pneumonia on imaging.  2. Severe cases: Those who have one of the following three clinical manifestations:   - shortness of breath with RR > 30 times/min; - mean oxygen saturation ≤ 93% in resting state; - partial pressure of arterial oxygen (PaO2)/oxygen Concentration (FiO2) ≤ 300 mmHg (1 mmHg = 0.133 kPa).   Or those whose pulmonary imaging shows that the lesions have progressed more than 50% within 24–48 h. |
| Wan, S. et al. | Based on "Fifth revised trial Version of the Novel Coronavirus Pneumonia Diagnosis and Treatment Guidance" | 1. Mild group had mild clinical symptoms and no pneumonia on imaging.  2. Normal group had symptoms of fever, respiratory tract symptoms, and imaging showed pneumonia.  3. Severe group had:   - respiratory distress, - RR ≥ 30 beats/minute in a resting state, - a mean oxygen saturation of ≤93%, and - an arterial blood oxygen partial pressure (PaO2)/oxygen concentration (FiO2) ≤ 300 mm Hg.   4. Critical group had:   - respiratory failure and required mechanical ventilation, - the occurrence of shock, and - (iii) the combined failure of other organs that required ICU monitoring and treatment. |
| Wang, R. et al. | Based in The Berlin definition to determine the presence and severity of ARDS (Acute Respiratory Distress Syndrome) (89). | 1. within 1 week of a known clinical insult or new or worsening respiratory symptoms;  2. bilateral opacities-not fully explained by effusions, lobar/lung collapse, or nodules;  3. respiratory failure not fully explained by cardiac failure or fluid overload need objective assessment (eg, echocardiography) to exclude hydrostatic edema if no risk factor present;  4. patients were classified as:   - mild (200 mmHg < PaO2/FIO2≤300 mmHg with PEEP or CPAP ≥ 5 cmH2O), - moderate (100 mmHg < PaO2/FIO2≤200 mmHg with PEEP ≥ 5 cmH2O) and - severe (PaO2/FIO2≤100 mmHg with PEEP ≥ 5 cmH2O) according to oxygenation index. |
| Wei, X. et al. | Chinese Centre for Disease Control (CDC) guidelines and a previous literature (90). | 1. Mild: non-pneumonia and mild pneumonia.  2. Severe:   - dyspnoea, - respiratory frequency 30/min, - blood oxygen saturation 93%, - partial pressure of arterial oxygen to fraction of inspired oxygen ratio <300, and/or - lung infiltrates >50% within 24 to 48 hours.   3. Critical:   - respiratory failure, - septic shock, and/or - multiple organ dysfunction or failure. |
| Wu, C. et al. | World Health Organization interim guidance to define ARDS (91). | 1. Mild cases  2. Moderate disease: Pneumonia  3. Severe disease: Severe Pneumonia  4. Critical disease: Acute respiratory distress syndrome |
| Xie, H. et al. | Based on "Sixth revised trial Version of the Novel Coronavirus Pneumonia Diagnosis and Treatment Guidance" | Severe group included:   - Respiratory distress, breathing frequency ≥ 30 breaths/min - In resting state, means oxygen saturation ≤ 93% and - (iii) Arterial blood oxygen partial pressure/oxygen concentration ≤ 300 mm Hg. |
| Xu, Y. | Based on "Fifth revised trial Version of the Novel Coronavirus Pneumonia Diagnosis and Treatment Guidance" | 1. Mild with slight clinical symptoms but no imaging presentations of pneumonia.  2. Common with fever, respiratory symptoms and imaging presentations of pneumonia.  3. Severe type with any of the following:   - respiratory distress with RR> 30 times/minutes, - oxygen saturation at rest <93%, or - PaO2/FiO2<300 mmHg (1 mmHg=0.133 kPa).   4. Critically severe type with any of the following:   - respiratory failure needing mechanical ventilation, - shock, or - combination with other organ failure needing ICU intensive care. |
| Yao, Q. et al. | Based on the official clinical practice guideline of the American Thoracic Society and Infectious Disease Society of America (87). | Severe COVID‑19 should reach either 1 major criterion or 3 or more minor criteria.  Minor criteria included:   - respiratory rate more than 30 breaths per minute, - the ratio of oxygen arterial pressure to oxygen inspiratory fraction lower than 250, - multilobar infiltrates confusion or disorientation, - blood urea nitrogen level more than 7.1 mmol/l, - white blood cell (WBC) count less than 4×109/l, - platelet count less than 100×1012/l, - core temperature lower than 36 ºC, - hypotension requiring aggressive fluid resuscitation.   Major criteria included:   - septic shock with need for vasopressors, or - mechanical ventilation. |
| Zhang, G. et al. | Based on the official clinical practice guideline of the American Thoracic Society and Infectious Disease Society of America (87). | Severe or critical COVID-19 patients were defined according to the following criteria: fever plus one of these conditions, including respiratory rate ≥ 30 breaths/min, severe respiratory distress, SpO2 ≤ 93% on room air, occurrence of respiratory failure requiring mechanical ventilation, shock and other organ failure. |
| Zhang, J. et al. | World Health Organization (WHO) interim guidance. | 1. Severely ill: one with any of the following symptoms:   - Respiratory distress, respiratory rate (RR) acuity of 30 breaths/min. - Oxygen saturation during resting state of 93% or less. - Arterial blood oxygen partial pressure (PaO2)/(FiO2) ≤ 300 mmHg (high-altitude region (more than 1000 m above sea level, according to the following formula for correction: PaO2/FiO2 × 760 (mmHg)/atmosphere)).   2. Critically ill: those with any of the characteristics:   - Respiratory failure and need for mechanical ventilation. - Shock. - Combination of failure of other organ functions, followed by the need for ICU admission. |
| Zhang, Jin-jin. et al. | Based on "Diagnosis and Treatment Guideline for SARS-CoV-2" issued by the Chinese National Health Committee (version 3-5). | 1. Severe: patients with one of the following criteria:   - Respiratory distress with respiratory frequency ≥ 30/min - Pulse oximeter oxygen saturation ≤ 93% at rest - Oxygenation index (artery partial pressure of oxygen/inspired oxygen fraction, PaO_2_/FiO_2_) ≤300 mm Hg. |
| Zhang, Jixiang. et al. | Interim guidelines from the World Health Organization and the National Health Commission of China. | 1. Mild: slight clinical symptoms without imaging findings of pneumonia.  2. Moderate: fever or respiratory symptoms.  3. Severe: having the following conditions:   - respiratory distress and a respiratory rate >30 times per minute, - fingertip blood oxygen saturation <93% at rest, and - partial arterial oxygen pressure (PaO2)/fraction of inspiration oxygen (FiO2) ≤300 mmHg.   4. Critical: having one of the following conditions:   - respiratory failure requiring mechanical ventilation, - shock, and - other organ failure requiring ICU treatment. |
| Zhang, Y. et al. | Based on "Fifth revised trial Version of the Novel Coronavirus Pneumonia Diagnosis and Treatment Guidance" | 1. Mild: patients with COVID-19 who had fever, respiratory symptoms and pneumonia from imaging.  2. Severe disease: patients with COVID-19 who in addition developed:   - significant respiratory distress (RR>30/min), - blood oxygen saturation<93%; - arterial oxygen partial pressure (PaO2)/Fraction of inspire O2 (FiO2) <300mmHg; - respiratory failure with mechanical ventilation; - shock; or - other organ failure need intensive care in ICU |
| Zheng, F. et al. | Based on "Fifth revised trial Version of the Novel Coronavirus Pneumonia Diagnosis and Treatment Guidance" | Severity was classified as follows:  1. Mild type: The clinical symptoms are mild, with no abnormal radiological findings.  2. Moderate type: Fever, cough, and other symptoms are present with pneumonia on chest CT.  3. Severe type: The disease is classified as severe if one of the following conditions is met:   - Respiratory distress, respiratory rate ≥30/min. - Oxygen saturation on room air at rest ≤93%. - Partial pressure of oxygen in arterial blood/FiO2 ≤300 mm Hg.   4. Critical type: One of the following conditions has to be met:   - Respiratory failure occurs and mechanical ventilation is required. - Shock occurs. - Other organ dysfunction is present, requiring ICU monitoring and treatment. |
| Zheng, KI. et al. | Based on "Seventh revised trial Version of the Novel Coronavirus Pneumonia Diagnosis and Treatment Guidance" | 1. Mild stage: the clinical symptoms were mild, and no signs of pneumonia were found on imaging;  2. Common stage: fever, respiratory tract and other symptoms, imaging findings of pneumonia;  3. Severe stage, 1 of the following criteria existed:   - shortness of breath, respiratory rate ≥30 times/min; - in resting state, oxygen saturation is less than 93%; - PaO2/FiO2 ≤300 mmHg. CT imaging showed significant lesion progression >50% within 24 to 48 h;   4. Critical stage, one of the following occurred:   - Respiratory failure requiring mechanical ventilation; - Shock; - Complicated with other organ failure requiring ICU care |
| Zheng, S. et al. | Based on “Sixth edition of the Guideline for Diagnosis and Treatment of SARS-CoV-2” | 1. Mild cases include non-pneumonia or mild pneumonia.  2. Severe disease refers to:   - dyspnoea, - respiratory rate ≥30/min, - blood oxygen saturation ≤93%, - partial pressure of arterial oxygen to fraction of inspired oxygen ratio <300, - or lung infiltrates >50% within 24 to 48 hours. |
| Zhu, Z. et al. | Based on “Sixth edition of the Guideline for Diagnosis and Treatment of SARS-CoV-2” | 1. Severe: patients should meet at least one of the following criterions:   - shortness of breath with respiration rate (RR) ≥30 times/min. - oxygen saturation ≤93% in resting state. - partial pressure of arterial oxygen (PaO2)-to-fraction of inspired oxygen (FiO2) ratio ≤300 mm Hg. - lesion progression >50% within 24-48 hours on pulmonary imaging were also recognized as severe cases.   2. Critical: patients with one of the following conditions:   - respiratory failure and require mechanical ventilation. - shock occurred. - combined with other organ failure and treated in intensive care unit. |
| Zou, Y. et al. | Based on "Seventh revised trial Version of the Novel Coronavirus Pneumonia Diagnosis and Treatment Guidance" | 1. Mild stage: the clinical symptoms were mild, and no signs of pneumonia were found on imaging;  2. Common stage: fever, respiratory tract and other symptoms, imaging findings of pneumonia;  3. Severe stage, 1 of the following criteria existed:   - shortness of breath, respiratory rate ≥30 times/min; - in resting state, oxygen saturation is less than 93%; - PaO2/FiO2 ≤300 mmHg. CT imaging showed significant lesion progression >50% within 24 to 48 h;   4. Critical stage, one of the following occurred:   - Respiratory failure requiring mechanical ventilation; - Shock; - Complicated with other organ failure requiring ICU care |

## Results of the risk of bias assessment

**Table 4.** Results of the risk of bias assessment

| **First Author** | **Bias due to confounding** | **Bias due to selection of participants and follow-up** | **Bias due to misclassification of exposure** | **Bias due to missing data** | **Bias in measurement of outcome** | **Bias in selection of the reported** |
| --- | --- | --- | --- | --- | --- | --- |
| **Criteria*** | **low** if stratification/adjustment for age - **moderate** if age information - **high** if no age information | **low** if follow-up well documented for all risk groups up to intended follow-up time - **moderate** if not well reported for all groups - **high** if different follow-up time for different groups or follow-up not until endpoint for all groups | **low** if medical records - **moderate** if self-reported - **high** if unclear source | **low** if outcome data available for all and missing data reported - **moderate** if some missing data reported but not all - **high** if participants excluded on large scale for missing data | **low** if outcome definition reported and well classified, **moderate** if no report, but clear meaning, **high** if no report available | **low** if subgroup reporting seems adequate and not selective, **high** if selective subgroup reporting |
| Cai, Q. et al. | moderate | low | low | high | low | low |
| Cao, J. et al. | moderate | low | low | high | low | low |
| CDC COVID-19 Response Team | moderate | moderate | low (public health departments) | low | low | low |
| Chen, G. et al. | moderate | low | low | high | low | low |
| Chen, Q. et al. | moderate | moderate | low | high | low | low |
| Chen, T. et al. | low | moderate | low | low | low | low |
| Cheng, Y. et al. | low | low | low | high | low | low |
| Deng, G. et al. | moderate | moderate | low | moderate | low | low |
| Deng, Q. et al. | moderate | low | low | high | low | low |
| Du, R. et al. | moderate | low | low | high | low | low |
| Duanmu, Y. et al. | moderate | moderate | low | high | low | low |
| Fan, J. et al. | moderate | low | low | high | low | low |
| Feng, Y. et al. | moderate | low | low | high | low | low |
| Goyal, P. et al. | moderate | low | low | high | low | low |
| Guan, W. et al. Eur Respir J | low | moderate | low | moderate | moderate | high |
| Guan, W. et al. N Engl J Med | moderate | low | low | low | low | low |
| Guo, T. et al. | moderate | moderate | low | high | low | low |
| Huang, C. et al | moderate | low | low | low | low | low |
| Ji, D. et al. | moderate | low | low | high | low | low |
| Ji, Dong. et al. | moderate | low | low | high | low | low |
| Kalligeros, M. et al. | moderate | low | low | high | low | low |
| Lei, S. et al. | moderate | low | low | low | low | low |
| Li, K. et al. | moderate | moderate | low | high | low | low |
| Li, X. et al. | low | low | low | moderate | low | low |
| Li, Y. et al | moderate | low | low | low | low | low |
| Liang, W. et al | low | moderate | low | moderate | moderate | low |
| Liu, Jing. et al. | moderate | moderate | low | high | high | low |
| Liu, Jingyuan. et al. | moderate | moderate | low | high | low | low |
| Liu, W. et al | moderate | low | low | low | low | low |
| Liu, Y. et al. | moderate | moderate | low | high | low | low |
| Ma, J. et al. | moderate | moderate | low | high | low | low |
| Mao, L. et al. | moderate | moderate | low | low | low | low |
| McMichael, T. et al. | moderate | moderate | low | moderate | moderate | low |
| Nikpouraghdam, M. et al. | low | moderate | low | low | low | low |
| Quin, C. et al | low | moderate | low | low | low | low |
| Ruan, Q. et al. | moderate | moderate | low | high | low | low |
| Shi, Y. et al | low | low | low | low | low | moderate |
| Simonnet, A. et al. | moderate | moderate | low | high | low | low |
| Sun, S. et al | moderate | low | low | high | low | low |
| Tang, N. et al. | moderate | low | low | high | low | low |
| The Novel Coronavirus Pneumonia Emergency Response Epidemiology Team | moderate | moderate | moderate | moderate | low | low |
| Tian, S. et al. | moderate | low | low | low | low | low |
| Tomlins, J. et al. | moderate | low | low | high | low | low |
| Wan, S. et al | moderate | low | low | low | moderate | low |
| Wang, D. et al | moderate | low | low | low | low | low |
| Wang, Dawei et al. | low | moderate | low | high | low | low |
| Wang, L. et al | moderate (only elderly included) | moderate | low | low | low | low |
| Wang, R. et al. | moderate | low | low | high | low | low |
| Wang, X. et al. | moderate | low | low | high | low | low |
| Wang, Y. et al. | moderate | moderate | low | moderate | low | low |
| Wei, X. et al. | moderate | moderate | low | high | low | low |
| Wie, YY. et al. | moderate | moderate | high | high | low | low |
| Wu, C. et al. | moderate | low | low | moderate | low | low |
| Xie, H. et al. | moderate | moderate | low | high | low | low |
| Xu, Y. | moderate | moderate | low | high | low | low |
| Yan, Y. et al. | moderate | low | low | low | low | low |
| Yang, X. et al. | moderate | low | low | low | low | low |
| Yao, Q. et al. | moderate | low | low | high | low | low |
| Yu, X. et al. | moderate | low | low | low | low | low |
| Yuan, M. et al. | moderate | moderate | low | high | low | low |
| Zhang, G. et al | moderate | low | low | low | low | low |
| Zhang, J. et al | low | low | low/moderate | low | low | low |
| Zhang, J. et al. | moderate | moderate | low | high | low | low |
| Zhang, Jixiang. et al. | low | low | low | high | low | low |
| Zhang, Y. et al. | moderate | moderate | low | high | low | low |
| Zhao, X. et al. | moderate | low | low | high | low | low |
| Zheng, F. et al. | moderate | moderate | low | high | low | low |
| Zheng, KI. et al. | moderate | moderate | low | high | low | low |
| Zheng, S. et al. | moderate | moderate | low | high | low | low |
| Zhou, F. et al. | low | moderate | low | moderate | low | low |
| Zhu, Z. et al. | low | moderate | low | high | low | low |
| Zou, Y. et al | moderate | moderate | low | high | low | low |

*Adapted version of ROBINS-I: a tool for assessing risk of bias in non-randomized studies of interventions.

## Meta-analysis by individual factor and outcome

### Comorbidities

#### Asthma

| Study | RR | [95% Conf. Interval] | |
| --- | --- | --- | --- |
| Severe clinical course of disease |  |  |  |
| Li X. et al. | 1.225 | 0.596 | 2.518 |
| Duanmu Y. et al. | 1.286 | 0.464 | 3.559 |
| Sub-total |  |  |  |
| D+L pooled RR | 1.245 | 0.691 | 2.242 |
|  |  |  |  |
| ICU |  |  |  |
| Goyal, P. et al. | 1.056 | 0.699 | 1.596 |
| Sub-total |  |  |  |
| D+L pooled RR | 1.056 | 0.699 | 1.596 |
|  |  |  |  |
| Death |  |  |  |
| Tomlins J. et al. | 0.881 | 0.330 | 2.354 |
| Sub-total |  |  |  |
| D+L pooled RR | 0.881 | 0.330 | 2.354 |
|  |  |  |  |
| Overall |  |  |  |
| D+L pooled RR | 1.088 | 0.790 | 1.497 |

#### Cancer

| Study | RR | [95% Conf. Interval] | |
| --- | --- | --- | --- |
| Severe clinical course of disease |  |  |  |
| Feng, Y. et al. | 2.313 | 1.399 | 3.826 |
| Li X. et al. | 1.174 | 0.827 | 1.665 |
| Liu, Jing. et al. | 0.750 | 0.129 | 4.356 |
| Mao, L. et al. | 0.931 | 0.459 | 1.889 |
| Qin, C. et al. | 1.134 | 0.808 | 1.591 |
| Wan, S. et al. | 2.655 | 1.417 | 4.977 |
| Yao Q. et al. | 4.417 | 1.001 | 19.495 |
| Zhang Jixiang. et al | 1.281 | 0.968 | 1.696 |
| Zhang Y. et al. | 1.727 | 0.610 | 4.893 |
| Zhao X. et al. | 2.095 | 0.890 | 4.935 |
| Zhu, Z. et al. | 1.627 | 0.265 | 9.999 |
| Duanmu Y. et al. | 1.406 | 0.273 | 7.244 |
| Sub-total |  |  |  |
| D+L pooled RR | **1.463** | **1.177** | **1.819** |
|  |  |  |  |
| ICU |  |  |  |
| Lei, S. et al. | 1.389 | 0.652 | 2.959 |
| Liang, W. et al. | 4.930 | 2.697 | 9.012 |
| Liu, W. et al. | 4.111 | 1.295 | 13.056 |
| Wang, D. et al. | 1.600 | 0.707 | 3.619 |
| Kalligeros M. et al. | 1.649 | 0.977 | 2.782 |
| Sub-total |  |  |  |
| D+L pooled RR | **2.304** | **1.333** | **3.984** |
|  |  |  |  |
| Death |  |  |  |
| Chen, T. et al. | 1.766 | 1.081 | 2.884 |
| Deng G. et al. | 2.926 | 1.337 | 6.406 |
| The Novel Coronaviru | 2.457 | 1.127 | 5.359 |
| Yang, X. et al. | 0.806 | 0.198 | 3.279 |
| Yao Q. et al. | 4.818 | 1.081 | 21.476 |
| Zhang Jixiang. et al | 1.932 | 0.281 | 13.295 |
| Nikpouraghdam M. et | 0.728 | 0.108 | 4.896 |
| Sub-total |  |  |  |
| D+L pooled RR | **2.019** | **1.445** | **2.822** |
|  |  |  |  |
| Overall |  |  |  |
| D+L pooled RR | **1.769** | **1.443** | **2.167** |

#### Cardiovascular disease

| Study | RR | [95% Conf. Interval] | |
| --- | --- | --- | --- |
| Severe clinical course of disease |  |  |  |
| Feng, Y. et al. | 1.831 | 1.240 | 2.704 |
| Guan, W. et al. | 2.218 | 1.524 | 3.228 |
| Li, Y. et al. | 0.656 | 0.110 | 3.902 |
| Liu, J. et al. | 2.773 | 1.127 | 6.820 |
| Qin, C. et al. | 1.442 | 1.237 | 1.680 |
| Shi, Y. et al. | 3.846 | 1.678 | 8.819 |
| Wan, S. et al. | 3.227 | 2.125 | 4.900 |
| Wang, X. et al. | 3.498 | 1.668 | 7.338 |
| Wei X. et al. | 1.642 | 1.229 | 2.192 |
| Wei YY. et al. | 1.813 | 0.876 | 3.753 |
| Yu X. et al. | 2.100 | 1.119 | 3.940 |
| Zhang G. et al | 2.800 | 1.804 | 4.344 |
| Zhang Jixiang. et al | 1.434 | 1.285 | 1.600 |
| Zhu, Z. et al. | 2.881 | 0.839 | 9.897 |
| Deng Q. et al. | 1.270 | 0.896 | 1.802 |
| Li K. et al. | 2.541 | 1.068 | 6.043 |
| Li X. et al. | 1.756 | 1.466 | 2.104 |
| Xie, H. et al. | 0.791 | 0.236 | 2.656 |
| Zhang, J. et al. | 1.407 | 0.718 | 2.761 |
| Zhang, J. et al. | 2.705 | 1.239 | 5.905 |
| Zheng S. et al. | 1.250 | 1.008 | 1.550 |
| Zheng, F. et al. | 2.804 | 0.995 | 7.899 |
| CDC COVID-19 Respons | 3.065 | 2.721 | 3.453 |
| Sub-total |  |  |  |
| D+L pooled RR | **1.975** | **1.629** | **2.394** |
|  |  |  |  |
| ICU |  |  |  |
| Huang, C. et al. | 1.750 | 0.673 | 4.554 |
| Lei, S. et al. | 2.571 | 1.393 | 4.748 |
| Liu, Y. et al. | 0.874 | 0.363 | 2.104 |
| Wang, D. et al. | 1.967 | 1.094 | 3.537 |
| Guan, W. et al. | 3.905 | 1.851 | 8.239 |
| Goyal, P. et al. | 1.495 | 1.076 | 2.076 |
| Kalligeros M. et al. | 1.456 | 0.931 | 2.276 |
| CDC COVID-19 Respons | 4.090 | 3.397 | 4.923 |
| Sub-total |  |  |  |
| D+L pooled RR | **2.071** | **1.333** | **3.215** |
|  |  |  |  |
| Death |  |  |  |
| Cao, J. et al. | 2.425 | 0.758 | 7.760 |
| Chen, T. et al. | 1.800 | 1.318 | 2.459 |
| Deng G. et al. | 6.749 | 5.404 | 8.429 |
| Fan J. et al. | 3.200 | 0.595 | 17.224 |
| Guan, W. et al. | 4.943 | 2.430 | 10.053 |
| Guo, T. et al. | 3.798 | 2.163 | 6.668 |
| Li, Y. et al. | 1.313 | 0.194 | 8.888 |
| The Novel Coronaviru | 4.958 | 4.045 | 6.076 |
| Wang Dawei et al. | 4.218 | 2.033 | 8.750 |
| Wang, L. et al. | 2.575 | 1.676 | 3.956 |
| Wang, Y. et al. | 1.506 | 1.097 | 2.068 |
| Yao Q. et al. | 5.200 | 1.657 | 16.316 |
| Zhang Jixiang. et al | 5.409 | 2.437 | 12.007 |
| Zhou, F. et al. | 3.468 | 2.428 | 4.954 |
| Nikpouraghdam M. et | 1.347 | 0.529 | 3.425 |
| Sub-total |  |  |  |
| D+L pooled RR | **3.250** | **2.335** | **4.522** |
|  |  |  |  |
| Composite Endpoint |  |  |  |
| Deng Q. et al. | 1.552 | 0.766 | 3.144 |
| Sub-total |  |  |  |
| D+L pooled RR | 1.552 | 0.766 | 3.144 |
|  |  |  |  |
| Overall |  |  |  |
| D+L pooled RR | **2.317** | **1.947** | **2.758** |

#### Cerebrovascular disease

| Study | RR | [95% Conf. Interval] | |
| --- | --- | --- | --- |
| Severe clinical course of disease |  |  |  |
| Mao, L. et al. | 1.147 | 0.651 | 2.020 |
| Feng, Y. et al. | 2.113 | 1.314 | 3.399 |
| Guan, W. et al. | 3.264 | 2.240 | 4.755 |
| Zhang G. et al | 3.433 | 2.296 | 5.133 |
| Zhang, J. et al. | 1.778 | 0.641 | 4.930 |
| Zheng, F. et al. | 1.353 | 0.240 | 7.626 |
| Sub-total |  |  |  |
| D+L pooled RR | **2.247** | **1.509** | **3.346** |
|  |  |  |  |
| ICU |  |  |  |
| Du, R. et al. | 0.811 | 0.515 | 1.276 |
| Lei, S. et al. | 2.037 | 1.062 | 3.907 |
| Liu, Y. et al. | 2.033 | 1.516 | 2.727 |
| Wang, D. et al. | 3.743 | 2.420 | 5.789 |
| Sub-total |  |  |  |
| D+L pooled RR | 1.884 | 0.895 | 3.967 |
|  |  |  |  |
| Death |  |  |  |
| Cao, J. et al. | 3.200 | 1.268 | 8.073 |
| Guan, W. et al. | 7.091 | 3.274 | 15.356 |
| Wang Dawei et al. | 3.156 | 1.261 | 7.902 |
| Yang, X. et al. | 1.691 | 1.234 | 2.317 |
| Wang, L. et al. | 2.753 | 1.655 | 4.580 |
| Yuan, M. et al. | 2.132 | 0.824 | 5.511 |
| Tomlins J. et al. | 1.208 | 0.340 | 4.297 |
| Sub-total |  |  |  |
| D+L pooled RR | **2.647** | **1.725** | **4.062** |
|  |  |  |  |
| Composite Endpoint |  |  |  |
| Guan, W. et al. | 4.588 | 1.917 | 10.982 |
| Sub-total |  |  |  |
| D+L pooled RR | 4.588 | 1.917 | 10.982 |
|  |  |  |  |
| Overall |  |  |  |
| D+L pooled RR | **2.326** | **1.812** | **2.987** |

#### COPD

| Study | RR | [95% Conf. Interval] | |
| --- | --- | --- | --- |
| Severe clinical course of disease |  |  |  |
| Deng Q. et al. | 1.266 | 0.704 | 2.276 |
| Li K. et al. | 2.971 | 1.679 | 5.258 |
| Li X. et al. | 1.586 | 1.201 | 2.095 |
| Li, Y. et al. | 3.200 | 1.332 | 7.688 |
| Liu, J. et al. | 2.400 | 1.029 | 5.600 |
| Liu, W. et al. | 3.800 | 0.847 | 17.055 |
| Qin, C. et al. | 1.191 | 0.853 | 1.664 |
| Zhang G. et al | 2.810 | 1.520 | 5.196 |
| Zhao X. et al. | 2.314 | 0.986 | 5.431 |
| Zheng, F. et al. | 1.845 | 0.567 | 6.006 |
| Duanmu Y. et al. | 3.191 | 1.331 | 7.655 |
| Sub-total |  |  |  |
| D+L pooled RR | **1.982** | **1.535** | **2.559** |
|  |  |  |  |
| ICU |  |  |  |
| Guan, W. et al. | 10.568 | 6.173 | 18.094 |
| Huang, C. et al. | 2.460 | 0.976 | 6.198 |
| Liu, Y. et al. | 1.029 | 0.379 | 2.797 |
| Goyal, P. et al. | 1.061 | 0.574 | 1.962 |
| Sub-total |  |  |  |
| D+L pooled RR | 2.352 | 0.564 | 9.810 |
|  |  |  |  |
| Death |  |  |  |
| Li, Y. et al. | 6.000 | 1.343 | 26.808 |
| Wang Dawei et al. | 1.926 | 0.368 | 10.074 |
| Wang, L. et al. | 3.085 | 1.919 | 4.959 |
| Wang, Y. et al. | 2.221 | 1.687 | 2.924 |
| Zhou, F. et al. | 2.467 | 1.336 | 4.555 |
| Tomlins J. et al. | 2.125 | 0.883 | 5.114 |
| Sub-total |  |  |  |
| D+L pooled RR | **2.436** | **1.972** | **3.009** |
|  |  |  |  |
| Composite Endpoint |  |  |  |
| Deng Q. et al. | 0.900 | 0.160 | 5.048 |
| Sub-total |  |  |  |
| D+L pooled RR | 0.900 | 0.160 | 5.048 |
|  |  |  |  |
| Overall |  |  |  |
| D+L pooled RR | **2.279** | **1.766** | **2.942** |

#### Chronic Renal Failure

| Study | RR | [95% Conf. Interval] | |
| --- | --- | --- | --- |
| Severe clinical course of disease |  |  |  |
| Chen Q. et al. | 1.127 | 0.223 | 5.696 |
| Cheng, Y. et al. | 1.290 | 1.047 | 1.591 |
| Li X. et al. | 1.225 | 0.733 | 2.047 |
| Mao, L. et al. | 0.806 | 0.257 | 2.529 |
| Shi, Y. et al. | 2.918 | 0.877 | 9.711 |
| Zhang Jixiang. et al | 1.245 | 0.972 | 1.593 |
| Zhao X. et al. | 2.314 | 0.986 | 5.431 |
| CDC COVID-19 Response | 3.290 | 2.801 | 3.865 |
| Sub-total |  |  |  |
| D+L pooled RR | **1.631** | **1.030** | **2.581** |
|  |  |  |  |
| ICU |  |  |  |
| Cheng, Y. et al. | 1.287 | 0.734 | 2.256 |
| Duanmu Y. et al. | 3.133 | 1.577 | 6.225 |
| Kalligeros M. et al. | 0.836 | 0.370 | 1.891 |
| CDC COVID-19 Response | 4.556 | 3.569 | 5.816 |
| Sub-total |  |  |  |
| D+L pooled RR | 2.057 | 0.872 | 4.851 |
|  |  |  |  |
| Death |  |  |  |
| Cheng, Y. et al. | 2.557 | 1.816 | 3.600 |
| Wang Dawei et al. | 1.926 | 0.368 | 10.074 |
| Nikpouraghdam M. et | 2.081 | 0.735 | 5.887 |
| Sub-total |  |  |  |
| D+L pooled RR | **2.481** | **1.804** | **3.414** |
|  |  |  |  |
| Composite Endpoint |  |  |  |
| Yu X. et al. | 2.119 | 0.899 | 4.992 |
| Sub-total |  |  |  |
| D+L pooled RR | 2.119 | 0.899 | 4.992 |
|  |  |  |  |
| Overall |  |  |  |
| D+L pooled RR | **1.885** | **1.368** | **2.596** |

#### Diabetes

| Study | RR | [95% Conf. Interval] | |
| --- | --- | --- | --- |
| Severe clinical course of disease |  |  |  |
| Chen Q. et al. | 1.819 | 1.005 | 3.292 |
| Chen, G. et al. | 1.333 | 0.529 | 3.359 |
| Deng Q. et al. | 1.293 | 0.937 | 1.783 |
| Feng, Y. et al. | 1.780 | 1.202 | 2.638 |
| Guan, W. et al. | 2.418 | 1.851 | 3.160 |
| Li K. et al. | 3.902 | 2.524 | 6.032 |
| Li X. et al. | 1.343 | 1.107 | 1.627 |
| Li, Y. et al. | 2.206 | 0.837 | 5.812 |
| Liu, J. et al. | 2.519 | 1.136 | 5.584 |
| Liu, J. et al. | 2.400 | 1.029 | 5.600 |
| Liu, W. et al. | 3.244 | 0.943 | 11.162 |
| Mao, L. et al. | 1.260 | 0.845 | 1.880 |
| Qin, C. et al. | 1.143 | 0.969 | 1.350 |
| Shi, Y. et al. | 2.632 | 1.298 | 5.336 |
| Wan, S. et al. | 2.976 | 1.904 | 4.651 |
| Wang, X. et al. | 2.746 | 1.410 | 5.346 |
| Wei X. et al. | 1.009 | 0.684 | 1.488 |
| Wei YY. et al. | 4.316 | 2.405 | 7.746 |
| Xie, H. et al. | 0.683 | 0.198 | 2.356 |
| Yao Q. et al. | 3.745 | 1.117 | 12.562 |
| Yu X. et al. | 2.306 | 1.301 | 4.085 |
| Zhang G. et al | 1.319 | 0.682 | 2.551 |
| Zhang Y. et al. | 1.578 | 0.695 | 3.580 |
| Zhang, J. et al. | 1.158 | 0.670 | 2.002 |
| Zhang, J. et al. | 2.250 | 0.904 | 5.603 |
| Zhao X. et al. | 1.011 | 0.199 | 5.151 |
| Zheng S. et al. | 1.207 | 0.966 | 1.509 |
| Zheng, F. et al. | 1.571 | 0.465 | 5.314 |
| Duanmu Y. et al. | 1.286 | 0.464 | 3.559 |
| CDC COVID-19 Respons | 2.598 | 2.301 | 2.933 |
| Sub-total |  |  |  |
| D+L pooled RR | **1.829** | **1.526** | **2.192** |
|  |  |  |  |
| ICU |  |  |  |
| Du, R. et al. | 1.203 | 0.801 | 1.808 |
| Guan, W. et al. | 4.617 | 2.827 | 7.540 |
| Huang, C. et al. | 0.344 | 0.052 | 2.271 |
| Lei, S. et al. | 2.167 | 1.117 | 4.203 |
| Liu, Y. et al. | 2.117 | 1.593 | 2.814 |
| Wang, D. et al. | 2.531 | 1.448 | 4.424 |
| Wu, C. et al. | 1.914 | 1.394 | 2.629 |
| Yan Y. et al. | 1.611 | 1.220 | 2.128 |
| Simonnet A. et al. | 1.272 | 1.013 | 1.597 |
| Goyal, P. et al. | 1.137 | 0.835 | 1.550 |
| Kalligeros M. et al. | 1.562 | 1.010 | 2.414 |
| CDC COVID-19 Respons | 3.896 | 3.250 | 4.671 |
| Sub-total |  |  |  |
| D+L pooled RR | **1.881** | **1.375** | **2.574** |
|  |  |  |  |
| Death |  |  |  |
| Cao, J. et al. | 2.364 | 0.944 | 5.921 |
| Chen, T. et al. | 1.302 | 0.943 | 1.800 |
| Deng G. et al. | 4.427 | 3.493 | 5.610 |
| Guan, W. et al. | 3.946 | 2.153 | 7.233 |
| Li, Y. et al. | 4.167 | 1.309 | 13.261 |
| The Novel Coronaviru | 3.354 | 2.691 | 4.181 |
| Wang Dawei et al. | 3.117 | 1.389 | 6.995 |
| Wang, L. et al. | 1.075 | 0.602 | 1.919 |
| Wang, Y. et al. | 1.274 | 0.941 | 1.725 |
| Wu, C. et al. | 2.712 | 1.614 | 4.558 |
| Yan Y. et al. | 1.707 | 1.373 | 2.124 |
| Yang, X. et al. | 1.338 | 0.869 | 2.060 |
| Yao Q. et al. | 1.873 | 0.297 | 11.790 |
| Yuan, M. et al. | 4.540 | 1.942 | 10.614 |
| Zhang Y. et al. | 3.944 | 0.396 | 39.268 |
| Zhou, F. et al. | 1.978 | 1.267 | 3.088 |
| Nikpouraghdam M. et | 1.217 | 0.685 | 2.163 |
| Tomlins J. et al. | 2.911 | 1.281 | 6.617 |
| Sub-total |  |  |  |
| D+L pooled RR | **2.213** | **1.693** | **2.893** |
|  |  |  |  |
| Composite Endpoint |  |  |  |
| Deng Q. et al. | 1.428 | 0.722 | 2.824 |
| Sub-total |  |  |  |
| D+L pooled RR | 1.428 | 0.722 | 2.824 |
|  |  |  |  |
| Overall |  |  |  |
| D+L pooled RR | **1.938** | **1.697** | **2.213** |

#### Diseases of liver

| Study | RR | [95% Conf. Interval] | |
| --- | --- | --- | --- |
| Severe clinical course of disease |  |  |  |
| Chen Q. et al. | 2.376 | 1.271 | 4.440 |
| Guan, W. et al. | 2.049 | 1.182 | 3.553 |
| Ji Dong. et al. | 11.274 | 4.609 | 27.578 |
| Li X. et al. | 0.813 | 0.277 | 2.388 |
| Shi, Y. et al. | 0.899 | 0.233 | 3.466 |
| Wan, S. et al. | 1.705 | 0.416 | 6.990 |
| Yu X. et al. | 0.759 | 0.135 | 4.251 |
| Zhang, J. et al. | 1.222 | 0.593 | 2.518 |
| Zheng S. et al. | 0.861 | 0.384 | 1.931 |
| Zhu, Z. et al. | 2.449 | 0.687 | 8.725 |
| Sub-total |  |  |  |
| D+L pooled RR | **1.734** | **1.029** | **2.921** |
|  |  |  |  |
| Death |  |  |  |
| Guan, W. et al. | 1.138 | 0.163 | 7.956 |
| Wang Dawei et al. | 0.935 | 0.149 | 5.874 |
| Yao Q. et al. | 4.818 | 1.081 | 21.476 |
| Sub-total |  |  |  |
| D+L pooled RR | 1.930 | 0.580 | 6.418 |
|  |  |  |  |
| Overall |  |  |  |
| D+L pooled RR | **1.767** | **1.118** | **2.791** |

Diseases of the digestive system

| Study | RR | [95% Conf. Interval] | |
| --- | --- | --- | --- |
| Severe clinical course of disease |  |  |  |
| Wei YY. et al. | 1.951 | 0.728 | 5.227 |
| Zhang Jixiang. et al | 1.159 | 0.918 | 1.464 |
| Zhang, J. et al. | 0.679 | 0.207 | 2.226 |
| Sub-total |  |  |  |
| D+L pooled RR | 1.168 | 0.934 | 1.460 |
|  |  |  |  |
| ICU |  |  |  |
| Du, R. et al. | 0.775 | 0.398 | 1.509 |
| Sub-total |  |  |  |
| D+L pooled RR | 0.775 | 0.398 | 1.509 |
|  |  |  |  |
| Death |  |  |  |
| Zhang Jixiang. et al | 0.849 | 0.119 | 6.077 |
| Sub-total |  |  |  |
| D+L pooled RR | 0.849 | 0.119 | 6.077 |
|  |  |  |  |
| Overall |  |  |  |
| D+L pooled RR | 1.117 | 0.905 | 1.379 |

#### Endocrine diseases

| Study | RR | [95% Conf. Interval] | |
| --- | --- | --- | --- |
| Severe clinical course of disease |  |  |  |
| Zhang Jixiang. et al | 1.353 | 1.182 | 1.548 |
| Sub-total |  |  |  |
| D+L pooled RR | **1.353** | **1.182** | **1.548** |
|  |  |  |  |
| Death |  |  |  |
| Zhang Jixiang. et al | 1.213 | 0.373 | 3.946 |
| Sub-total |  |  |  |
| D+L pooled RR | 1.213 | 0.373 | 3.946 |
|  |  |  |  |
| Overall |  |  |  |
| D+L pooled RR | **1.351** | **1.182** | **1.545** |

#### Hypertension

| Study | RR | [95% Conf. Interval] | |
| --- | --- | --- | --- |
| Severe clinical course of disease |  |  |  |
| Chen Q. et al. | 1.480 | 0.830 | 2.638 |
| Chen, G. et al. | 1.829 | 0.901 | 3.711 |
| Deng Q. et al. | 1.178 | 0.870 | 1.596 |
| Feng, Y. et al. | 1.530 | 1.120 | 2.089 |
| Guan, W. et al. | 2.603 | 2.084 | 3.253 |
| Li K. et al. | 1.357 | 0.440 | 4.187 |
| Li X. et al. | 1.450 | 1.231 | 1.710 |
| Li, Y. et al. | 1.438 | 0.322 | 6.408 |
| Liu, J. et al. | 3.542 | 1.752 | 7.159 |
| Liu, J. et al. | 2.227 | 1.033 | 4.804 |
| Liu, W. et al. | 1.944 | 0.506 | 7.473 |
| Mao, L. et al. | 1.826 | 1.354 | 2.464 |
| Qin, C. et al. | 1.362 | 1.195 | 1.553 |
| Shi, Y. et al. | 4.430 | 2.645 | 7.421 |
| Wan, S. et al. | 1.043 | 0.441 | 2.465 |
| Wang, X. et al. | 1.340 | 0.620 | 2.896 |
| Wei X. et al. | 1.437 | 1.120 | 1.842 |
| Xie, H. et al. | 0.774 | 0.319 | 1.879 |
| Yao Q. et al. | 1.045 | 0.255 | 4.282 |
| Yu X. et al. | 2.043 | 1.147 | 3.639 |
| Zhang G. et al | 2.773 | 1.801 | 4.268 |
| Zhang Y. et al. | 2.810 | 1.502 | 5.256 |
| Zhang, J. et al. | 1.426 | 0.967 | 2.102 |
| Zhang, J. et al. | 2.182 | 0.585 | 8.134 |
| Zhao X. et al. | 1.738 | 0.967 | 3.124 |
| Zheng S. et al. | 1.256 | 1.027 | 1.537 |
| Zheng, F. et al. | 4.212 | 2.369 | 7.490 |
| Zhu, Z. et al. | 3.097 | 1.269 | 7.559 |
| Duanmu Y. et al. | 2.132 | 1.073 | 4.233 |
| Sub-total |  |  |  |
| D+L pooled RR | **1.799** | **1.557** | **2.078** |
|  |  |  |  |
| ICU |  |  |  |
| Du, R. et al. | 0.892 | 0.598 | 1.332 |
| Huang, C. et al. | 1.061 | 0.309 | 3.639 |
| Lei, S. et al. | 2.423 | 1.125 | 5.219 |
| Liu, Y. et al. | 1.277 | 0.872 | 1.871 |
| Wang, D. et al. | 3.093 | 1.774 | 5.394 |
| Wu, C. et al. | 1.566 | 1.128 | 2.175 |
| Simonnet A. et al. | 1.384 | 1.084 | 1.767 |
| Goyal, P. et al. | 1.161 | 0.875 | 1.540 |
| Kalligeros M. et al. | 1.337 | 0.805 | 2.220 |
| Sub-total |  |  |  |
| D+L pooled RR | **1.404** | **1.144** | **1.723** |
|  |  |  |  |
| Death |  |  |  |
| Cao, J. et al. | 3.304 | 1.452 | 7.514 |
| Chen, T. et al. | 1.781 | 1.358 | 2.337 |
| Deng G. et al. | 4.482 | 3.689 | 5.445 |
| Fan J. et al. | 1.100 | 0.189 | 6.413 |
| Guan, W. et al. | 6.250 | 3.632 | 10.755 |
| Li, Y. et al. | 2.875 | 0.554 | 14.932 |
| The Novel Coronaviru | 2.923 | 2.482 | 3.443 |
| Wang Dawei et al. | 3.462 | 1.579 | 7.587 |
| Wang, L. et al. | 1.412 | 0.914 | 2.183 |
| Wang, Y. et al. | 1.552 | 1.192 | 2.021 |
| Wu, C. et al. | 2.374 | 1.432 | 3.933 |
| Yao Q. et al. | 8.050 | 2.910 | 22.268 |
| Yuan, M. et al. | 3.833 | 1.779 | 8.262 |
| Zhang Y. et al. | 5.619 | 0.537 | 58.814 |
| Zhou, F. et al. | 2.129 | 1.377 | 3.292 |
| Nikpouraghdam M. et | 1.705 | 0.885 | 3.286 |
| Tomlins J. et al. | 2.095 | 0.964 | 4.553 |
| Sub-total |  |  |  |
| D+L pooled RR | **2.659** | **2.061** | **3.430** |
|  |  |  |  |
| Composite Endpoint |  |  |  |
| Deng Q. et al. | 1.333 | 0.729 | 2.440 |
| Guan, W. et al. | 3.159 | 1.972 | 5.061 |
| Sub-total |  |  |  |
| D+L pooled RR | 2.097 | 0.901 | 4.879 |
|  |  |  |  |
| Overall |  |  |  |
| D+L pooled RR | **1.952** | **1.710** | **2.229** |

#### Immunocompromised condition

| Study | RR | [95% Conf. Interval] | |
| --- | --- | --- | --- |
| Severe clinical course of disease |  |  |  |
| CDC COVID-19 Response | 1.690 | 1.352 | 2.113 |
| Sub-total |  |  |  |
| D+L pooled RR | **1.690** | **1.352** | **2.113** |
|  |  |  |  |
| ICU |  |  |  |
| CDC COVID-19 Response | 2.575 | 1.915 | 3.463 |
| Sub-total |  |  |  |
| D+L pooled RR | **2.575** | **1.915** | **3.463** |
|  |  |  |  |
| Overall |  |  |  |
| D+L pooled RR | **2.062** | **1.365** | **3.114** |

#### Mycobacterial diseases

| Study | RR | [95% Conf. Interval] | |
| --- | --- | --- | --- |
| Severe clinical course of disease |  |  |  |
| Li X. et al. | 0.904 | 0.433 | 1.886 |
| Sub-total |  |  |  |
| D+L pooled RR | 0.904 | 0.433 | 1.886 |
|  |  |  |  |
| Overall |  |  |  |
| D+L pooled RR | 0.904 | 0.433 | 1.886 |

#### Other diseases or unspecified

| Study | RR | [95% Conf. Interval] | |
| --- | --- | --- | --- |
| Severe clinical course of disease |  |  |  |
| Ji D. et al. | 6.037 | 3.488 | 10.448 |
| Ji Dong. et al. | 5.277 | 3.025 | 9.205 |
| Ma J. et al. | 1.389 | 0.784 | 2.459 |
| Wang, X. et al. | 0.890 | 0.297 | 2.664 |
| Wei YY. et al. | 2.446 | 1.304 | 4.589 |
| Zou Y. et al | 4.197 | 1.820 | 9.676 |
| Sub-total |  |  |  |
| D+L pooled RR | **2.875** | **1.607** | **5.143** |
|  |  |  |  |
| ICU |  |  |  |
| Wang R. et al. | 2.471 | 1.253 | 4.870 |
| Sub-total |  |  |  |
| D+L pooled RR | **2.471** | **1.253** | **4.870** |
|  |  |  |  |
| Death |  |  |  |
| Ruan, Q. et al. | 1.631 | 1.121 | 2.372 |
| Tang, N. et al. | 1.920 | 0.852 | 4.327 |
| Sub-total |  |  |  |
| D+L pooled RR | **1.678** | **1.194** | **2.358** |
|  |  |  |  |
| Overall |  |  |  |
| D+L pooled RR | **2.531** | **1.673** | **3.829** |

#### Other respiratory diseases

| Study | RR | [95% Conf. Interval] | |
| --- | --- | --- | --- |
| Severe clinical course of disease |  |  |  |
| Wang, X. et al. | 1.012 | 0.268 | 3.820 |
| Wei YY. et al. | 2.911 | 1.033 | 8.205 |
| Zhang Jixiang. et al | 1.229 | 1.034 | 1.461 |
| Zhang, J. et al. | 0.930 | 0.503 | 1.722 |
| Zheng S. et al. | 1.187 | 0.867 | 1.625 |
| Zhu, Z. et al. | 2.881 | 0.839 | 9.897 |
| CDC COVID-19 Response | 1.703 | 1.463 | 1.983 |
| Sub-total |  |  |  |
| D+L pooled RR | **1.389** | **1.091** | **1.768** |
|  |  |  |  |
| ICU |  |  |  |
| Du, R. et al. | 0.461 | 0.191 | 1.110 |
| Kalligeros M. et al. | 1.383 | 0.859 | 2.228 |
| CDC COVID-19 Respons | 2.568 | 2.077 | 3.175 |
| Sub-total |  |  |  |
| D+L pooled RR | 1.304 | 0.578 | 2.940 |
|  |  |  |  |
| Death |  |  |  |
| Chen, T. et al. | 1.534 | 1.030 | 2.284 |
| Deng G. et al. | 3.428 | 2.415 | 4.865 |
| The Novel Coronaviru | 2.791 | 1.984 | 3.925 |
| Yang, X. et al. | 0.800 | 0.293 | 2.184 |
| Zhang Jixiang. et al | 3.000 | 1.175 | 7.660 |
| Cao, J. et al. | 1.840 | 0.641 | 5.279 |
| Nikpouraghdam M. et | 1.894 | 1.024 | 3.503 |
| Sub-total |  |  |  |
| D+L pooled RR | **2.166** | **1.546** | **3.035** |
|  |  |  |  |
| Overall |  |  |  |
| D+L pooled RR | **1.682** | **1.343** | **2.105** |

### Demographic, Occupational or lifestyle Factors

#### Healthcare workers

| Study | RR | [95% Conf. Interval] | |
| --- | --- | --- | --- |
| Severe clinical course of disease |  |  |  |
| Li X. et al. | 0.388 | 0.215 | 0.700 |
| Duanmu Y. et al. | 0.750 | 0.253 | 2.220 |
| McMichael, T. et al. | 0.111 | 0.037 | 0.338 |
| Sub-total |  |  |  |
| D+L pooled RR | **0.327** | **0.125** | **0.855** |
|  |  |  |  |
| Death |  |  |  |
| Chen, T. et al. | 0.120 | 0.018 | 0.811 |
| The Novel Coronavirus Team | 0.123 | 0.051 | 0.296 |
| Sub-total |  |  |  |
| D+L pooled RR | **0.122** | **0.055** | **0.272** |
|  |  |  |  |
| Overall |  |  |  |
| D+L pooled RR | **0.234** | **0.105** | **0.521** |

#### Male

| Study | RR | [95% Conf. Interval] | |
| --- | --- | --- | --- |
| Severe clinical course of disease |  |  |  |
| Cai, Q. et al. | 2.166 | 1.315 | 3.568 |
| Chen Q. et al. | 0.961 | 0.581 | 1.588 |
| Chen, G. et al. | 2.353 | 0.412 | 13.451 |
| Deng Q. et al. | 1.264 | 0.927 | 1.724 |
| Feng, Y. et al. | 1.425 | 1.032 | 1.967 |
| Ji D. et al. | 1.815 | 0.978 | 3.368 |
| Ji Dong. et al. | 1.772 | 0.953 | 3.296 |
| Li K. et al. | 1.330 | 0.678 | 2.609 |
| Li X. et al. | 1.272 | 1.069 | 1.513 |
| Li, Y. et al. | 2.167 | 0.691 | 6.791 |
| Liu, J. et al. | 1.944 | 0.804 | 4.701 |
| Liu, J. et al. | 1.382 | 0.606 | 3.155 |
| Ma J. et al. | 0.850 | 0.470 | 1.536 |
| Mao, L. et al. | 1.460 | 1.064 | 2.003 |
| Qin, C. et al. | 1.093 | 0.948 | 1.259 |
| Shi, Y. et al. | 2.438 | 1.326 | 4.481 |
| Sun S. et al | 3.524 | 1.741 | 7.131 |
| Tian, S. et al. | 1.382 | 0.813 | 2.348 |
| Wan, S. et al. | 0.967 | 0.575 | 1.627 |
| Wang, X. et al. | 1.519 | 1.034 | 2.232 |
| Wei X. et al. | 1.333 | 1.023 | 1.736 |
| Wei YY. et al. | 1.516 | 0.757 | 3.036 |
| Xie, H. et al. | 1.432 | 0.760 | 2.696 |
| Xu, Y. et al. | 0.845 | 0.332 | 2.151 |
| Yao Q. et al. | 1.296 | 0.467 | 3.594 |
| Yu X. et al. | 2.018 | 0.968 | 4.203 |
| Zhang G. et al | 1.831 | 1.131 | 2.965 |
| Zhang Jixiang. et al | 1.071 | 0.950 | 1.207 |
| Zhang Y. et al. | 0.855 | 0.426 | 1.714 |
| Zhang, J. et al. | 1.283 | 0.860 | 1.914 |
| Zhang, J. et al. | 1.212 | 0.402 | 3.657 |
| Zhao X. et al. | 0.750 | 0.417 | 1.349 |
| Zheng S. et al. | 1.284 | 0.996 | 1.656 |
| Zheng, F. et al. | 0.886 | 0.464 | 1.693 |
| Zhu, Z. et al. | 2.343 | 0.935 | 5.869 |
| Zou Y. et al | 3.059 | 1.264 | 7.405 |
| Sub-total |  |  |  |
| D+L pooled RR | **1.339** | **1.216** | **1.474** |
|  |  |  |  |
| ICU |  |  |  |
| Du, R. et al. | 1.135 | 0.725 | 1.778 |
| Guan, W. et al. | 1.474 | 0.898 | 2.419 |
| Huang, C. et al. | 2.017 | 0.529 | 7.693 |
| Lei, S. et al. | 0.714 | 0.312 | 1.635 |
| Liu, Y. et al. | 0.949 | 0.645 | 1.396 |
| Wang R. et al. | 1.352 | 0.648 | 2.823 |
| Wang, D. et al. | 1.320 | 0.739 | 2.358 |
| Wu, C. et al. | 1.426 | 0.979 | 2.077 |
| Simonnet A. et al. | 1.151 | 0.857 | 1.547 |
| Goyal, P. et al. | 1.577 | 1.146 | 2.170 |
| Kalligeros M. et al. | 1.228 | 0.759 | 1.986 |
| Sub-total |  |  |  |
| D+L pooled RR | **1.257** | **1.098** | **1.437** |
|  |  |  |  |
| Death |  |  |  |
| Cao, J. et al. | 1.849 | 0.752 | 4.547 |
| Chen, T. et al. | 1.666 | 1.188 | 2.338 |
| Deng G. et al. | 1.646 | 1.451 | 1.867 |
| Fan J. et al. | 0.909 | 0.156 | 5.300 |
| Li, Y. et al. | 4.333 | 0.560 | 33.527 |
| Tang, N. et al. | 2.776 | 1.061 | 7.258 |
| The Novel Coronaviru | 1.666 | 1.468 | 1.890 |
| Wang Dawei et al. | 4.678 | 1.447 | 15.122 |
| Wang, L. et al. | 1.563 | 0.999 | 2.447 |
| Wang, Y. et al. | 1.156 | 0.884 | 1.513 |
| Wu, C. et al. | 1.103 | 0.634 | 1.917 |
| Yang, X. et al. | 0.927 | 0.595 | 1.444 |
| Yao Q. et al. | 2.116 | 0.718 | 6.238 |
| Yuan, M. et al. | 0.833 | 0.303 | 2.293 |
| Zhang Jixiang. et al | 1.598 | 0.729 | 3.506 |
| Zhang Y. et al. | 0.712 | 0.067 | 7.533 |
| Zhou, F. et al. | 1.437 | 0.867 | 2.382 |
| Nikpouraghdam M. et | 1.197 | 0.918 | 1.561 |
| Tomlins J. et al. | 0.875 | 0.397 | 1.931 |
| Sub-total |  |  |  |
| D+L pooled RR | **1.443** | **1.274** | **1.634** |
|  |  |  |  |
| Composite Endpoint |  |  |  |
| Deng Q. et al. | 1.528 | 0.821 | 2.842 |
| Liu, W. et al. | 1.750 | 0.557 | 5.502 |
| Sub-total |  |  |  |
| D+L pooled RR | 1.576 | 0.913 | 2.719 |
|  |  |  |  |
| Overall |  |  |  |
| D+L pooled RR | **1.354** | **1.259** | **1.457** |

#### Obesity

| Study | RR | [95% Conf. Interval] | |
| --- | --- | --- | --- |
| Severe clinical course of disease |  |  |  |
| Zheng KI. et al. | 3.967 | 1.007 | 15.619 |
| Duanmu Y. et al. | 2.127 | 1.080 | 4.189 |
| Sub-total |  |  |  |
| D+L pooled RR | **2.404** | **1.310** | **4.413** |
|  |  |  |  |
| ICU |  |  |  |
| Goyal, P. et al. | 1.376 | 1.042 | 1.817 |
| Kalligeros M. et al. | 1.450 | 0.921 | 2.284 |
| Sub-total |  |  |  |
| D+L pooled RR | **1.396** | **1.101** | **1.769** |
|  |  |  |  |
| Overall |  |  |  |
| D+L pooled RR | **1.532** | **1.187** | **1.977** |

#### Smoking

| Study | RR | [95% Conf. Interval] | |
| --- | --- | --- | --- |
| Severe clinical course of disease |  |  |  |
| Chen Q. et al. | 0.650 | 0.229 | 1.847 |
| Feng, Y. et al. | 1.523 | 1.013 | 2.290 |
| Ji D. et al. | 1.755 | 0.847 | 3.637 |
| Ji Dong. et al. | 1.101 | 0.439 | 2.763 |
| Li X. et al. | 1.171 | 0.952 | 1.441 |
| Li, Y. et al. | 3.214 | 1.203 | 8.591 |
| Liu, J. et al. | 1.273 | 0.391 | 4.141 |
| Liu, J. et al. | 1.900 | 0.650 | 5.550 |
| Liu, W. et al. | 5.475 | 2.077 | 14.434 |
| Qin, C. et al. | 0.674 | 0.286 | 1.590 |
| Shi, Y. et al. | 1.559 | 0.707 | 3.438 |
| Wan, S. et al. | 0.359 | 0.056 | 2.320 |
| Yu X. et al. | 1.188 | 0.582 | 2.425 |
| Zhang, J. et al. | 1.078 | 0.505 | 2.302 |
| Zou Y. et al | 1.720 | 0.451 | 6.564 |
| Duanmu Y. et al. | 3.667 | 1.967 | 6.836 |
| CDC COVID-19 Response | 1.826 | 1.474 | 2.263 |
| Sub-total |  |  |  |
| D+L pooled RR | **1.537** | **1.213** | **1.948** |
|  |  |  |  |
| ICU |  |  |  |
| Guan, W. et al. | 2.934 | 1.809 | 4.756 |
| Wang R. et al. | 2.649 | 1.319 | 5.323 |
| Goyal, P. et al. | 0.902 | 0.455 | 1.790 |
| Kalligeros M. et al. | 0.955 | 0.609 | 1.497 |
| CDC COVID-19 Response | 2.398 | 1.762 | 3.264 |
| Sub-total |  |  |  |
| D+L pooled RR | **1.765** | **1.083** | **2.878** |
|  |  |  |  |
| Death |  |  |  |
| Chen, T. et al. | 1.177 | 0.813 | 1.703 |
| Li, Y. et al. | 3.857 | 0.809 | 18.387 |
| Yao Q. et al. | 8.667 | 3.732 | 20.128 |
| Zhou, F. et al. | 1.670 | 0.837 | 3.329 |
| Sub-total |  |  |  |
| D+L pooled RR | **2.634** | **1.016** | **6.826** |
|  |  |  |  |
| Overall |  |  |  |
| D+L pooled RR | **1.685** | **1.369** | **2.074** |

## Meta-analysis of comorbidities and severe clinical course of disease

| **Comorbidity** | **Study** | **RR** | **[95% Conf.**  **Interval]** | |
| --- | --- | --- | --- | --- |
| Acute renal failure |  |  |  |  |
|  | Xiao, G. et al. | 1.8 | 1.2 | 2.6 |
|  | Xiao, G. et al. | 1.6 | 1.2 | 2.1 |
|  | D+L pooled | 1.7 | 1.3 | 2.1 |
| Asthma |  |  |  |  |
|  | Li X. et al. | 1.2 | 0.6 | 2.5 |
|  | Duanmu Y. et al. | 1.3 | 0.5 | 3.6 |
|  | D+L pooled | 1.2 | 0.7 | 2.2 |
| Cancer |  |  |  |  |
|  | Feng, Y. et al. | 2.3 | 1.4 | 3.8 |
|  | Li X. et al. | 1.2 | 0.8 | 1.7 |
|  | Liu, Jing. et al. | 0.8 | 0.1 | 4.4 |
|  | Mao, L. et al. | 0.9 | 0.5 | 1.9 |
|  | Qin, C. et al. | 1.1 | 0.8 | 1.6 |
|  | Wan, S. et al. | 2.7 | 1.4 | 5.0 |
|  | Yao Q. et al. | 4.4 | 1.0 | 19.5 |
|  | Zhang Jixiang. | 1.3 | 1.0 | 1.7 |
|  | Zhang Y. et al. | 1.7 | 0.6 | 4.9 |
|  | Zhao X. et al. | 2.1 | 0.9 | 4.9 |
|  | Zhu, Z. et al. | 1.6 | 0.3 | 10.0 |
|  | Duanmu Y. et al. | 1.4 | 0.3 | 7.2 |
|  | D+L pooled | 1.5 | 1.2 | 1.8 |
| Cardiovascular Disease |  |  |  |  |
|  | Feng, Y. et al. | 1.8 | 1.2 | 2.7 |
|  | Guan, W. et al. | 2.2 | 1.5 | 3.2 |
|  | Li, Y. et al. | 0.7 | 0.1 | 3.9 |
|  | Liu, J. et al. | 2.8 | 1.1 | 6.8 |
|  | Qin, C. et al. | 1.4 | 1.2 | 1.7 |
|  | Shi, Y. et al. | 3.8 | 1.7 | 8.8 |
|  | Wan, S. et al. | 3.2 | 2.1 | 4.9 |
|  | Wang, X. et al. | 3.5 | 1.7 | 7.3 |
|  | Wei X. et al. | 1.6 | 1.2 | 2.2 |
|  | Wei YY. et al. | 1.8 | 0.9 | 3.8 |
|  | Yu X. et al. | 2.1 | 1.1 | 3.9 |
|  | Zhang G. et al. | 2.8 | 1.8 | 4.3 |
|  | Zhang Jixiang. et al. | 1.4 | 1.3 | 1.6 |
|  | Zhu, Z. et al. | 2.9 | 0.8 | 9.9 |
|  | Deng Q. et al. | 1.3 | 0.9 | 1.8 |
|  | Li K. et al. | 2.5 | 1.1 | 6.0 |
|  | Li X. et al. | 1.8 | 1.5 | 2.1 |
|  | Xie, H. et al. | 0.8 | 0.2 | 2.7 |
|  | Zhang, J. et al. | 1.4 | 0.7 | 2.8 |
|  | Zhang, J. et al. | 2.7 | 1.2 | 5.9 |
|  | Zheng S. et al. | 1.3 | 1.0 | 1.6 |
|  | Zheng, F. et al. | 2.8 | 1.0 | 7.9 |
|  | CDC COVID-19 Team | 3.1 | 2.7 | 3.5 |
|  | D+L pooled | 2.0 | 1.6 | 2.4 |
| Cerebrovascular Disease |  |  |  |  |
|  | Mao, L. et al. | 1.1 | 0.7 | 2.0 |
|  | Feng, Y. et al. | 2.1 | 1.3 | 3.4 |
|  | Guan, W. et al. | 3.3 | 2.2 | 4.8 |
|  | Zhang G. et al. | 3.4 | 2.3 | 5.1 |
|  | Zhang, J. et al. | 1.8 | 0.6 | 4.9 |
|  | Zheng, F. et al. | 1.4 | 0.2 | 7.6 |
|  | D+L pooled | 2.2 | 1.5 | 3.3 |
| Chronic Obstructive Pulmonary Disease |  |  |  |  |
|  | Deng Q. et al. | 1.3 | 0.7 | 2.3 |
|  | Li K. et al. | 3.0 | 1.7 | 5.3 |
|  | Li X. et al. | 1.6 | 1.2 | 2.1 |
|  | Li, Y. et al. | 3.2 | 1.3 | 7.7 |
|  | Liu, J. et al. | 2.4 | 1.0 | 5.6 |
|  | Liu, W. et al. | 3.8 | 0.8 | 17.1 |
|  | Qin, C. et al. | 1.2 | 0.9 | 1.7 |
|  | Zhang G. et al. | 2.8 | 1.5 | 5.2 |
|  | Zhao X. et al. | 2.3 | 1.0 | 5.4 |
|  | Zheng, F. et al. | 1.8 | 0.6 | 6.0 |
|  | Duanmu Y. et al. | 3.2 | 1.3 | 7.7 |
|  | D+L pooled | 2.0 | 1.5 | 2.6 |
| Chronic renal failure |  |  |  |  |
|  | Chen Q. et al. | 1.1 | 0.2 | 5.7 |
|  | Cheng, Y. et al. | 1.3 | 1.0 | 1.6 |
|  | Li X. et al. | 1.2 | 0.7 | 2.0 |
|  | Mao, L. et al. | 0.8 | 0.3 | 2.5 |
|  | Shi, Y. et al. | 2.9 | 0.9 | 9.7 |
|  | Zhang Jixiang. | 1.2 | 1.0 | 1.6 |
|  | Zhao X. et al. | 2.3 | 1.0 | 5.4 |
|  | CDC COVID-19 Team | 3.3 | 2.8 | 3.9 |
|  | D+L pooled | 1.6 | 1.0 | 2.6 |
| Diabetes mellitus |  |  |  |  |
|  | Chen Q. et al. | 1.8 | 1.0 | 3.3 |
|  | Chen, G. et al. | 1.3 | 0.5 | 3.4 |
|  | Deng Q. et al. | 1.3 | 0.9 | 1.8 |
|  | Feng, Y. et al. | 1.8 | 1.2 | 2.6 |
|  | Guan, W. et al. | 2.4 | 1.9 | 3.2 |
|  | Li K. et al. | 3.9 | 2.5 | 6.0 |
|  | Li X. et al. | 1.3 | 1.1 | 1.6 |
|  | Li, Y. et al. | 2.2 | 0.8 | 5.8 |
|  | Liu, J. et al. | 2.5 | 1.1 | 5.6 |
|  | Liu, J. et al. | 2.4 | 1.0 | 5.6 |
|  | Liu, W. et al. | 3.2 | 0.9 | 11.2 |
|  | Mao, L. et al. | 1.3 | 0.8 | 1.9 |
|  | Qin, C. et al. | 1.1 | 1.0 | 1.4 |
|  | Shi, Y. et al. | 2.6 | 1.3 | 5.3 |
|  | Wan, S. et al. | 3.0 | 1.9 | 4.7 |
|  | Wang, X. et al. | 2.7 | 1.4 | 5.3 |
|  | Wei X. et al. | 1.0 | 0.7 | 1.5 |
|  | Wei YY. et al. | 4.3 | 2.4 | 7.7 |
|  | Xie, H. et al. | 0.7 | 0.2 | 2.4 |
|  | Yao Q. et al. | 3.7 | 1.1 | 12.6 |
|  | Yu X. et al. | 2.3 | 1.3 | 4.1 |
|  | Zhang G. et al | 1.3 | 0.7 | 2.6 |
|  | Zhang Y. et al. | 1.6 | 0.7 | 3.6 |
|  | Zhang, J. et al. | 1.2 | 0.7 | 2.0 |
|  | Zhang, J. et al. | 2.3 | 0.9 | 5.6 |
|  | Zhao X. et al. | 1.0 | 0.2 | 5.2 |
|  | Zheng S. et al. | 1.2 | 1.0 | 1.5 |
|  | Zheng, F. et al. | 1.6 | 0.5 | 5.3 |
|  | Duanmu Y. et al. | 1.3 | 0.5 | 3.6 |
|  | CDC COVID-19 Team | 2.6 | 2.3 | 2.9 |
|  | D+L pooled RR | 1.8 | 1.5 | 2.2 |
| Diseases of liver |  |  |  |  |
|  | Chen Q. et al. | 2.4 | 1.3 | 4.4 |
|  | Guan, W. et al. | 2.0 | 1.2 | 3.6 |
|  | Ji Dong. et al. | 11.3 | 4.6 | 27.6 |
|  | Li X. et al. | 0.8 | 0.3 | 2.4 |
|  | Shi, Y. et al. | 0.9 | 0.2 | 3.5 |
|  | Wan, S. et al. | 1.7 | 0.4 | 7.0 |
|  | Yu X. et al. | 0.8 | 0.1 | 4.3 |
|  | Zhang, J. et al. | 1.2 | 0.6 | 2.5 |
|  | Zheng S. et al. | 0.9 | 0.4 | 1.9 |
|  | Zhu, Z. et al. | 2.4 | 0.7 | 8.7 |
|  | D+L pooled | 1.7 | 1.0 | 2.9 |
| Diseases of the digestive system |  |  |  |  |
|  | Wei YY. et al. | 2.0 | 0.7 | 5.2 |
|  | Zhang Jixiang. et al. | 1.2 | 0.9 | 1.5 |
|  | Zhang, J. et al. | 0.7 | 0.2 | 2.2 |
|  | D+L pooled | 1.2 | 0.9 | 1.5 |
| Endocrine disease |  |  |  |  |
|  | Zhang Jixiang. | 1.4 | 1.2 | 1.5 |
|  | D+L pooled R | 1.4 | 1.2 | 1.5 |
| Hypertension |  |  |  |  |
|  | Chen Q. et al. | 1.5 | 0.8 | 2.6 |
|  | Chen, G. et al. | 1.8 | 0.9 | 3.7 |
|  | Deng Q. et al. | 1.2 | 0.9 | 1.6 |
|  | Feng, Y. et al. | 1.5 | 1.1 | 2.1 |
|  | Guan, W. et al. | 2.6 | 2.1 | 3.3 |
|  | Li K. et al. | 1.4 | 0.4 | 4.2 |
|  | Li X. et al. | 1.5 | 1.2 | 1.7 |
|  | Li, Y. et al. | 1.4 | 0.3 | 6.4 |
|  | Liu, J. et al. | 3.5 | 1.8 | 7.2 |
|  | Liu, J. et al. | 2.2 | 1.0 | 4.8 |
|  | Liu, W. et al. | 1.9 | 0.5 | 7.5 |
|  | Mao, L. et al. | 1.8 | 1.4 | 2.5 |
|  | Qin, C. et al. | 1.4 | 1.2 | 1.6 |
|  | Shi, Y. et al. | 4.4 | 2.6 | 7.4 |
|  | Wan, S. et al. | 1.0 | 0.4 | 2.5 |
|  | Wang, X. et al. | 1.3 | 0.6 | 2.9 |
|  | Wei X. et al. | 1.4 | 1.1 | 1.8 |
|  | Xie, H. et al. | 0.8 | 0.3 | 1.9 |
|  | Yao Q. et al. | 1.0 | 0.3 | 4.3 |
|  | Yu X. et al. | 2.0 | 1.1 | 3.6 |
|  | Zhang G. et al. | 2.8 | 1.8 | 4.3 |
|  | Zhang Y. et al. | 2.8 | 1.5 | 5.3 |
|  | Zhang, J. et al. | 1.4 | 1.0 | 2.1 |
|  | Zhang, J. et al. | 2.2 | 0.6 | 8.1 |
|  | Zhao X. et al. | 1.7 | 1.0 | 3.1 |
|  | Zheng S. et al. | 1.3 | 1.0 | 1.5 |
|  | Zheng, F. et al. | 4.2 | 2.4 | 7.5 |
|  | Zhu, Z. et al. | 3.1 | 1.3 | 7.6 |
|  | Duanmu Y. et al. | 2.1 | 1.1 | 4.2 |
|  | D+L pooled | 1.8 | 1.6 | 2.1 |
| Immunocompromised condition |  |  |  |  |
|  | CDC COVID-19 Team | 1.7 | 1.4 | 2.1 |
|  | D+L pooled | 1.7 | 1.4 | 2.1 |
| Mycobacterial disease |  |  |  |  |
|  | Li X. et al. | 0.9 | 0.4 | 1.9 |
|  | D+L pooled | 0.9 | 0.4 | 1.9 |
| Other diseases or unspecified disease |  |  |  |  |
|  | Ji D. et al. | 6.0 | 3.5 | 10.4 |
|  | Ji Dong. et al. | 5.3 | 3.0 | 9.2 |
|  | Ma J. et al. | 1.4 | 0.8 | 2.5 |
|  | Wang, X. et al. | 0.9 | 0.3 | 2.7 |
|  | Wei YY. et al. | 2.4 | 1.3 | 4.6 |
|  | Zou Y. et al. | 4.2 | 1.8 | 9.7 |
|  | D+L pooled | 2.9 | 1.6 | 5.1 |
| Other respiratory diseases |  |  |  |  |
|  | Wang, X. et al. | 1.0 | 0.3 | 3.8 |
|  | Wei YY. et al. | 2.9 | 1.0 | 8.2 |
|  | Zhang Jixiang. | 1.2 | 1.0 | 1.5 |
|  | Zhang, J. et al. | 0.9 | 0.5 | 1.7 |
|  | Zheng S. et al. | 1.2 | 0.9 | 1.6 |
|  | Zhu, Z. et al. | 2.9 | 0.8 | 9.9 |
|  | CDC COVID-19 Team | 1.7 | 1.5 | 2.0 |
|  | D+L pooled | 1.4 | 1.1 | 1.8 |

## Meta-analysis of Comorbidities and ICU admission

| **Comorbidity** | **Study** | **RR** | **[95% Conf.**  **Interval]** | |
| --- | --- | --- | --- | --- |
| Asthma |  |  |  |  |
|  | Goyal, P. et al. | 1.1 | 0.7 | 1.6 |
|  | D+L pooled | 1.1 | 0.7 | 1.6 |
| Cancer |  |  |  |  |
|  | Lei, S. et al. | 1.4 | 0.7 | 3.0 |
|  | Liang, W. et al. | 4.9 | 2.7 | 9.0 |
|  | Liu, W. et al. | 4.1 | 1.3 | 13.1 |
|  | Wang, D. et al. | 1.6 | 0.7 | 3.6 |
|  | Kalligeros M. et al. | 1.6 | 1.0 | 2.8 |
|  | D+L pooled | 2.3 | 1.3 | 4.0 |
| Cardiovascular Disease |  |  |  |  |
|  | Huang, C. et al. | 1.8 | 0.7 | 4.6 |
|  | Lei, S. et al. | 2.6 | 1.4 | 4.7 |
|  | Liu, Y. et al. | 0.9 | 0.4 | 2.1 |
|  | Wang, D. et al. | 2.0 | 1.1 | 3.5 |
|  | Guan, W. et al. | 3.9 | 1.9 | 8.2 |
|  | Goyal, P. et al. | 1.5 | 1.1 | 2.1 |
|  | Kalligeros M. et al. | 1.5 | 0.9 | 2.3 |
|  | CDC COVID-19 Team | 4.1 | 3.4 | 4.9 |
|  | D+L pooled | 2.1 | 1.3 | 3.2 |
| Cerebrovascular Disease |  |  |  |  |
|  | Du, R. et al. | 0.8 | 0.5 | 1.3 |
|  | Lei, S. et al. | 2.0 | 1.1 | 3.9 |
|  | Liu, Y. et al. | 2.0 | 1.5 | 2.7 |
|  | Wang, D. et al. | 3.7 | 2.4 | 5.8 |
|  | D+L pooled | 1.9 | 0.9 | 4.0 |
| Chronic obstructive pulmonary disease |  |  |  |  |
|  | Guan, W. et al. | 10.6 | 6.2 | 18.1 |
|  | Huang, C. et al. | 2.5 | 1.0 | 6.2 |
|  | Liu, Y. et al. | 1.0 | 0.4 | 2.8 |
|  | Goyal, P. et al. | 1.1 | 0.6 | 2.0 |
|  | D+L pooled | 2.4 | 0.6 | 9.8 |
| Chronic renal failure |  |  |  |  |
|  | Cheng, Y. et al. | 1.3 | 0.7 | 2.3 |
|  | Duanmu Y. et al. | 3.1 | 1.6 | 6.2 |
|  | Kalligeros M. et al. | 0.8 | 0.4 | 1.9 |
|  | CDC COVID-19 Team | 4.6 | 3.6 | 5.8 |
|  | D+L pooled | 2.1 | 0.9 | 4.9 |
| Diabetes mellitus |  |  |  |  |
|  | Du, R. et al. | 1.2 | 0.8 | 1.8 |
|  | Guan, W. et al. | 4.6 | 2.8 | 7.5 |
|  | Huang, C. et al. | 0.3 | 0.1 | 2.3 |
|  | Lei, S. et al. | 2.2 | 1.1 | 4.2 |
|  | Liu, Y. et al. | 2.1 | 1.6 | 2.8 |
|  | Wang, D. et al. | 2.5 | 1.4 | 4.4 |
|  | Wu, C. et al. | 1.9 | 1.4 | 2.6 |
|  | Yan Y. et al. | 1.6 | 1.2 | 2.1 |
|  | Simonnet A. et al. | 1.3 | 1.0 | 1.6 |
|  | Goyal, P. et al. | 1.1 | 0.8 | 1.6 |
|  | Kalligeros M. et al. | 1.6 | 1.0 | 2.4 |
|  | CDC COVID-19 Team | 3.9 | 3.3 | 4.7 |
|  | D+L pooled | 1.9 | 1.4 | 2.6 |
| Diseases of the digestive system |  |  |  |  |
|  | Du, R. et al. | 0.8 | 0.4 | 1.5 |
|  | D+L pooled | 0.8 | 0.4 | 1.5 |
| Hypertension |  |  |  |  |
|  | Du, R. et al. | 0.9 | 0.6 | 1.3 |
|  | Huang, C. et al. | 1.1 | 0.3 | 3.6 |
|  | Lei, S. et al. | 2.4 | 1.1 | 5.2 |
|  | Liu, Y. et al. | 1.3 | 0.9 | 1.9 |
|  | Wang, D. et al. | 3.1 | 1.8 | 5.4 |
|  | Wu, C. et al. | 1.6 | 1.1 | 2.2 |
|  | Simonnet A. et al. | 1.4 | 1.1 | 1.8 |
|  | Goyal, P. et al. | 1.2 | 0.9 | 1.5 |
|  | Kalligeros M. et al. | 1.3 | 0.8 | 2.2 |
|  | D+L pooled | 1.4 | 1.1 | 1.7 |
| Immunocompromised condition |  |  |  |  |
|  | CDC COVID-19 Team | 2.6 | 1.9 | 3.5 |
|  | D+L pooled | 2.6 | 1.9 | 3.5 |
| Myocardial infarction |  |  |  |  |
|  | Wu, C. et al. | 1.5 | 0.9 | 2.7 |
|  | D+L pooled | 1.5 | 0.9 | 2.7 |
| Other respiratory disease |  |  |  |  |
|  | Du, R. et al. | 0.5 | 0.2 | 1.1 |
|  | Kalligeros M. et al. | 1.4 | 0.9 | 2.2 |
|  | CDC COVID-19 Team | 2.6 | 2.1 | 3.2 |
|  | D+L pooled | 1.3 | 0.6 | 2.9 |

## Meta-analysis of Comorbidities and Death

| **Comorbidity** | **Study** | **RR** | **[95% Conf.**  **Interval]** | |
| --- | --- | --- | --- | --- |
| Acute renal failure |  |  |  |  |
|  | Xiao, G. et al. | 7.2 | 3.0 | 17.5 |
|  | D+L pooled | 7.2 | 3.0 | 17.5 |
| Asthma |  |  |  |  |
|  | Tomlins J. et al. | 0.9 | 0.3 | 2.4 |
|  | D+L pooled | 0.9 | 0.3 | 2.4 |
| Cancer |  |  |  |  |
|  | Chen, T. et al. | 1.8 | 1.1 | 2.9 |
|  | Deng G. et al. | 2.9 | 1.3 | 6.4 |
|  | The Novel Coronavirus Team | 2.5 | 1.1 | 5.4 |
|  | Yang, X. et al. | 0.8 | 0.2 | 3.3 |
|  | Yao Q. et al. | 4.8 | 1.1 | 21.5 |
|  | Zhang Jixiang. et al. | 1.9 | 0.3 | 13.3 |
|  | Nikpouraghdam M. et al. | 0.7 | 0.1 | 4.9 |
|  | D+L pooled | 2.0 | 1.4 | 2.8 |
| Cardiovascular Disease |  |  |  |  |
|  | Cao, J. et al. | 2.4 | 0.8 | 7.8 |
|  | Chen, T. et al. | 1.8 | 1.3 | 2.5 |
|  | Deng G. et al. | 6.7 | 5.4 | 8.4 |
|  | Fan J. et al. | 3.2 | 0.6 | 17.2 |
|  | Guan, W. et al. | 4.9 | 2.4 | 10.1 |
|  | Guo, T. et al. | 3.8 | 2.2 | 6.7 |
|  | Li, Y. et al. | 1.3 | 0.2 | 8.9 |
|  | The Novel Coronavirus Team | 5.0 | 4.0 | 6.1 |
|  | Wang Dawei et al. | 4.2 | 2.0 | 8.8 |
|  | Wang, L. et al. | 2.6 | 1.7 | 4.0 |
|  | Wang, Y. et al. | 1.5 | 1.1 | 2.1 |
|  | Yao Q. et al. | 5.2 | 1.7 | 16.3 |
|  | Zhang Jixiang. et al. | 5.4 | 2.4 | 12.0 |
|  | Zhou, F. et al. | 3.5 | 2.4 | 5.0 |
|  | Nikpouraghdam M. et al. | 1.3 | 0.5 | 3.4 |
|  | D+L pooled | 3.3 | 2.3 | 4.5 |
| Cerebrovascular Disease |  |  |  |  |
|  | Cao, J. et al. | 3.2 | 1.3 | 8.1 |
|  | Guan, W. et al. | 7.1 | 3.3 | 15.4 |
|  | Wang Dawei et al. | 3.2 | 1.3 | 7.9 |
|  | Yang, X. et al. | 1.7 | 1.2 | 2.3 |
|  | Wang, L. et al. | 2.8 | 1.7 | 4.6 |
|  | Yuan, M. et al. | 2.1 | 0.8 | 5.5 |
|  | Tomlins J. et al. | 1.2 | 0.3 | 4.3 |
|  | D+L pooled | 2.6 | 1.7 | 4.1 |
| Chronic obstructive pulmonary disease |  |  |  |  |
|  | Li, Y. et al. | 6.0 | 1.3 | 26.8 |
|  | Wang Dawei et al. | 1.9 | 0.4 | 10.1 |
|  | Wang, L. et al. | 3.1 | 1.9 | 5.0 |
|  | Wang, Y. et al. | 2.2 | 1.7 | 2.9 |
|  | Zhou, F. et al. | 2.5 | 1.3 | 4.6 |
|  | Tomlins J. et al. | 2.1 | 0.9 | 5.1 |
|  | D+L pooled | 2.4 | 2.0 | 3.0 |
| Chronic renal failure |  |  |  |  |
|  | Cheng, Y. et al. | 2.6 | 1.8 | 3.6 |
|  | Wang Dawei et al. | 1.9 | 0.4 | 10.1 |
|  | Nikpouraghdam M. et al. | 2.1 | 0.7 | 5.9 |
|  | D+L pooled | 2.5 | 1.8 | 3.4 |
| Diabetes mellitus |  |  |  |  |
|  | Cao, J. et al. | 2.4 | 0.9 | 5.9 |
|  | Chen, T. et al. | 1.3 | 0.9 | 1.8 |
|  | Deng G. et al. | 4.4 | 3.5 | 5.6 |
|  | Guan, W. et al. | 3.9 | 2.2 | 7.2 |
|  | Li, Y. et al. | 4.2 | 1.3 | 13.3 |
|  | The Novel Coronavirus Team | 3.4 | 2.7 | 4.2 |
|  | Wang Dawei et al. | 3.1 | 1.4 | 7.0 |
|  | Wang, L. et al. | 1.1 | 0.6 | 1.9 |
|  | Wang, Y. et al. | 1.3 | 0.9 | 1.7 |
|  | Wu, C. et al. | 2.7 | 1.6 | 4.6 |
|  | Yan Y. et al. | 1.7 | 1.4 | 2.1 |
|  | Yang, X. et al. | 1.3 | 0.9 | 2.1 |
|  | Yao Q. et al. | 1.9 | 0.3 | 11.8 |
|  | Yuan, M. et al. | 4.5 | 1.9 | 10.6 |
|  | Zhang Y. et al. | 3.9 | 0.4 | 39.3 |
|  | Zhou, F. et al. | 2.0 | 1.3 | 3.1 |
|  | Nikpouraghdam M. et al. | 1.2 | 0.7 | 2.2 |
|  | Tomlins J. et al. | 2.9 | 1.3 | 6.6 |
|  | D+L pooled | 2.2 | 1.7 | 2.9 |
| Diseases of liver |  |  |  |  |
|  | Guan, W. et al. | 1.1 | 0.2 | 8.0 |
|  | Wang Dawei et al. | 0.9 | 0.1 | 5.9 |
|  | Yao Q. et al. | 4.8 | 1.1 | 21.5 |
|  | D+L pooled | 1.9 | 0.6 | 6.4 |
| Diseases of the digestive system |  |  |  |  |
|  | Zhang Jixiang. et al. | 0.8 | 0.1 | 6.1 |
|  | D+L pooled | 0.8 | 0.1 | 6.1 |
| Endocrine diseases |  |  |  |  |
|  | Zhang Jixiang. et al. | 1.2 | 0.4 | 3.9 |
|  | D+L pooled | 1.2 | 0.4 | 3.9 |
| Hypertension |  |  |  |  |
|  | Cao, J. et al. | 3.3 | 1.5 | 7.5 |
|  | Chen, T. et al. | 1.8 | 1.4 | 2.3 |
|  | Deng G. et al. | 4.5 | 3.7 | 5.4 |
|  | Fan J. et al. | 1.1 | 0.2 | 6.4 |
|  | Guan, W. et al. | 6.3 | 3.6 | 10.8 |
|  | Li, Y. et al. | 2.9 | 0.6 | 14.9 |
|  | The Novel Coronavirus Team | 2.9 | 2.5 | 3.4 |
|  | Wang Dawei et al. | 3.5 | 1.6 | 7.6 |
|  | Wang, L. et al. | 1.4 | 0.9 | 2.2 |
|  | Wang, Y. et al. | 1.6 | 1.2 | 2.0 |
|  | Wu, C. et al. | 2.4 | 1.4 | 3.9 |
|  | Yao Q. et al. | 8.1 | 2.9 | 22.3 |
|  | Yuan, M. et al. | 3.8 | 1.8 | 8.3 |
|  | Zhang Y. et al. | 5.6 | 0.5 | 58.8 |
|  | Zhou, F. et al. | 2.1 | 1.4 | 3.3 |
|  | Nikpouraghdam M. et al. | 1.7 | 0.9 | 3.3 |
|  | Tomlins J. et al. | 2.1 | 1.0 | 4.6 |
|  | D+L pooled | 2.7 | 2.1 | 3.4 |
| Myocardial infarction |  |  |  |  |
|  | Wu, C. et al. | 2.4 | 1.1 | 5.1 |
|  | Yang, X. et al. | 1.0 | 0.5 | 2.1 |
|  | Yuan, M. et al. | 2.9 | 1.4 | 5.9 |
|  | Guo, T. et al. | 6.7 | 3.7 | 12.0 |
|  | Shi, S. et al. | 11.4 | 6.7 | 19.5 |
|  | D+L pooled | 3.6 | 1.5 | 8.6 |
| Other diseases or unspecified disease |  |  |  |  |
|  | Ruan, Q. et al. | 1.6 | 1.1 | 2.4 |
|  | Tang, N. et al. | 1.9 | 0.9 | 4.3 |
|  | D+L pooled | 1.7 | 1.2 | 2.4 |
| Other respiratory disease |  |  |  |  |
|  | Chen, T. et al. | 1.5 | 1.0 | 2.3 |
|  | Deng G. et al. | 3.4 | 2.4 | 4.9 |
|  | The Novel Coronavirus Team | 2.8 | 2.0 | 3.9 |
|  | Yang, X. et al. | 0.8 | 0.3 | 2.2 |
|  | Zhang Jixiang. et al. | 3.0 | 1.2 | 7.7 |
|  | Cao, J. et al. | 1.8 | 0.6 | 5.3 |
|  | Nikpouraghdam M. et al. | 1.9 | 1.0 | 3.5 |
|  | D+L pooled | 2.2 | 1.5 | 3.0 |

## Meta-analysis of Epidemiologic Factors and severe clinical course of disease

| **Epidemiologic Factor** | **Study** | **RR** | **[95% Conf.**  **Interval]** | |
| --- | --- | --- | --- | --- |
| Health care workers |  |  |  |  |
|  | Li X. et al. | 0.4 | 0.2 | 0.7 |
|  | Duanmu Y. et al. | 0.8 | 0.3 | 2.2 |
|  | McMichael, T. et al. | 0.1 | 0.0 | 0.3 |
|  | D+L pooled | 0.3 | 0.1 | 0.9 |
| Male |  |  |  |  |
|  | Cai, Q. et al. | 2.2 | 1.3 | 3.6 |
|  | Chen Q. et al. | 1.0 | 0.6 | 1.6 |
|  | Chen, G. et al. | 2.4 | 0.4 | 13.5 |
|  | Deng Q. et al. | 1.3 | 0.9 | 1.7 |
|  | Feng, Y. et al. | 1.4 | 1.0 | 2.0 |
|  | Ji D. et al. | 1.8 | 1.0 | 3.4 |
|  | Ji Dong. et al. | 1.8 | 1.0 | 3.3 |
|  | Li K. et al. | 1.3 | 0.7 | 2.6 |
|  | Li X. et al. | 1.3 | 1.1 | 1.5 |
|  | Li, Y. et al. | 2.2 | 0.7 | 6.8 |
|  | Liu, J. et al. | 1.9 | 0.8 | 4.7 |
|  | Liu, J. et al. | 1.4 | 0.6 | 3.2 |
|  | Ma J. et al. | 0.9 | 0.5 | 1.5 |
|  | Mao, L. et al. | 1.5 | 1.1 | 2.0 |
|  | Qin, C. et al. | 1.1 | 0.9 | 1.3 |
|  | Shi, Y. et al. | 2.4 | 1.3 | 4.5 |
|  | Sun S. et al. | 3.5 | 1.7 | 7.1 |
|  | Tian, S. et al. | 1.4 | 0.8 | 2.3 |
|  | Wan, S. et al. | 1.0 | 0.6 | 1.6 |
|  | Wang, X. et al. | 1.5 | 1.0 | 2.2 |
|  | Wei X. et al. | 1.3 | 1.0 | 1.7 |
|  | Wei YY. et al. | 1.5 | 0.8 | 3.0 |
|  | Xie, H. et al. | 1.4 | 0.8 | 2.7 |
|  | Xu, Y. et al. | 0.8 | 0.3 | 2.2 |
|  | Yao Q. et al. | 1.3 | 0.5 | 3.6 |
|  | Yu X. et al. | 2.0 | 1.0 | 4.2 |
|  | Zhang G. et al. | 1.8 | 1.1 | 3.0 |
|  | Zhang Jixiang. et al. | 1.1 | 1.0 | 1.2 |
|  | Zhang Y. et al. | 0.9 | 0.4 | 1.7 |
|  | Zhang, J. et al. | 1.3 | 0.9 | 1.9 |
|  | Zhang, J. et al. | 1.2 | 0.4 | 3.7 |
|  | Zhao X. et al. | 0.8 | 0.4 | 1.3 |
|  | Zheng S. et al. | 1.3 | 1.0 | 1.7 |
|  | Zheng, F. et al. | 0.9 | 0.5 | 1.7 |
|  | Zhu, Z. et al. | 2.3 | 0.9 | 5.9 |
|  | Zou Y. et al. | 3.1 | 1.3 | 7.4 |
|  | D+L pooled | 1.3 | 1.2 | 1.5 |
| Obesity |  |  |  |  |
|  | Zheng KI. et al. | 4.0 | 1.0 | 15.6 |
|  | Duanmu Y. et al. | 2.1 | 1.1 | 4.2 |
|  | D+L pooled | 2.4 | 1.3 | 4.4 |
| Smoking |  |  |  |  |
|  | Chen Q. et al. | 0.7 | 0.2 | 1.8 |
|  | Feng, Y. et al. | 1.5 | 1.0 | 2.3 |
|  | Ji D. et al. | 1.8 | 0.8 | 3.6 |
|  | Ji Dong. et al. | 1.1 | 0.4 | 2.8 |
|  | Li X. et al. | 1.2 | 1.0 | 1.4 |
|  | Li, Y. et al. | 3.2 | 1.2 | 8.6 |
|  | Liu, J. et al. | 1.3 | 0.4 | 4.1 |
|  | Liu, J. et al. | 1.9 | 0.7 | 5.6 |
|  | Liu, W. et al. | 5.5 | 2.1 | 14.4 |
|  | Qin, C. et al. | 0.7 | 0.3 | 1.6 |
|  | Shi, Y. et al. | 1.6 | 0.7 | 3.4 |
|  | Wan, S. et al. | 0.4 | 0.1 | 2.3 |
|  | Yu X. et al. | 1.2 | 0.6 | 2.4 |
|  | Zhang, J. et al. | 1.1 | 0.5 | 2.3 |
|  | Zou Y. et al. | 1.7 | 0.5 | 6.6 |
|  | Duanmu Y. et al. | 3.7 | 2.0 | 6.8 |
|  | CDC COVID-19 Team | 1.8 | 1.5 | 2.3 |
|  | D+L pooled | 1.5 | 1.2 | 1.9 |

## Meta-analysis of Epidemiologic Factors and ICU admission

| **Epidemiologic Factor** | **Study** | **RR** | **[95% Conf.**  **Interval]** | |
| --- | --- | --- | --- | --- |
| Male |  |  |  |  |
|  | Du, R. et al. | 1.135 | 0.725 | 1.778 |
|  | Guan, W. et al. | 1.474 | 0.898 | 2.419 |
|  | Huang, C. et al. | 2.017 | 0.529 | 7.693 |
|  | Lei, S. et al. | 0.714 | 0.312 | 1.635 |
|  | Liu, Y. et al. | 0.949 | 0.645 | 1.396 |
|  | Wang R. et al. | 1.352 | 0.648 | 2.823 |
|  | Wang, D. et al. | 1.32 | 0.739 | 2.358 |
|  | Wu, C. et al. | 1.426 | 0.979 | 2.077 |
|  | Simonnet A. et al. | 1.2 | 0.9 | 1.5 |
|  | Goyal, P. et al. | 1.6 | 1.1 | 2.2 |
|  | Kalligeros M. et al. | 1.2 | 0.8 | 2.0 |
|  | D+L pooled | 1.3 | 1.1 | 1.4 |
| Obesity |  |  |  |  |
|  | Goyal, P. et al. | 1.4 | 1.0 | 1.8 |
|  | Kalligeros M. et al. | 1.5 | 0.9 | 2.3 |
|  | D+L pooled | 1.4 | 1.1 | 1.8 |
| Smoking |  |  |  |  |
|  | Guan, W. et al. | 2.9 | 1.8 | 4.8 |
|  | Wang R. et al. | 2.6 | 1.3 | 5.3 |
|  | Goyal, P. et al. | 0.9 | 0.5 | 1.8 |
|  | Kalligeros M. et al. | 1.0 | 0.6 | 1.5 |
|  | CDC COVID-19 Team | 2.4 | 1.8 | 3.3 |
|  | D+L pooled | 1.8 | 1.1 | 2.9 |

## Meta-analysis of Epidemiologic Factors and death

| **Epidemiologic Factor** | **Study** | **RR** | **[95% Conf.**  **Interval]** | |
| --- | --- | --- | --- | --- |
| Health care workers |  |  |  |  |
|  | Chen, T. et al. | 0.1 | 0.0 | 0.8 |
|  | The Novel Coronavirus Team | 0.1 | 0.1 | 0.3 |
|  | D+L pooled | 0.1 | 0.1 | 0.3 |
| Male |  |  |  |  |
|  | Cao, J. et al. | 1.8 | 0.8 | 4.5 |
|  | Chen, T. et al. | 1.7 | 1.2 | 2.3 |
|  | Deng G. et al. | 1.6 | 1.5 | 1.9 |
|  | Fan J. et al. | 0.9 | 0.2 | 5.3 |
|  | Li, Y. et al. | 4.3 | 0.6 | 33.5 |
|  | Tang, N. et al. | 2.8 | 1.1 | 7.3 |
|  | The Novel Coronavirus Team | 1.7 | 1.5 | 1.9 |
|  | Wang Dawei et al. | 4.7 | 1.4 | 15.1 |
|  | Wang, L. et al. | 1.6 | 1.0 | 2.4 |
|  | Wang, Y. et al. | 1.2 | 0.9 | 1.5 |
|  | Wu, C. et al. | 1.1 | 0.6 | 1.9 |
|  | Yang, X. et al. | 0.9 | 0.6 | 1.4 |
|  | Yao Q. et al. | 2.1 | 0.7 | 6.2 |
|  | Yuan, M. et al. | 0.8 | 0.3 | 2.3 |
|  | Zhang Jixiang. et al. | 1.6 | 0.7 | 3.5 |
|  | Zhang Y. et al. | 0.7 | 0.1 | 7.5 |
|  | Zhou, F. et al. | 1.4 | 0.9 | 2.4 |
|  | Nikpouraghdam M. et al. | 1.2 | 0.9 | 1.6 |
|  | Tomlins J. et al. | 0.9 | 0.4 | 1.9 |
|  | D+L pooled | 1.4 | 1.3 | 1.6 |
| Smoking |  |  |  |  |
|  | Chen, T. et al. | 1.2 | 0.8 | 1.7 |
|  | Li, Y. et al. | 3.9 | 0.8 | 18.4 |
|  | Yao Q. et al. | 8.7 | 3.7 | 20.1 |
|  | Zhou, F. et al. | 1.7 | 0.8 | 3.3 |
|  | D+L pooled | 2.6 | 1.0 | 6.8 |

## References

1. Couper K, Taylor-Phillips S, Grove A, Freeman K, Osokogu O, Court R, et al. COVID-19 in cardiac arrest and infection risk to rescuers: a systematic review. Resuscitation [Internet]. 2020/04/24. 2020; Available from: https://www.ncbi.nlm.nih.gov/pubmed/32325096

2. Hu Y, Sun J, Dai Z, Deng H, Li X, Huang Q, et al. Prevalence and severity of corona virus disease 2019 (COVID-19): A systematic review and meta-analysis. J Clin Virol [Internet]. 2020/04/22. 2020;127:104371. Available from: https://www.ncbi.nlm.nih.gov/pubmed/32315817

3. Nasiri MJ, Haddadi S, Tahvildari A, Farsi Y, Arbabi M, Hasanzadeh S, et al. COVID-19 Clinical Characteristics, and Sex-Specific Risk of Mortality: Systematic Review and Meta-Analysis. Front Med [Internet]. 2020 Jul 21;7. Available from: https://www.frontiersin.org/article/10.3389/fmed.2020.00459/full

4. Xu L, Mao Y, Chen G. Risk factors for 2019 novel coronavirus disease (COVID-19) patients progressing to critical illness: a systematic review and meta-analysis. Aging (Albany NY) [Internet]. 2020 Jun 23;12(12):12410–21. Available from: https://www.aging-us.com/lookup/doi/10.18632/aging.103383

5. Zhao Q, Meng M, Kumar R, Wu Y, Huang J, Lian N, et al. The impact of COPD and smoking history on the severity of Covid-19: A systemic review and meta-analysis. J Med Virol [Internet]. 2020/04/16. 2020; Available from: https://www.ncbi.nlm.nih.gov/pubmed/32293753

6. Zheng Z, Peng F, Xu B, Zhao J, Liu H, Peng J, et al. Risk factors of critical & mortal COVID-19 cases: A systematic literature review and meta-analysis. J Infect [Internet]. 2020; Available from: https://www.ncbi.nlm.nih.gov/pmc/articles/PMC7177098/

7. Zuin M, Rigatelli G, Zuliani G, Rigatelli A, Mazza A, Roncon L. Arterial hypertension and risk of death in patients with COVID-19 infection: systematic review and meta-analysis. J Infect [Internet]. 2020/04/14. 2020; Available from: https://www.ncbi.nlm.nih.gov/pubmed/32283158

8. Li B, Yang J, Zhao F, Zhi L, Wang X, Liu L, et al. Prevalence and impact of cardiovascular metabolic diseases on COVID-19 in China. Clin Res Cardiol [Internet]. 2020/03/13. 2020; Available from: https://www.ncbi.nlm.nih.gov/pubmed/32161990

9. Matsushita K, Ding N, Kou M, Hu X, Chen M, Gao Y, et al. The Relationship of COVID-19 Severity with Cardiovascular Disease and Its Traditional Risk Factors: A Systematic Review and Meta-Analysis. Glob Heart [Internet]. 2020 Sep 22;15(1):64. Available from: https://globalheartjournal.com/article/10.5334/gh.814/

10. Aggarwal G, Lippi G, Michael Henry B. Cerebrovascular disease is associated with an increased disease severity in patients with Coronavirus Disease 2019 (COVID-19): A pooled analysis of published literature. Int J Stroke [Internet]. 2020/04/21. 2020;1747493020921664. Available from: https://www.ncbi.nlm.nih.gov/pubmed/32310015

11. Li JW, Han TW, Woodward M, Anderson CS, Zhou H, Chen YD, et al. The impact of 2019 novel coronavirus on heart injury: A systemic review and Meta-analysis. Prog Cardiovasc Dis [Internet]. 2020/04/20. 2020; Available from: https://www.ncbi.nlm.nih.gov/pubmed/32305557

12. Santoso A, Pranata R, Wibowo A, Al-Farabi MJ, Huang I, Antariksa B. Cardiac injury is associated with mortality and critically ill pneumonia in COVID-19: A meta-analysis. Am J Emerg Med [Internet]. 2020/04/26. 2020; Available from: https://www.ncbi.nlm.nih.gov/pubmed/32331955

13. Huang I, Lim M, Pranata R. Diabetes mellitus is associated with increased mortality and severity of disease in COVID-19 pneumonia - A systematic review, meta-analysis, and meta-regression. Diabetes Metab Syndr [Internet]. 2020;14(4):395–403. Available from: https://www.ncbi.nlm.nih.gov/pubmed/32334395

14. Cai Q, Huang D, Ou P, Yu H, Zhu Z, Xia Z, et al. COVID-19 in a Designated Infectious Diseases Hospital Outside Hubei Province, China. Allergy [Internet]. 2020/04/03. 2020;00:1–11. Available from: https://onlinelibrary.wiley.com/doi/epdf/10.1111/all.14309

15. Cao J, Tu W, Cheng W, Yu L, Liu Y, Hu X, et al. Clinical Features and Short-term Outcomes of 102 Patients with Corona Virus Disease 2019 in Wuhan, China. Clin Infect Dis [Internet]. 2020; Available from: https://academic.oup.com/cid/advance-article/doi/10.1093/cid/ciaa243/5814897

16. CDC COVID-19 Response Team. Preliminary Estimates of the Prevalence of Selected Underlying Health Conditions Among Patients with Coronavirus Disease 2019 — United States, February 12–March 28, 2020. MMWR Morb Mortal Wkly Rep. 2020;69(13):382–386.

17. Chen G, Zhao J, Ning Q. Clinical and immunological features of severe and moderate coronavirus disease 2019. J Clin Invest [Internet]. 2020; Available from: https://www.jci.org/articles/view/137244/pdf

18. Chen Q, Zheng Z, Zhang C, Zhang X, Wu H, Wang J, et al. Clinical characteristics of 145 patients with corona virus disease 2019 (COVID-19) in Taizhou, Zhejiang, China. Infection [Internet]. 2020/04/29. 2020; Available from: https://www.ncbi.nlm.nih.gov/pubmed/32342479

19. Chen T, Wu D, Chen H, Yan W, Yang D, Chen G, et al. Clinical characteristics of 113 deceased patients with coronavirus disease 2019: retrospective study. BMJ. 2020;368.

20. Cheng Y, Luo R, Wang K, Zhang M, Wang Z, Dong L, et al. Kidney disease is associated with in-hospital death of patients with COVID-19. Kidney Int [Internet]. 2020/04/06. 2020; Available from: https://www.kidney-international.org/article/S0085-2538(20)30255-6/fulltext

21. Deng G, Yin M, Chen X, Zeng F. Clinical determinants for fatality of 44,672 patients with COVID-19. Crit Care [Internet]. 2020/04/30. 2020;24(1):179. Available from: https://www.ncbi.nlm.nih.gov/pubmed/32345311

22. Deng Q, Hu B, Zhang Y, Wang H, Zhou X, Hu W, et al. Suspected myocardial injury in patients with COVID-19: Evidence from front-line clinical observation in Wuhan, China. Int J Cardiol [Internet]. 2020/04/16. 2020; Available from: https://www.ncbi.nlm.nih.gov/pubmed/32291207

23. Du R, Liu L, Yin W, Wang W, Guan L, Yuan M, et al. Hospitalization and Critical Care of 109 Decedents with COVID-19 Pneumonia in Wuhan, China. Ann Am Thorac Soc [Internet]. 2020; Available from: https://www.atsjournals.org/doi/abs/10.1513/AnnalsATS.202003-225OC

24. Duanmu Y, Brown IP, Gibb WR, Singh J, Matheson LW, Blomkalns AL, et al. Characteristics of Emergency Department Patients With COVID-19 at a Single Site in Northern California: Clinical Observations and Public Health Implications. Acad Emerg Med [Internet]. 2020/04/29. 2020; Available from: https://www.ncbi.nlm.nih.gov/pubmed/32344458

25. Fan J, Wang H, Ye G, Cao X, Xu X, Tan W, et al. Low-density lipoprotein is a potential predictor of poor prognosis in patients with coronavirus disease 2019. Metabolism [Internet]. 2020/04/23. 2020;154243. Available from: https://www.ncbi.nlm.nih.gov/pubmed/32320740

26. Feng Y, Ling Y, Bai T, Xie Y, Huang J, Li J, et al. COVID-19 with Different Severity: A Multi-center Study of Clinical Features. Am J Respir Crit Care Med [Internet]. 2020/04/11. 2020; Available from: https://www.ncbi.nlm.nih.gov/pubmed/32275452

27. Goyal P, Choi JJ, Pinheiro LC, Schenck EJ, Chen R, Jabri A, et al. Clinical Characteristics of Covid-19 in New York City. N Engl J Med [Internet]. 2020/04/18. 2020; Available from: https://www.ncbi.nlm.nih.gov/pubmed/32302078

28. Guan W, Liang W, Zhao Y, Liang H, Chen Z, Li Y, et al. Comorbidity and its impact on 1590 patients with Covid-19 in China: A Nationwide Analysis. Eur Respir J [Internet]. 2020 Mar 26;2000547. Available from: http://erj.ersjournals.com/lookup/doi/10.1183/13993003.00547-2020

29. Guan W, Ni Z, Hu Y, Liang W, Ou C, He J, et al. Clinical Characteristics of Coronavirus Disease 2019 in China. N Engl J Med [Internet]. 2020; Available from: https://www.nejm.org/doi/full/10.1056/NEJMoa2002032

30. Guo T, Fan Y, Chen C, Wu X, Zhang L, He T, et al. Cardiovascular Implications of Fatal Outcomes of Patients With Coronavirus Disease 2019 (COVID-19). JAMA Cardiol [Internet]. 2020; Available from: https://www.ncbi.nlm.nih.gov/pmc/articles/PMC7101506/

31. Huang C, Wang Y, Li X, Ren L, Zhao J, Hu Y, et al. Clinical features of patients infected with 2019 novel coronavirus in Wuhan, China. Lancet [Internet]. 2020;395:497–506. Available from: https://www.thelancet.com/journals/lancet/article/PIIS0140-6736(20)30183-5/fulltext

32. Ji D, Zhang D, Xu J, Chen Z, Yang T, Zhao P, et al. Prediction for Progression Risk in Patients with COVID-19 Pneumonia: the CALL Score. Clin Infect Dis [Internet]. 2020/04/10. 2020; Available from: https://www.ncbi.nlm.nih.gov/pubmed/32271369

33. Ji D, Qin E, Xu J, Zhang D, Cheng G, Wang Y, et al. Implication of non-alcoholic fatty liver diseases (NAFLD) in patients with COVID-19: a preliminary analysis. J Hepatol [Internet]. 2020/04/12. 2020; Available from: https://www.ncbi.nlm.nih.gov/pubmed/32278005

34. Kalligeros M, Shehadeh F, Mylona EK, Benitez G, Beckwith CG, Chan PA, et al. Association of Obesity with Disease Severity among Patients with COVID-19. Obes (Silver Spring) [Internet]. 2020/05/01. 2020; Available from: https://www.ncbi.nlm.nih.gov/pubmed/32352637

35. Lei S, Jiang F, Su W, Chen C, Chen J, Mei W, et al. Clinical characteristics and outcomes of patients undergoing surgeries during the incubation period of COVID-19 infection. EClinicalMedicine [Internet]. 2020/04/16. 2020;100331. Available from: https://www.ncbi.nlm.nih.gov/pubmed/32292899

36. Li K, Wu J, Wu F, Guo D, Chen L, Fang Z, et al. The Clinical and Chest CT Features Associated with Severe and Critical COVID-19 Pneumonia. Invest Radiol [Internet]. 2020/03/03. 2020; Available from: https://www.ncbi.nlm.nih.gov/pubmed/32118615

37. Li X, Xu S, Yu M, Wang K, Tao Y, Zhou Y, et al. Risk factors for severity and mortality in adult COVID-19 inpatients in Wuhan. J Allergy Clin Immunol [Internet]. 2020/04/16. 2020; Available from: https://www.ncbi.nlm.nih.gov/pubmed/32294485

38. Li Y, Peng S, Li L, Wang Q, Ping W, Zhang N, et al. Clinical and Transmission Characteristics of Covid-19 - A Retrospective Study of 25 Cases From a Single Thoracic Surgery Department . Curr Med Sci [Internet]. 2020; Available from: https://pubmed.ncbi.nlm.nih.gov/32232652/?from_term=covid+19+and+cases&from_sort=date&from_pos=3

39. Liang W, Guan W, Chen R, Wang W, Li J, Xu K, et al. Cancer patients in SARS-CoV-2 infection: a nationwide analysis in China. Lancet Oncol [Internet]. 2020/02/19. 2020;21(3):335–7. Available from: https://www.ncbi.nlm.nih.gov/pubmed/32066541

40. Liu J, Li S, Liu J, Liang B, Wang X, Wang H, et al. Longitudinal characteristics of lymphocyte responses and cytokine profiles in the peripheral blood of SARS-CoV-2 infected patients. EBioMedicine [Internet]. 2020 May;55:102763. Available from: https://linkinghub.elsevier.com/retrieve/pii/S2352396420301389

41. Liu J, Liu Y, Xiang P, Pu L, Xiong H, Li C, et al. Neutrophil-to-lymphocyte ratio predicts critical illness patients with 2019 coronavirus disease in the early stage. J Transl Med [Internet]. 2020 Dec 20;18(1):206. Available from: https://link.springer.com/content/pdf/10.1186/s12967-020-02374-0.pdf

42. Liu W, Tao Z, Wang L, Yuan M, Liu K, Zhou L, et al. Analysis of factors associated with disease outcomes in hospitalized patients with 2019 novel coronavirus disease. Chin Med J (Engl). 2020;

43. Liu Y, Sun W, Li J, Chen L, Wang Y, Zhang L, et al. Clinical features and progression of acute respiratory distress syndrome in coronavirus disease 2019. medRxiv. 2020.

44. Ma J, Yin J, Qian Y, Wu Y. Clinical Characteristics and Prognosis in Cancer Patients with COVID-19: a Single Center’s Retrospective Study. J Infect [Internet]. 2020/04/17. 2020; Available from: https://www.ncbi.nlm.nih.gov/pubmed/32298677

45. Mao L, Jin H, Wang M, Hu Y, Chen S, He Q, et al. Neurologic Manifestations of Hospitalized Patients With Coronavirus Disease 2019 in Wuhan, China. JAMA Neurol [Internet]. 2020/04/11. 2020; Available from: https://www.ncbi.nlm.nih.gov/pubmed/32275288

46. McMichael T, Currie D, Clark S, Pogosjans S, Kay M, Schwartz N, et al. Epidemiology of Covid-19 in a Long-Term Care Facility in King County, Washington. new engl J Med. 2020;

47. Nikpouraghdam M, Jalali Farahani A, Alishiri G, Heydari S, Ebrahimnia M, Samadinia H, et al. Epidemiological characteristics of coronavirus disease 2019 (COVID-19) patients in IRAN: A single center study. J Clin Virol [Internet]. 2020/05/01. 2020;127:104378. Available from: https://www.ncbi.nlm.nih.gov/pubmed/32353762

48. Qin C, Zhou L, Hu Z, Zhang S, Yang S, Tao Y, et al. Dysregulation of immune response in patients with COVID-19 in Wuhan, China. Clin Infect Dis. 2020;

49. Ruan Q, Yang K, Wang W, Jiang L, Song J. Clinical predictors of mortality due to COVID-19 based on an analysis of data of 150 patients from Wuhan, China. Intensive Care Med [Internet]. 2020/03/04. 2020; Available from: https://www.ncbi.nlm.nih.gov/pubmed/32125452

50. Shi Y, Yu X, Zhao H, Wang H, Zhao R, Sheng J. Host susceptibility to severe COVID-19 and establishment of a host risk score: findings of 487 cases outside Wuhan. Crit Care. 2020;24.

51. Simonnet A, Chetboun M, Poissy J, Raverdy V, Noulette J, Duhamel A, et al. High prevalence of obesity in severe acute respiratory syndrome coronavirus-2 (SARS-CoV-2) requiring invasive mechanical ventilation. Obes (Silver Spring) [Internet]. 2020/04/10. 2020; Available from: https://www.ncbi.nlm.nih.gov/pubmed/32271993

52. Sun S, Cai X, Wang H, He G, Lin Y, Lu B, et al. Abnormalities of peripheral blood system in patients with COVID-19 in Wenzhou, China. Clin Chim Acta [Internet]. 2020/04/28. 2020;507:174–80. Available from: https://www.ncbi.nlm.nih.gov/pubmed/32339487

53. Tang N, Li D, Wang X, Sun Z. Abnormal coagulation parameters are associated with poor prognosis in patients with novel coronavirus pneumonia. J Thromb Haemost [Internet]. 2020;18:844–847. Available from: https://onlinelibrary.wiley.com/doi/10.1111/jth.14768

54. Novel Coronavirus Pneumonia Emergency Response Epidemiology Team. The epidemiological characteristics of an outbreak of 2019 novel coronavirus diseases (COVID-19) - China, 2020. China CDC Wkly [Internet]. 2020/02/18. 2020;2. Available from: https://www.ncbi.nlm.nih.gov/pubmed/32064853

55. Tian S, Hu N, Lou J, Chen K, Kang X, Xiang Z, et al. Characteristics of COVID-19 infection in Beijing. J Infect [Internet]. 2020/03/01. 2020; Available from: https://www.ncbi.nlm.nih.gov/pubmed/32112886

56. Tomlins J, Hamilton F, Gunning S, Sheehy C, Moran E, Macgowan A. Clinical features of 95 sequential hospitalised patients with novel coronavirus 2019 disease (COVID-19), the first UK cohort. J Infect [Internet]. 2020/05/01. 2020; Available from: https://www.ncbi.nlm.nih.gov/pubmed/32353384

57. Wan S, Xiang Y, Fang W, Zheng Y, Li B, Hu Y, et al. Clinical features and treatment of COVID‐19 patients in northeast Chongqing. J Med Virol [Internet]. 2020;1–10. Available from: https://onlinelibrary.wiley.com/doi/full/10.1002/jmv.25783

58. Wang D, Yin Y, Hu C, Liu X, Zhang X, Zhou S, et al. Clinical course and outcome of 107 patients infected with the novel coronavirus, SARS-CoV-2, discharged from two hospitals in Wuhan, China. Crit Care [Internet]. 2020/05/02. 2020;24(1):188. Available from: https://www.ncbi.nlm.nih.gov/pubmed/32354360

59. Wang D, Hu B, Hu C, Zhu F, Liu X, Zhang J, et al. Clinical Characteristics of 138 Hospitalized Patients With 2019 Novel Coronavirus–Infected Pneumonia in Wuhan, China. JAMA [Internet]. 2020 Mar 17;323(11):1061. Available from: https://jamanetwork.com/journals/jama/fullarticle/2761044

60. Wang L, He W, Yu X, Hu D, Bao M, Liu H, et al. Coronavirus Disease 2019 in elderly patients: characteristics and prognostic factors based on 4-week follow-up. J Infect [Internet]. 2020/04/03. 2020; Available from: https://www.sciencedirect.com/science/article/pii/S0163445320301468

61. Wang R, Pan M, Zhang X, Fan X, Han M, Zhao F, et al. Epidemiological and clinical features of 125 Hospitalized Patients with COVID-19 in Fuyang, Anhui, China. Int J Infect Dis [Internet]. 2020/04/15. 2020; Available from: https://www.sciencedirect.com/science/article/pii/S1201971220302034

62. Wang X, Fang J, Zhu Y, Chen L, Ding F, Zhou R, et al. Clinical characteristics of non-critically ill patients with novel coronavirus infection (COVID-19) in a Fangcang Hospital. Clin Microbiol Infect [Internet]. 2020/04/07. 2020; Available from: https://www.clinicalmicrobiologyandinfection.com/article/S1198-743X(20)30177-4/fulltext

63. Wang Y, Lu X, Chen H, Chen T, Su N, Huang F, et al. Clinical Course and Outcomes of 344 Intensive Care Patients with COVID-19 [Internet]. American Journal of Respiratory and Critical Care Medicine. 2020. Available from: https://www.atsjournals.org/doi/10.1164/rccm.202003-0736LE

64. Wei X, Su J, Yang K, Wei J, Wan H, Cao X, et al. Elevations of serum cancer biomarkers correlate with severity of COVID-19. J Med Virol [Internet]. 2020/04/30. 2020; Available from: https://www.ncbi.nlm.nih.gov/pubmed/32347972

65. Wei YY, Wang RR, Zhang DW, Tu YH, Chen CS, Ji S, et al. Risk factors for severe COVID-19: evidence from 167 hospitalized patients in Anhui, China. J Infect [Internet]. 2020/04/20. 2020; Available from: https://www.ncbi.nlm.nih.gov/pubmed/32305487

66. Williamson EJ, Walker AJ, Bhaskaran K, Bacon S, Bates C, Morton CE, et al. Factors associated with COVID-19-related death using OpenSAFELY. Nature [Internet]. 2020 Aug 20;584(7821):430–6. Available from: http://www.nature.com/articles/s41586-020-2521-4

67. Wu C, Chen X, Cai Y, Xia J, Zhou X, Xu S, et al. Risk Factors Associated With Acute Respiratory Distress Syndrome and Death in Patients With Coronavirus Disease 2019 Pneumonia in Wuhan, China. JAMA Intern Med. 2020;

68. Xie H, Zhao J, Lian N, Lin S, Xie Q, Zhuo H. Clinical characteristics of Non‐ICU hospitalized patients with coronavirus disease 2019 and liver injury：A Retrospective study [Internet]. Liver International. 2020. Available from: https://onlinelibrary.wiley.com/doi/abs/10.1111/liv.14449

69. Xu Y, Dong J, An W, Lv X, Yin X, Zhang J, et al. Clinical and computed tomographic imaging features of novel coronavirus pneumonia caused by SARS-CoV-2. J Infect [Internet]. 2020; Available from: https://pubmed.ncbi.nlm.nih.gov/32109443/

70. Yan Y, Yang Y, Wang F, Ren H, Zhang S, Shi X, et al. Clinical characteristics and outcomes of patients with severe covid-19 with diabetes. BMJ Open Diab Res Care [Internet]. 2020;8:e001343. Available from: https://drc.bmj.com/content/bmjdrc/8/1/e001343.full.pdf

71. Yang X, Yu Y, Xu J, Shu H, Xia J, Liu H, et al. Clinical course and outcomes of critically ill patients with SARS-CoV-2 pneumonia in Wuhan, China: a single-centered, retrospective, observational study. Lancet Respir Med [Internet]. 2020/02/28. 2020; Available from: https://www.ncbi.nlm.nih.gov/pubmed/32105632

72. Yao Q, Wang P, Wang X, Qie G, Meng M, Tong X, et al. Retrospective study of risk factors for severe SARS-Cov-2 infections in hospitalized adult patients. Pol Arch Intern Med [Internet]. 2020/04/25. 2020; Available from: https://www.ncbi.nlm.nih.gov/pubmed/32329978

73. Yu X, Sun S, Shi Y, Wang H, Zhao R, Sheng J. SARS-CoV-2 viral load in sputum correlates with risk of COVID-19 progression. Crit Care [Internet]. 2020/04/25. 2020;24(1):170. Available from: https://www.ncbi.nlm.nih.gov/pubmed/32326952

74. Yuan M, Yin W, Tao Z, Tan W, Hu Y. Association of radiologic findings with mortality of patients infected with 2019 novel coronavirus in Wuhan, China. PLoS One. 2020;15:1–10.

75. Zhang G, Hu C, Luo L, Fang F, Chen Y, Li J, et al. Clinical features and short-term outcomes of 221 patients with COVID-19 in Wuhan, China. J Clin Virol [Internet]. 2020/04/21. 2020;127:104364. Available from: https://www.ncbi.nlm.nih.gov/pubmed/32311650

76. Zhang J, Liu P, Wang M, Wang J, Chen J, Yuan W, et al. The clinical data from 19 critically ill patients with coronavirus disease 2019: a single-centered, retrospective, observational study. Z Gesundh Wiss [Internet]. 2020/04/23. 2020;1–4. Available from: https://www.ncbi.nlm.nih.gov/pubmed/32318325

77. Zhang J, Wang X, Jia X, Li J, Hu K, Chen G, et al. Risk factors for disease severity, unimprovement, and mortality of COVID-19 patients in Wuhan, China. Clin Microbiol Infect [Internet]. 2020/04/19. 2020; Available from: https://www.ncbi.nlm.nih.gov/pubmed/32304745

78. Zhang J, Dong X, Cao Y, Yuan Y, Yang Y, Yan Y, et al. Clinical characteristics of 140 patients infected with SARS‐CoV‐2 in Wuhan, China. Allergy [Internet]. 2020 Feb 27;00:all.14238. Available from: https://onlinelibrary.wiley.com/doi/abs/10.1111/all.14238

79. Zhang Y, Qin L, Zhao Y, Zhang P, Xu B, Li K, et al. Interferon-induced transmembrane protein-3 genetic variant rs12252-C is associated with disease severity in COVID-19. J Infect Dis [Internet]. 2020/04/30. 2020; Available from: https://www.ncbi.nlm.nih.gov/pubmed/32348495

80. Zhao XY, Xu XX, Yin HS, Hu QM, Xiong T, Tang YY, et al. Clinical characteristics of patients with 2019 coronavirus disease in a non-Wuhan area of Hubei Province, China: a retrospective study. BMC Infect Dis [Internet]. 2020/04/30. 2020;20(1):311. Available from: https://www.ncbi.nlm.nih.gov/pubmed/32345226

81. Zheng KI, Gao F, Wang XB, Sun QF, Pan KH, Wang TY, et al. Obesity as a risk factor for greater severity of COVID-19 in patients with metabolic associated fatty liver disease. Metabolism [Internet]. 2020/04/23. 2020;154244. Available from: https://www.ncbi.nlm.nih.gov/pubmed/32320741

82. Zheng S, Fan J, Yu F, Feng B, Lou B, Zou Q, et al. Viral load dynamics and disease severity in patients infected with SARS-CoV-2 in Zhejiang province, China, January-March 2020: retrospective cohort study. BMJ [Internet]. 2020/04/23. 2020;369:m1443. Available from: https://www.ncbi.nlm.nih.gov/pubmed/32317267

83. Zheng F, Tang W, Li H, Huang YX, Xie YL, Zhou ZG. Clinical characteristics of 161 cases of corona virus disease 2019 (COVID-19) in Changsha. Eur Rev Med Pharmacol Sci [Internet]. 2020/04/10. 2020;24(6):3404–10. Available from: https://www.ncbi.nlm.nih.gov/pubmed/32271459

84. Zhou F, Yu T, Du R, Fan G, Liu Y, Liu Z, et al. Clinical course and risk factors for mortality of adult inpatients with COVID-19 in Wuhan, China: a retrospective cohort study. Lancet [Internet]. 2020; Available from: https://www.thelancet.com/pdfs/journals/lancet/PIIS0140-6736(20)30566-3.pdf

85. Zhu Z, Cai T, Fan L, Lou K, Hua X, Huang Z, et al. Clinical value of immune-inflammatory parameters to assess the severity of coronavirus disease 2019. Int J Infect Dis [Internet]. 2020/04/26. 2020; Available from: https://www.ncbi.nlm.nih.gov/pubmed/32334118

86. Zou Y, Guo H, Zhang Y, Zhang Z, Liu Y, Wang J, et al. Analysis of coagulation parameters in patients with COVID-19 in Shanghai, China. Biosci Trends [Internet]. 2020/05/01. 2020; Available from: https://www.ncbi.nlm.nih.gov/pubmed/32350161

87. Metlay JP, Waterer GW, Long AC, Anzueto A, Brozek J, Crothers K, et al. Diagnosis and Treatment of Adults with Community-acquired Pneumonia. An Official Clinical Practice Guideline of the American Thoracic Society and Infectious Diseases Society of America. Am J Respir Crit Care Med [Internet]. 2019 Oct 1;200(7):e45–67. Available from: https://www.atsjournals.org/doi/10.1164/rccm.201908-1581ST

88. Mandell LA, Wunderink RG, Anzueto A, Bartlett JG, Campbell GD, Dean NC, et al. Infectious Diseases Society of America/American Thoracic Society Consensus Guidelines on the Management of Community-Acquired Pneumonia in Adults. Clin Infect Dis [Internet]. 2007 Mar 1;44(Supplement_2):S27–72. Available from: http://academic.oup.com/cid/article/44/Supplement_2/S27/372079/Infectious-Diseases-Society-of-AmericaAmerican

89. The ARDS Definition Task Force. Acute Respiratory Distress Syndrome. JAMA [Internet]. 2012 Jun 20;307(23). Available from: http://jama.jamanetwork.com/article.aspx?doi=10.1001/jama.2012.5669

90. Wu Z, McGoogan JM. Characteristics of and Important Lessons From the Coronavirus Disease 2019 (COVID-19) Outbreak in China. JAMA [Internet]. 2020 Apr 7;323(13):1239. Available from: https://jamanetwork.com/journals/jama/fullarticle/2762130

91. World Health Organization. Clinical management of COVID-19: interim guidance [Internet]. 2020. Available from: https://www.who.int/publications/i/item/clinical-management-of-severe-acute-respiratory-infection-when-novel-coronavirus-(ncov)-infection-is-suspected
